# Readiness for professional practice among health professions education graduates: a systematic review

**DOI:** 10.3389/fmed.2024.1472834

**Published:** 2024-11-08

**Authors:** Katie Wynne, Felista Mwangi, Oyepeju Onifade, Omotola Abimbola, Fiona Jones, Julie Burrows, Marita Lynagh, Tazeen Majeed, Dileep Sharma, Elizabeth Bembridge, Michelle Stubbs, Carla Sunner, Jessica Bergmann, Tanmay Bagade, Bunmi S. Malau-Aduli

**Affiliations:** ^1^School of Medicine and Public Health, University of Newcastle, Callaghan, NSW, Australia; ^2^School of Nursing and Midwifery, University of Newcastle, Callaghan, NSW, Australia; ^3^University Library, University of Newcastle, Callaghan, NSW, Australia; ^4^Department of Rural Health, University of Newcastle, Tamworth, NSW, Australia; ^5^School of Health Sciences, University of Newcastle, Callaghan, NSW, Australia

**Keywords:** readiness to practice, work-ready, professional practice, healthcare training, health graduates

## Abstract

Readiness to practice is the state of being prepared and capable of engaging in professional activities in a specific field. Graduates of healthcare professions require a diverse set of skills, knowledge and attitudes to meet the demands of complex healthcare settings. This systematic review provides a comprehensive analysis of readiness for professional practice among graduates of health professions education. It encompasses a wide range of articles published between 2013 and 2024, incorporating various health professions and utilizing a combination of quantitative, qualitative, and mixed methods designs. The review identified 93 studies from 32 diverse countries. The review revealed that factors influencing readiness to practice, include individual capabilities, the workplace context, and educational provision. It also explored strategies to enhance readiness to practice. The findings underscore the significance of addressing challenges such as lack of confidence, stress, communication, time management, job satisfaction, clinical experience, academic workload, teaching quality, mentorship and curriculum design. This comprehensive analysis serves as a valuable resource for healthcare educators, policymakers, and practitioners seeking to optimize the preparedness of graduates for the complexities of contemporary healthcare environments. Future studies should explore the interactions between readiness to practice stakeholders’ perception of the educational curriculum, and the quality of support in the professional practice environment.

## Introduction

Readiness for professional practice, also referred to as readiness to practice (R2P), is an important component of a student’s transition to professional practice and goal of health professions education (HPE). R2P refers to the state of being prepared and capable of engaging in professional activities in a specific field (1). It is having the knowledge, skills, and judgement that is required to perform their role (1). Readiness in healthcare settings not only relates to clinical proficiency, which is the ability to competently apply clinical knowledge, skills, and judgement, but also the ability to navigate ethical dilemmas, work collaboratively in interprofessional teams, adapt to evolving technologies, demonstrate cultural competence, interpersonal skills, and a commitment to lifelong learning (2). Graduates of HPE programs, including medicine, nursing, allied health or other related disciplines, require a diverse set of skills, knowledge, and attitudes to meet the complex demands of contemporary healthcare settings (3). Optimal performance in healthcare settings requires professionals to provide high-quality, patient-centered care within dynamic and often unpredictable environments, thus it is important that graduates possess the skills to adapt to this environment (3). Furthermore, achieving readiness for professional practice involves a combination of academic instruction, clinical experience, and the cultivation of professional values. It is therefore essential for graduates to be equipped with the cognitive, technical, and interpersonal skills required to address the individual needs of patients and to contribute to the improvement of healthcare systems (4, 5).

### Readiness for practice skills and attributes according to health disciplines

Effective communication, critical thinking, global citizenship, teamwork, independence, problem-solving and information literacy have been identified as common, yet important, graduate-level attributes, irrespective of discipline (6). General attributes necessary for success identified across different health specializations include critical appraisal skills, an inquiring mind, teamwork skills, continuous learning, adaptability to change, and awareness of politics and directions of healthcare (7–9). Furthermore, as demonstrated in these previous studies, different health disciplines have a unique combination of skills and attributes that are unique to the disciplines and thus required for success by the graduates.

#### Nursing

Nursing graduates require a range of skills and attributes to be ready for practice. These include competencies in professionalism, communication, management of responsibilities, critical thinking, clinical knowledge, and technical skills (10). They should also be able to apply theoretical concepts to clinical practice, communicate with other health professionals and convey information to patients, whilst being self-aware (11, 12). Additional work-ready attributes essential for nursing graduates include an approachable attitude, knowledge of ward or unit culture and routines and an ability to work within highly specialized ward or unit environments (12). Cultural competence is also an important work-ready skill for graduate nurses; this ensures the provision of quality healthcare to patients (13).

Nursing graduates need to be prepared to care for patients within different settings and cultural dynamics including during critical times such as the COVID-19 pandemic (14). Therefore, nursing graduates may require additional education and training in specific areas to be better prepared (14, 15). It is important for nursing students to have opportunities for practical learning, close guidance, support, and timely feedback in clinical settings (16). Furthermore, nursing graduates should possess clinical competence, the ability to make clinical judgements, and the skills needed to provide high-quality nursing care (17). Overall, nursing graduates require a combination of theoretical knowledge, practical skills, and the ability to adapt to the clinical environment’s demands to be ready for practice.

#### Medicine

The need for medical graduates to acquire multiple skills and attributes to be ready for practice has been emphasized in many studies. Important skills and attributes include clinical competence, personal capability and confidence, understanding of role and responsibilities, individual resilience, and provision of adequate support and feedback (18). Studies have also highlighted the key skills and attributes required by a graduate doctor to enhance their R2P and contribute to improved patient health outcomes. According to Morrow et al. (19), adequate preparedness for basic clinical tasks and good communication skills to work effectively with patients and colleagues are important. Studies have also shown that leadership skills, critical thinking, conflict resolution, helping others, mutual responsibility, and team building skills are important for medical graduates to improve their performance in clinical practice, research, and teamwork (20). Other essential profession-related competencies include interpersonal competencies (communication and collaboration), cognitive skills (problem solving, critical thinking, and reflectivity), work-related skills (planning and time management), and professionalism (integrity, sense of responsibility, respect, and empathy) (21).

The transition to practice for a medical graduate signifies the beginning of independent performance of professional duties at the workplace which further translates to a high level of responsibility in decision-making regarding the patient’s health (22). The preparedness for practice of medical students is associated with their professional identity, teamwork experience, and objective clinical rotation endpoints, such as clinical rotation results (23). Therefore, medical graduates need technical competence, communication skills, and consultation skills for gathering and transferring information to patients, which is crucial for accurate diagnosis, promoting patient understanding and adherence to recommendations (24).

#### Pharmacy

Pharmacists are a fundamental part of the healthcare system and possess a unique body of knowledge and skills which is essential in optimizing patient health outcomes (25). It is therefore important to ensure that pharmacy graduates are well prepared and equipped with the necessary work readiness skills required to be employable (25). Communication and leadership skills have been identified as the most important attributes for pharmacy students to be ready for practice in integrated health systems (26). Additional skills and attributes required by pharmacy graduates to be ready for practice include organizational competence, clarity of roles and responsibilities, team dynamics, self-awareness, and self-learning. Additionally, dispensing skills, prescription interpretation, patient counselling, pharmaceutical care, public health-related activities, and administrative and management skills are essential work-ready attributes a graduate pharmacist should possess (27). Pharmacy graduates are expected to have forward-thinking, patient-centric approach to practice and a provider mentality, thus being able to meet regulatory requirements as care providers (28).

#### Physiotherapy

Several attributes are described as important for physiotherapy graduates’ preparedness for the workforce. A study in Australia on the work readiness of new graduate physiotherapists showed that stakeholders perceived new graduates to be partially prepared for practice thus emphasizing the need for additional specific attributes and skills to enhance their transition into the workforce (29).

Work-ready attributes necessary for physiotherapy graduates include clinical skills such as manual therapy, red flag management, and exercise prescription (30). Attributes such as knowledge of anatomy and human function, workplace injury prevention and rehabilitation and disability management are also necessary (31). Physiotherapy graduates also need to develop psychosocial skills, patient management, and effective communication with patient relatives, and the multidisciplinary team (32). In addition to these clinical skills, graduates should possess attributes such as confidence in their own abilities, escalation, and communication. They should have a solid educational foundation that includes good attitudes and skills to develop their professional practice (33, 34). Furthermore, graduates should be competent in assessing and managing pain, demonstrating empathic and compassionate communication, and understanding patient preferences (35). Therefore, it is necessary that physiotherapy graduates acquire these attributes to ensure they are prepared to meet workforce demands and provide high-quality patient care.

#### Nutritionists and dietitians

Work-ready attributes for nutrition and dietetics graduates include a combination of personal characteristics, general skills, and specific competencies. Employers in the nutrition and dietetics field value attributes such as motivation, completion of tasks, dependability, and respectfulness which are important for success in the workplace (36, 37). Additional work-ready attributes for nutritionists and dietitians include a positive attitude, flexibility, good listening skills, professionalism, in-depth knowledge of nutrition, enhancing career profile, and personal interest in the field (36, 38). Furthermore, competencies including critical thinking, problem-solving, oral/written communication, teamwork, collaboration, and digital technology skills are highly regarded by employers and are critical for graduates to effectively contribute to their roles (39). Therefore, possessing these work-ready attributes is crucial for nutrition and dietetics graduates to thrive in their professional careers and provide high-quality services.

#### Other allied health disciplines

Allied health graduates, including speech pathologists, occupational therapists, social workers, radiographers, clinical psychologists, exercise physiologists, chiropractors, podiatrists, and oral health therapists, require a range of work-ready skills to ensure their smooth transition into professional practice. Studies have identified the different skills and attributes required by a range of allied health professions, and these include developing relationships, planning and organization skills, clinical reasoning, interprofessional practice and self-confidence (40). Additional attributes graduates should possess include interpersonal capabilities, communication skills, self-awareness, organizational acumen, resilience, and professionalism (41–43). These attributes encompass a combination of personal characteristics, generic skills, and specific competencies vital for success in the allied health profession.

As healthcare systems worldwide undergo profound transformations, HPE programs face the critical task of ensuring that their graduates are not only well-versed in the latest disciplinary knowledge but are also equipped with the adaptive and interdisciplinary skills necessary to thrive in diverse and dynamic healthcare environments (22, 44). The evolving landscape of healthcare further underscores the importance of ensuring that HPE graduates are well-prepared for the realities they will face in their careers. Rapid advancements in medical technology, changes in healthcare policies, and the increasing emphasis on patient-centered care, require professionals who are not only competent in their respective disciplines but also adaptable and capable of lifelong learning (1). Moreover, the shift toward collaborative, interprofessional, team-based care emphasizes the need for graduates to communicate effectively, collaborate with colleagues from various disciplines, and contribute to a holistic approach to patient wellbeing (45).

While the existing literature underscores the importance of adaptive interdisciplinary skills for HPE graduates to thrive in dynamic, real world working environments, it does not provide a comprehensive and systematic evaluation of their readiness for professional practice. This gap in the literature necessitates a systematic review of recent studies on the professional readiness of HPE graduates. Consequently, this study seeks to address this gap by systematically reviewing the professional readiness (R2P) among HPE graduates. The review will focus on the conceptualization of R2P, the tools used for measurement, the factors that influence it, and the strategies proposed to enhance it. This approach will highlight the areas that need further exploration and contribute to a more nuanced and holistic understanding of professional practice readiness among HPE graduates.

## Methods

The Preferred Reporting Items for Systematic Reviews and Meta-Analysis (PRISMA) was adhered to in designing and preparing this study (46).

### Search strategy

Articles published in English between 2013 and 2024, a date range which the literature has focused on the gap between academic preparation and R2P, and that addressed R2P across health professions were included in the review. The literature search was conducted across five electronic databases: Medline, Scopus, CINAHL, PsycInfo, and EMBASE using identified keywords and indexed terms. The keywords included health professions terms such as: “health occupation,” “allied health,” “chiropractic,” “dentistry,” “medicine” or “nursing” and “preparedness” terms such as “ready,” “readiness” adjacent to professional practice terms like “practice” or “work” combined with Boolean operators (AND, OR).

### Study design

Articles included in the review were a combination of quantitative, qualitative and mixed-methods studies.

### Study selection

All studies identified by the search databases were retrieved and exported into Covidence (Veritas Health Innovation) (47), an online tool for study selection and data extraction, and duplicates were removed. The first phase of study identification was conducted by two independent reviewers (OO and BMA) who assessed the relevance of each study by title and abstract against the inclusion criteria to determine the need for full-text review. If either reviewer identified a potentially eligible study for full-text extraction, the full-text was retrieved. The second phase of the study identification was an assessment of full-text review of retrieved studies, completed by two independent reviewers (FM and BMA) to determine if they met inclusion criteria. In cases where reviewers disagreed, a third reviewer (OO) was consulted to reach consensus on suitability of the study for inclusion.

### Eligibility criteria

#### Participants/population

Studies were included whose target population was health professions students, graduates, and stakeholders such as nurses, doctors, dentists, oral health therapists, nutritionists and dietitians, physiotherapists, radiographers, chiropractors, podiatrists, biomedical scientists, clinical psychologists, exercise physiologists, pharmacists, speech pathologists, social workers, occupational therapists, and veterinary doctors.

#### Inclusion criteria

Studies included in this review were those conducted among health professions students, graduates and stakeholders, that investigated R2P, and were published in the English language between 2013 and May 2024. Included studies reported skills acquired by health professions graduates that prepare them for professional practice, described the conceptualization of R2P, reported tools used to measure R2P or the factors influencing R2P, and described the strategies used to enhance R2P.

#### Exclusion criteria

Protocols for ongoing studies with no form of evaluation of outcomes reported, studies reporting R2P of graduates of other professions, conference abstracts, opinion papers, clinical case studies, book chapters, editorials, commentaries, all types of reviews, and studies not published in English language or available as translation in English were excluded from this review.

### Data extraction and synthesis

Data extraction was conducted by two independent reviewers (OA and BMA) and cross-checked by a third independent reviewer (OO) for accuracy and consistency. Key data reported from each study included the names of authors, aim of the study, study site, discipline, definition of R2P according to the article, conceptualization of R2P as implied in the article, measurement tool used, factors influencing R2P and strategies to enhance R2P. Due to the heterogeneity of the studies included, a meta-analysis could not be conducted. Thus, the readiness for professional practice among health professions graduates was described in a narrative synthesis.

### Risk of bias assessment

The Quality Assessment for Diverse Studies (QuADS) was used to assess the quality of the included papers (48). The QuADS tool was chosen due to the demonstrated interrater reliability, content, and face validity, and is more appropriate to assess the quality of multi- or mixed-methods research (48). The tool has a total of 13 assessment criteria, with rating scores ranging from zero (0-not stated at all) to three (3-explicitly described/completely appropriate) (48). Ten authors were involved in the assessment, with each study independently assessed by a pair of these authors. The risk of bias for each paper was then determined by calculating the average of the assessments made by each pair. This approach ensured a balanced and unbiased evaluation of each study.

## Results

### Study selection

**Figure 1:**
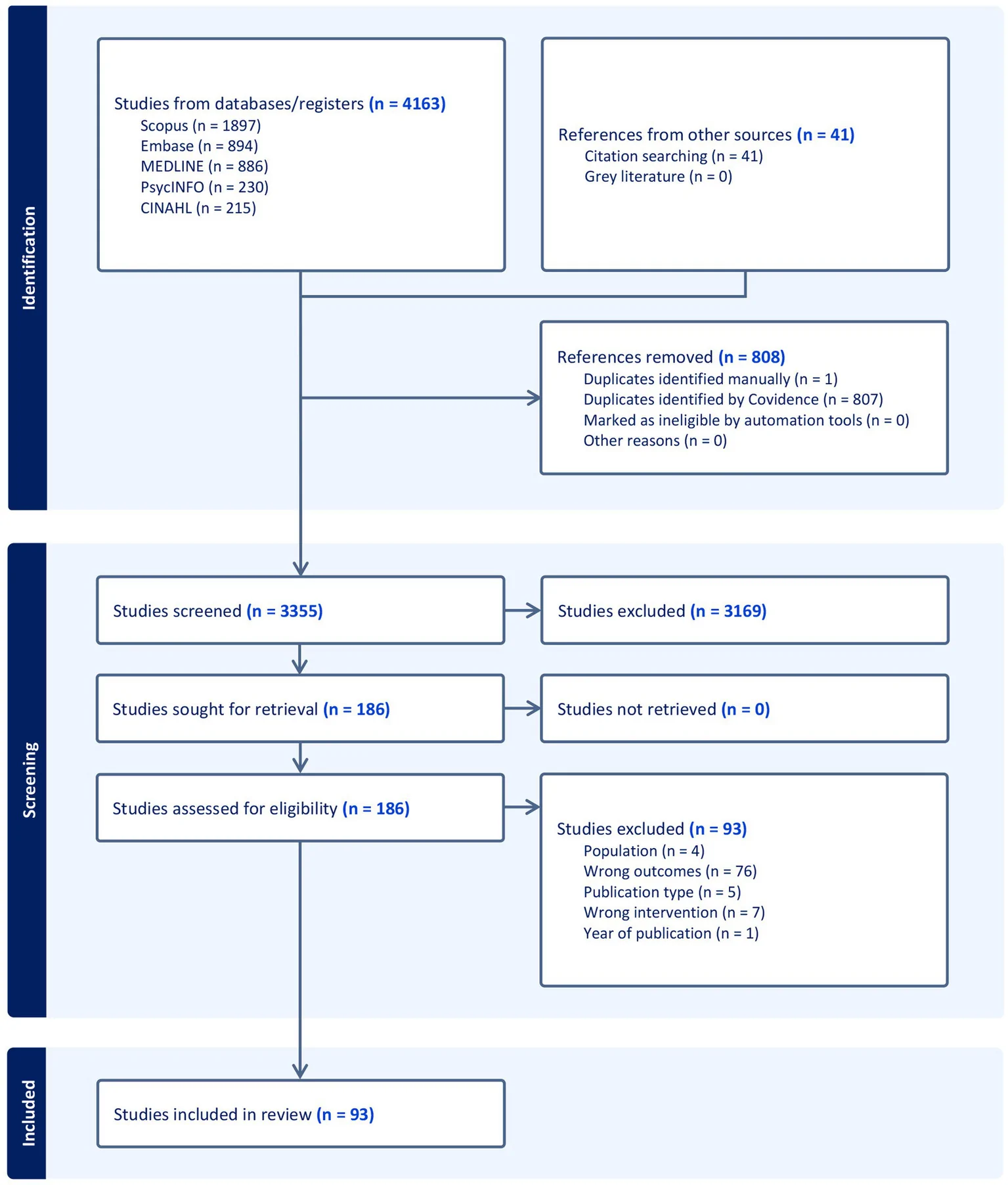
PRISMA chart.

A total of 4,163 studies were identified from the initial database search with 3,355 articles identified after duplicates were removed (Figure 1). During the title and abstract screening, 3,169 records were excluded. The remaining 186 full-text records were screened, and a further 93 studies were excluded. Reasons for exclusion varied and they include wrong population, wrong outcomes, wrong interventions and publication type and year of publication. The majority of the studies were excluded for wrong outcomes (n = 76) as the studies were out of this review’s focus. Ninety-three articles remained and were included in this study. No additional records were identified by hand-searching the reference lists of included articles.

### Characteristics of included studies

**Figure 2:**
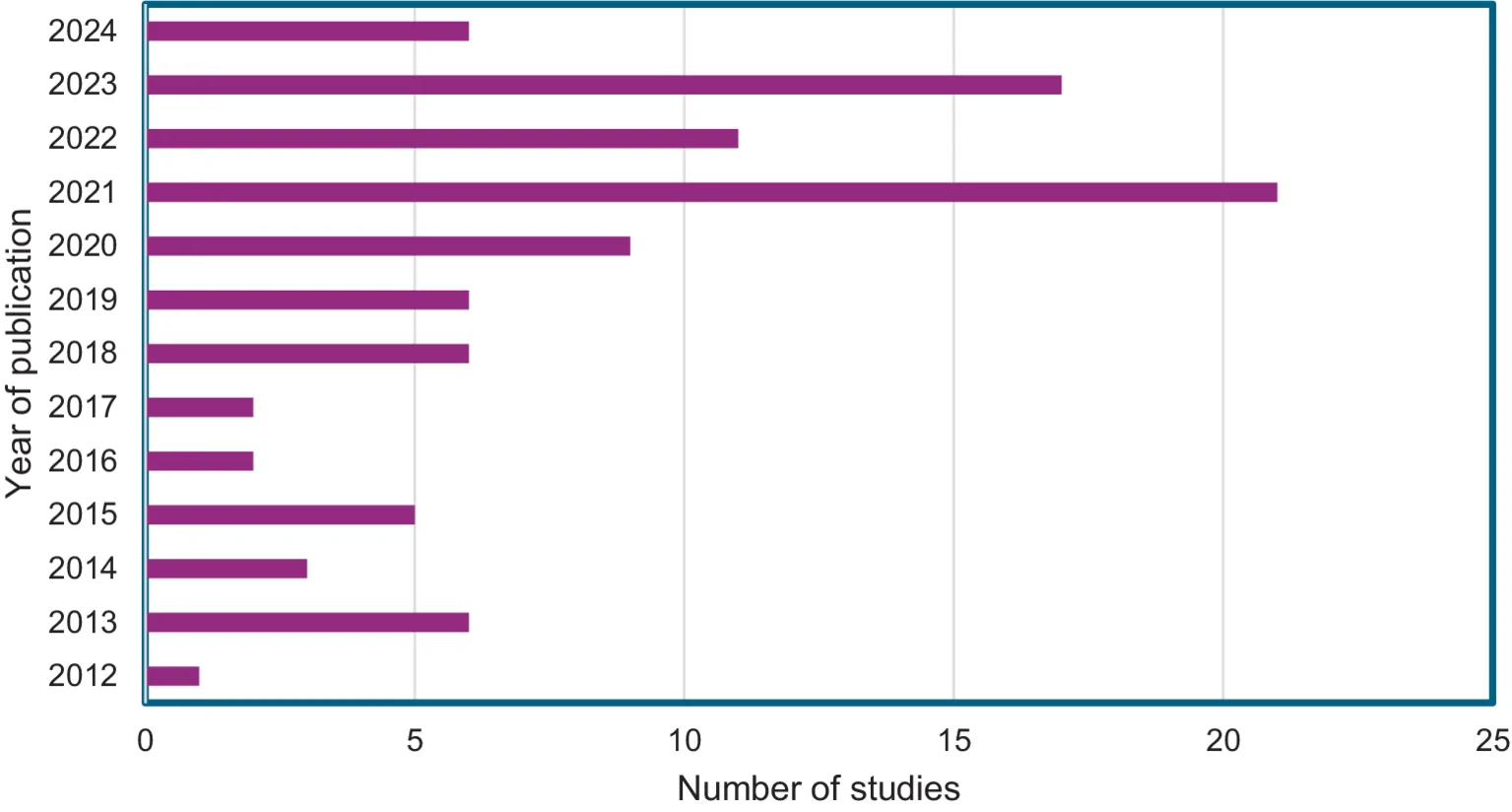
Publication years of included studies to May 2024.

Thirty-nine (42%) of the 93 articles were quantitative studies, 30 articles (32%) were qualitative and 24 (26%) were mixed methods studies (Supplementary Table S1), with most of the studies published within the last 5 years (Figure 2). The majority of studies focused on post-graduates (47%) or undergraduates (29%) or both post-graduates and undergraduates (5%); a smaller number included faculty and educators (5%) or employers (2%) and the remainder mixed groups of stakeholders.

### Countries

**Figure 3:**
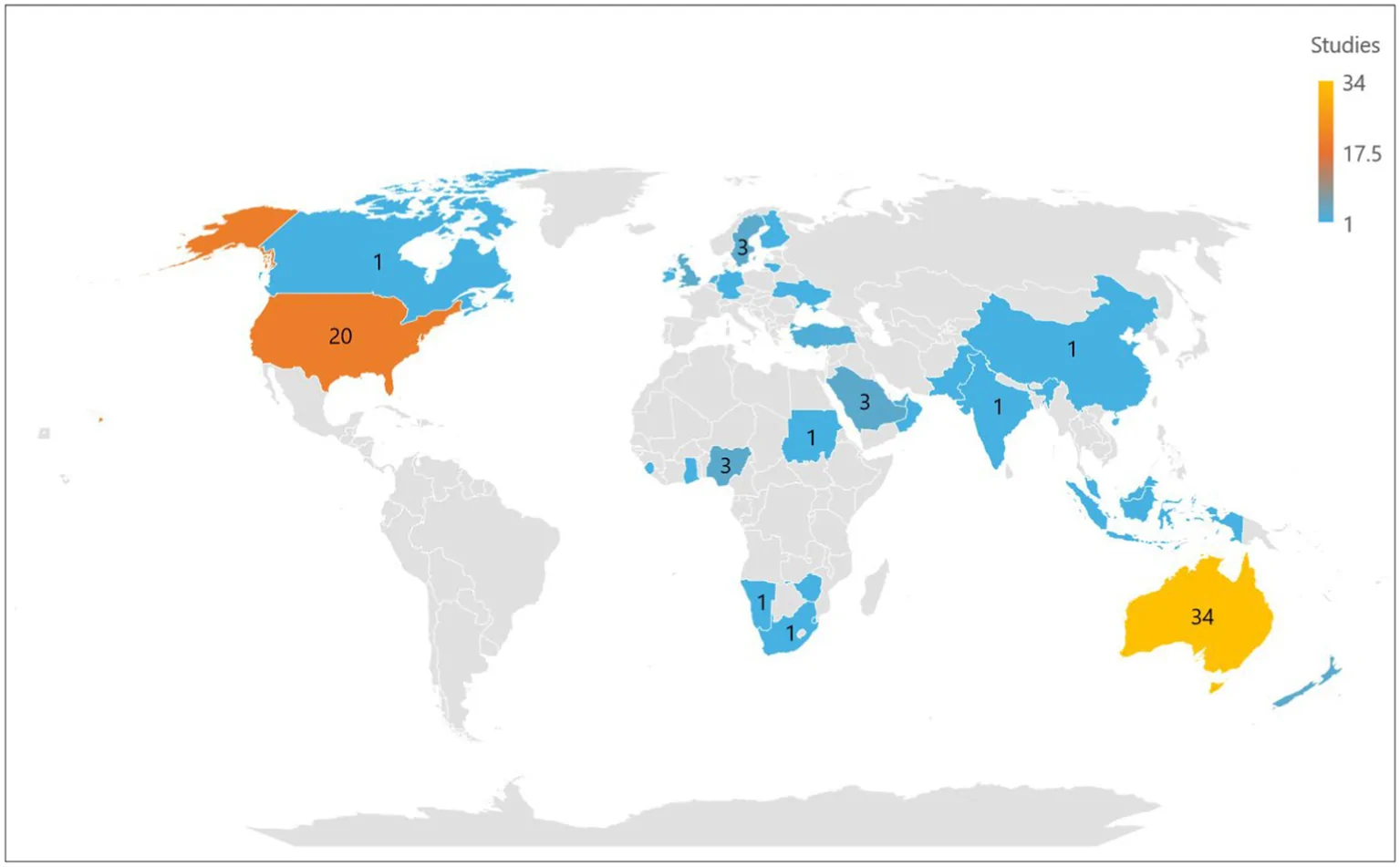
Geographic distribution of the 93 articles included in the literature review.

Of the 93 articles reviewed, 32 (34%) were from Australia and 19 (20%) were from the United States. The United Kingdom, New Zealand, Nigeria, Sweden and Saudi Arabia had three studies (3%) each, Turkey and United Arab Emirates had two studies (2%) each while India, Korea, Ghana, Singapore, Pakistan, Sierra Leone Swaziland, Scotland Canada, Zimbabwe, Ukraine, South Africa, Namibia, Netherlands, Oman, Ireland, Indonesia, Malaysia and China had one study (1%) each (Figure 3). Four studies were conducted in multiple countries: Netherlands and Germany; Finland and Lithuania; Australia and Singapore; and Australia, Sudan and the United States of America.

### Health professions

**Figure 4:**
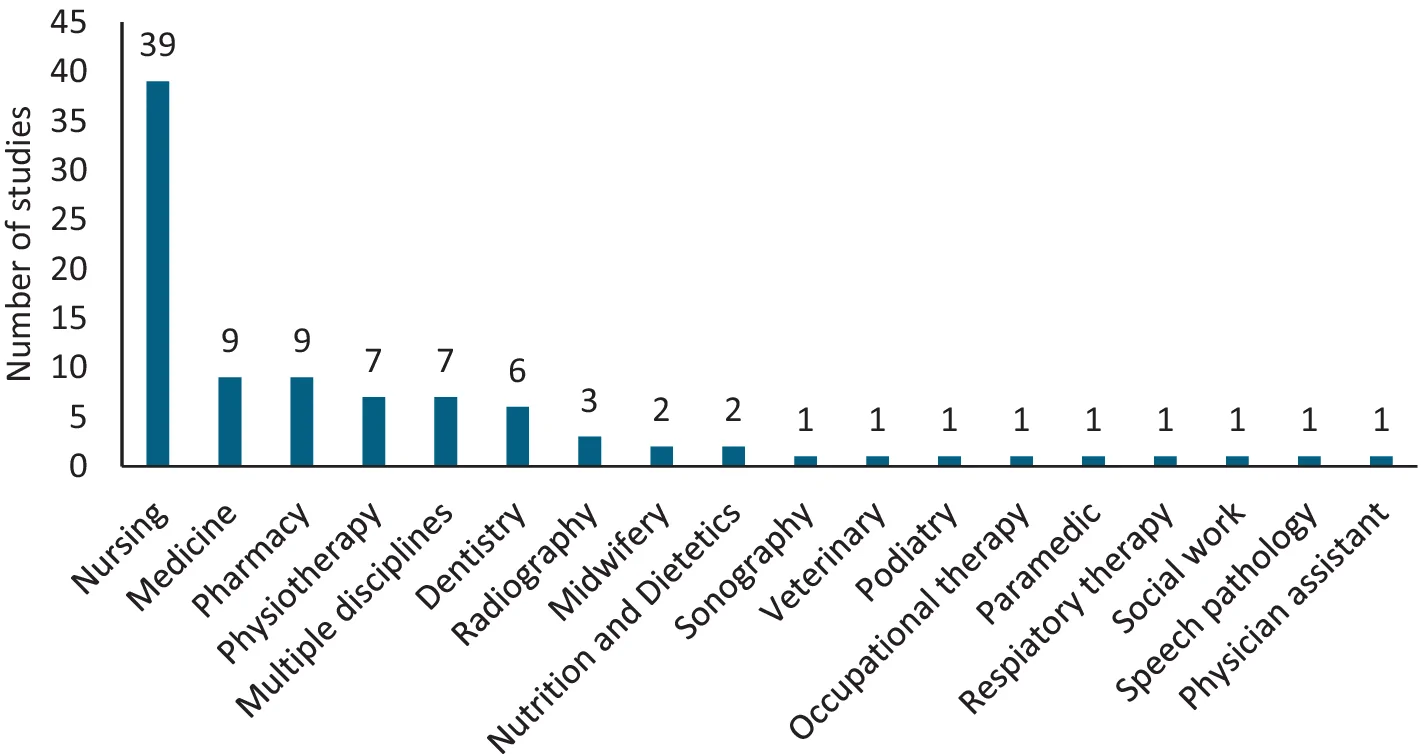
Health professions in the included studies.

Of the 93 articles, 39 were focused on the nursing profession (42%), nine each on pharmacy and medicine (10%), seven on physiotherapy (8%), six on dentistry (6%), three studies on radiography (3%) and two each on nutrition & dietetics, and midwifery (2%). There were single studies on the physician assistant, podiatry, respiratory therapy, social work, sonography, speech pathology, paramedicine, occupational therapy, and veterinary medicine (1%) (Figure 4).

### Conceptualization of readiness for professional practice

Readiness for professional practice (R2P) was conceptualized as a multifaceted construct that encompasses different components influencing graduates’ preparedness to transition into the workforce and subsequently perform their roles efficiently (36, 49). These factors include clinical skills and knowledge, interpersonal skills, critical thinking, adaptability, and a commitment to lifelong learning (50–52). The conceptualization of R2P fluctuates across different settings and researchers have sought to define and operationalize readiness in a way that captures the complexity of the transition from education to practice (53, 54).

As shown in Supplementary Table S2, R2P has been defined as the extent to which graduates are perceived to possess the attitudes and attributes that make them prepared or ready for success in the workplace (12, 52, 55). The study by Malau-Aduli et al. (4) showed that R2P is an emerging work of literature that focuses on graduate readiness and the factors that enhance or are barriers to R2P, and relies on the utilization of case-based learning, research, practical skills development, and interprofessional activities (4). Other studies defined R2P in the context of professional practice and a graduate’s ability to perform efficiently in a new role different from the school environment. Attrill and colleagues defined R2P as skills and attributes required to allow graduates to succeed in the workplace (56). Studies also provided definitions for R2P in the context of acquisition of skills necessary for efficient performance in the workplace. It was defined as the “preparedness for a lifetime working within a field that is likely to change significantly over the lifespan of the graduate” (57). Additionally, Pullen and Ahchay (49) succinctly described R2P as the immediate ability to excel in professional roles upon graduation. It is the result of training encompassing knowledge and skills for effective teamwork, patient-centered care delivery, and the ability to adapt to the dynamic demands of a working environment, and a blend of competencies and attributes that graduates are believed to possess upon entering the workforce (58–61).

### Measuring readiness for professional practice

The common tools used for measuring R2P were surveys (83%), interviews (49%) and focus groups (8%); mostly as a combination of two tools to obtain in-depth information on the skills and attributes needed for work readiness of health graduates. Supplementary Table S2 shows that the frequently used standard scales in the surveys were the Work Readiness Scale (55, 62, 63) and the Casey Fink Graduate Nurse Experience Survey (64–66). Only the Graduate Work Readiness Project Survey tool (45) and the Work Readiness Scale (62, 63, 67) were used to evaluate R2P across multiple health professions.

### Factors that influence readiness for professional practice

New graduates encounter challenges when transitioning into professional practice despite acquiring foundational skills during their training (53). Several factors were reported to contribute to and influence healthcare professionals’ R2P. These factors were broadly categorized into individual, educational, and contextual factors (Supplementary Table S2 and Table 1), and they were reported in 76 (82%), 88 (95%) and 66 (71%) of the reviewed studies, respectively.

**Table 1:** Summary of factors that influence readiness.

Domain	Factor
Individual	Self-confidence
	Adaptability
	Resilience
	Competency
	Good communication skills
	Critical thinking
	Stress management
	Professional skills
Educational	Access to mentorship
	Optimal curriculum design
	Realistic simulation
	Quality clinical placement
	Effective feedback
	Interprofessional learning opportunities
Contextual	Supportive workplace culture and activities
	Realistic workload
	Available resources
	Quality workplace supervision
	Orientation programs
	Workplace familiarity

#### Individual factors

New health graduates must develop confidence in managing complex clinical situations to be ready for professional practice (68). As evidenced in 30 studies (Supplementary Table S2), self-confidence was among the most important individual factors determining graduates’ preparedness for professional practice (29, 51, 55, 69, 70). Graduates reported low or no confidence in important skills such as central line care (16), handling professional challenges (71), performing complex dental procedures (72) and managing multiple patient assignments (68). Studies reported that health professions graduates need to be adaptable (17%) (58, 73–75), resilient (12%) (76–79), and competent (11%) (2, 80, 81, 147) to be prepared for practice. Furthermore, graduates require good communication (12%) (25, 43, 67, 82), critical thinking (83, 84), stress management (67, 84, 85), and professional skills (58, 67, 86) to succeed in their roles after graduation.

#### Educational factors

Educational factors that were reported as critical for graduates’ R2P included mentorship (29%), optimal curriculum design (23%), and clinical placements (23%). Studies reported that students are better prepared for practice by curricula directly aimed at equipping students with the required skills for employment (27, 32, 64). Opportunities for quality clinical placement were reported as particularly important due to the experience of hands-on practice (32) (4, 57) and exposure to real-world scenarios (87, 88). Furthermore, interprofessional learning opportunities (89), effective feedback mechanisms (16, 87) and the realism of simulations (90) may influence graduates’ R2P.

#### Contextual factors

New graduates reported reality shock upon entry into professional practice due to the excess workload, impediments to patient care, the burden of student supervision, and lack of professional trust (91). Studies reported that workplace culture (16%) (92–94), availability of resources (10%) (69, 95, 96), workload during training or transition to practice (4, 80, 97, 98) and orientation (58, 59) influence graduates readiness for their professional roles. Quality workplace supervision (93) and a supportive workplace culture (56), participation in workplace activities (58), orientation programs (59) and workplace familiarity were reported to provide a sense of belonging and minimize stress and feelings of overwhelm.

### Strategies to enhance readiness for professional practice

As portrayed in Supplementary Table S2, evidence from this review showed that effective strategies for enhancing R2P require a multifaceted approach that addresses the individual, educational, and contextual factors influencing practice readiness.

#### Curricular design

Eighty-six studies in this review (93%) reported that curriculum design is an important strategy to enhance readiness for professional practice. These strategies targeted clinical and professional competencies (57%), improved simulation practices (38%), and ensured regular assessment and feedback practices (20%). Strategies to develop clinical and professional competencies include enhancing clinical placements, improving clinical training quality, improving internship programs, and providing more clinical exposure (49, 71, 99, 100). Improving quality of simulations, promoting rural placements that extend patient responsibility, enhancing practical training, increasing real-life practical exposures and hands-on training are useful approaches in the overall enhancement of R2P (50, 54, 58, 87, 101–103). Additionally, structured feedback and assessment mechanisms were identified as effective methods to improve curriculum design. The studies showed that providing regular feedback, regular and continuous assessment mechanisms, and incorporating feedback from standardized assessors into curriculum are important strategies to improve curriculum design (64, 82, 90, 100, 104).

#### Individual support and capabilities

Of the 93 studies in this review, over half (52%) identified various individual support mechanisms as important strategies, and almost half (48%) showed that mentorship and guidance are factors that can improve R2P. The studies reported that providing continuous support and robust mentorship, regular support networks, and enhanced and supportive clinical supervision could significantly improve readiness for professional practice (3, 5, 32, 53, 61, 79, 82, 105). Furthermore, lifelong learning and self-reflection were identified as strategies to enhance R2P. Some studies in this review (3%) showed that encouraging self-assessment, prioritization skills and enhanced focus on time management and critical thinking could enhance R2P (36, 83, 86). Furthermore, fostering professional behaviors and attitudes in the new graduates could increase R2P (106).

#### Workplace integration

Of the studies in this review, almost a third (30%) reported various workplace integration mechanisms that could enhance graduates’ R2P. The main mechanisms identified include a supportive and inclusive workplace environment (16%), structured orientation (6%), and collaboration and teamwork amongst professionals (6%).

Providing consistent and comprehensive structured orientation could enhance R2P (49, 52, 58). Individualized attention during transition to the workplace (52), collaboration between educational institutions and industry, increased group dynamics and teamwork exercises (101), and supportive collegial environments (63, 92, 107) provide quality support that improves new graduates’ R2P.

### Risk of bias of included studies

The risk of bias assessment results are presented in Table 2. The scores ranged from 35 to 97%. There were more high-quality studies (n = 46) compared to medium (n = 42) and low-quality studies (n = 5). Most studies scored between zero and one on stakeholder engagement in the research. Description of study aims, study design, format and content of the data collection tool and statistical analysis had the highest scores.

**Table 2:** Risk of bias assessment results of the reviewed studies.

	QuADS criteria^*^													
Study (Year)	1	2	3	4	5	6	7	8	9	10	11	12	13	Maximum possible score (%)
Pullen and Ahchay (49)	1	3	2	2	1	3	2	3	2	2	2	3	2	71
Akinkugbe et al. (101)	3	3	2	3	2	3	3	3	2	3	3	2	3	88
Grimm and Barker (90)	3	3	3	3	3	2	3	3	2	1	3	0	2	74
Stulz et al. (105)	0	1	2	2	2	0	1	2	2	1	1	0	1	36
Ersoy and Ayaz-Alkaya (50)	1	2	2	2	3	3	3	3	2	3	3	0	3	68
Watt and Pascoe (88)	1	2	3	3	3	1	3	3	1	0	3	0	2	58
Zhang et al. (52)	1	2	2	3	2	1	2	1	2	3	3	2	2	59
Mak et al. (60)	0	3	3	2	3	2	3	3	3	3	3	3	1	78
Graham et al. (71)	0	3	2	2	2	1	2	2	2	3	3	3	3	64
Sheehan et al. (54)	3	1	3	3	1	3	3	2	2	3	3	2	0	64
Oluwatosin and Ogundero (36)	0	2	2	2	2	2	2	2	1	2	2	2	0	47
Reynolds and Mclean (100)	3	3	3	3	3	3	3	3	3	0	3	2	2	86
Farris et al. (87)	3	3	3	3	2	2	3	3	2	3	3	3	3	88
Woolley et al. (103)	3	3	3	3	3	3	3	3	3	3	3	3	3	97
Hatzenbuhler and Klein (99)	2	3	3	3	2	1	3	2	1	2	2	0	2	64
Lagali-Jirge and Umarani (72)	0	3	1	1	1	1	1	1	2	2	2	1	1	38
Hyun et al. (82)	3	3	3	2	3	3	2	3	3	2	2	3	3	86
Ottrey et al. (61)	3	3	3	3	3	3	3	3	1	3	3	0	3	83
Phan et al. (53)	1	3	3	3	2	2	3	3	3	3	3	2	3	79
Gruenberg et al. (58)	3	3	2	3	3	3	3	2	3	3	3	1	3	87
Ford et al. (104)	2	2	3	3	3	3	3	3	3	3	3	2	3	91
Opoku et al. (79)	2	2	3	3	3	3	3	3	3	3	3	1	2	85
Lim et al. (59)	3	3	3	3	3	3	3	3	3	3	2	2	3	94
Harrison et al. (3)	2	3	3	3	2	2	3	2	2	3	3	1	2	79
Mustakallio et al. (102)	3	3	3	3	3	3	3	2	2	0	3	2	2	82
Javed et al. (127)	0	3	3	3	3	3	3	3	3	3	3	0	3	81
Smith et al. (128)	2	3	2	2	2	3	2	2	2	2	2	0	1	54
Almarzoky Abuhussain et al. (62)	2	2	1	2	3	2	2	2	2	2	2	1	1	59
Adam et al. (92)	1	3	3	3	3	3	3	3	3	3	3	0	2	85
Almadani et al. (64)	0	3	3	3	3	2	3	2	2	2	2	1	1	68
AlMekkawi and El Khalil (16)	1	3	3	3	3	3	3	3	3	1	1	1	2	77
Almotairy et al. (107)	2	3	2	2	3	2	3	2	3	3	3	0	3	69
Al-Rawajfah et al. (129)	1	3	3	3	3	3	3	3	3	3	3	3	2	87
Anokwuru and Daniels (73)	0	3	3	3	2	3	3	3	1	2	2	0	1	67
Atkinson and McElroy (130)	0	3	2	3	3	1	2	2	2	3	3	1	2	64
Attrill et al. (56)	3	3	3	3	3	3	3	3	1	3	3	0	3	86
Bäck et al. (80)	2	3	3	2	3	3	3	2	3	2	2	2	3	85
Barr et al. (2)	2	2	3	3	1	3	3	2	3	3	3	0	2	74
Bradley et al. (81)	1	3	2	2	2	2	2	2	1	2	3	1	2	64
Carter and Stoehr (131)	3	3	3	3	2	2	3	2	3	1	3	0	3	74
Casey et al. (76)	1	3	3	3	3	3	3	3	3	3	3	0	3	87
Chesterton et al. (32)	2	3	3	3	3	3	3	3	3	3	3	3	3	96
Clark et al. (93)	2	3	1	3	3	3	3	3	3	3	3	3	2	90
Dlamini et al. (132)	1	3	2	2	2	1	2	2	2	2	2	1	0	53
Dudley et al. (133)	2	3	3	3	1	2	3	2	1	3	3	0	2	72
Duijn et al. (97)	3	3	3	2	2	3	3	3	3	1	3	2	3	87
Fejzic and Barker (25)	2	3	3	3	1	0	0	2	3	0	0	0	3	50
Fenech et al. (67)	2	3	2	3	2	3	3	3	1	2	3	0	1	71
Forbes and Ingram (89)	1	2	2	3	3	2	3	3	3	3	3	0	3	78
Friedlander et al. (95)	0	3	2	2	2	2	2	3	1	2	2	1	3	64
Grant et al. (77)	2	3	3	2	3	2	2	3	3	2	2	1	2	74
Haruzivishe and Macherera (74)	1	2	3	3	1	2	3	3	3	0	3	0	1	60
Harvey et al. (134)	0	3	3	2	2	2	2	2	2	1	2	0	0	54
Illing et al. (98)	3	3	3	3	3	3	3	3	3	3	3	3	2	97
James and Cole (27)	1	3	3	3	1	2	3	3	3	2	3	0	3	74
Jamieson et al. (65)	3	3	3	3	3	3	3	3	3	2	2	2	1	87
Kasita et al. (91)	1	2	3	3	3	2	2	3	2	1	2	1	1	60
Kinnane et al. (106)	1	3	2	3	3	2	3	3	3	2	3	0	1	73
Kuzmenko et al. (22)	1	2	2	1	1	1	1	2	2	1	2	1	1	42
Lanahan et al. (75)	2	2	2	3	2	3	3	3	2	2	3	0	3	73
Lazarus et al. (135)	1	3	3	3	3	3	3	3	2	3	3	2	3	86
Leufer and Cleary-Holdforth (136)	1	1	2	1	1	1	1	2	2	2	1	0	0	35
Li et al. (94)	3	3	3	3	3	3	3	3	3	3	3	0	3	92
Malau-Aduli et al. (4)	3	3	3	3	3	3	3	3	3	3	3	3	3	95
Mariño et al. (137)	3	3	3	3	0	3	3	3	2	2	3	2	3	85
Meyer and Shatto (78)	3	3	3	3	3	3	3	3	3	3	3	0	1	87
Missen et al. (138)	1	3	3	3	3	1	2	3	3	3	3	0	2	73
Monrouxe et al. (5)	3	3	3	3	3	3	3	3	1	3	3	3	3	91
Muruvan et al. (15)	1	3	2	3	3	3	3	3	1	3	3	0	3	79
Musallam and Flinders (68)	2	3	3	3	1	3	3	2	0	0	2	0	2	62
Nelson et al. (139)	2	2	2	3	2	1	3	2	0	2	3	1	2	63
Nweke et al. (69)	3	3	2	2	2	2	2	2	2	2	2	2	3	74
O'Brien et al. (57)	1	3	3	3	2	2	3	2	3	3	3	0	2	65
O'Brien et al. (43)	2	3	3	3	3	2	3	3	2	2	3	0	0	64
Phillips et al. (140)	2	3	3	3	2	2	2	3	3	3	3	2	3	83
Piccuito and Santiago (96)	2	2	3	3	2	2	3	2	2	2	3	0	2	72
Powers et al. (85)	2	3	2	2	3	2	2	3	3	3	3	2	3	85
Rusch et al. (86)	0	3	3	3	3	2	3	3	3	2	3	0	3	78
Shaw et al. (83)	2	3	3	3	3	3	3	3	3	2	3	2	1	86
Sterner et al. (141)	2	3	3	3	3	2	3	3	2	3	3	3	3	90
Stoikov et al. (142)	2	3	3	3	3	2	2	2	2	2	2	0	2	72
Tarhan et al. (143)	3	3	3	3	3	3	3	3	3	3	3	0	3	91
Thomas and Merrill (84)	3	2	3	3	0	3	3	3	3	3	3	0	3	81
Usher et al. (70)	2	3	3	3	3	3	3	3	3	3	3	1	3	87
Waite et al. (144)	0	3	3	3	3	3	3	3	3	1	3	2	2	82
Walker and Campbell (63)	2	3	2	3	3	3	3	2	2	3	3	0	3	79
Walker et al. (12)	2	2	3	3	2	2	3	2	0	2	3	0	2	65
Walters et al. (55)	3	3	3	3	2	3	3	1	3	3	3	0	2	79
Wells et al. (29)	1	3	3	3	3	3	3	3	3	2	3	3	2	86
Wijnen-Meijer et al. (145)	1	3	3	3	3	3	3	3	1	2	3	2	2	79
Willman et al. (51)	2	3	3	3	0	3	3	3	3	3	3	0	2	79
Wong et al. (146)	2	3	3	3	2	3	3	3	3	3	3	2	3	87
Woods et al. (66)	2	3	3	3	2	2	3	3	3	3	3	1	2	81

*QuADS Criteria: (1) theoretical or conceptual framework; (2) statement of research aims/objectives; (3) research setting and target population; (4) study design; (5) sampling; (6) rationale for choice of data collection tool/s; (7) format and content of data collection tool; (8) data collection procedure; (9) recruitment data; (10) justification for analytical method selected; (11) method of analysis appropriate; (12) research stakeholders considered in research design or conduct; (13) strengths and limitations. 0 = no mention at all, 1 = general reference/slightly appropriate; 2 = evidence of consideration/moderately appropriate; 3 = detailed description provided/completely appropriate (48).

## Discussion

This systematic review synthesized evidence from studies that investigated the readiness of health graduates for professional practice. The study highlights the essential components of R2P for successful practice in healthcare settings, which includes clinical proficiency, and a range of interpersonal and cognitive skills. The 93 studies included in this review originated from 32 countries. The review identified skills and attributes beneficial for graduates’ R2P and found that multiple individual, educational, and contextual factors were influential. Furthermore, the findings also underscore strategies used to enhance R2P, demonstrating that curricular design, individual support, and workplace integration are instrumental in ensuring readiness for professional practice.

Findings from this review showed that the various components of R2P have multiple benefits for graduates, employers and the healthcare system. Clinical skills and knowledge were identified as essential requirements for graduates to possess to provide high-quality care to patients. Similarly, there is existing evidence highlighting the benefits of graduate clinical skills and knowledge, showing that clinical skills acquired through work-integrated learning, volunteering, or shadowing prepares graduates for the real-world, contributes to high-quality care delivery and effective navigation of the healthcare environment (61).

Developing the interpersonal skills of communication and teamwork are crucial in working with patients, their families and other healthcare professionals (108). The potential for interpersonal communication to improve collaboration and patient safety and enhance patient-centered care underscores the need for healthcare graduates to acquire this skill. Evidence suggests that interpersonal communication, considered as one of the foundations of quality patient care, facilitates the establishment of a trust relationship between medical professionals and patients, thus contributing to a genuine therapeutic connection (109). This ensures a successful outcome of individualized nursing care, ensuring patient satisfaction and the protection of the health professional (109, 110). Studies conducted among nursing graduates show that to form this relationship, nurses need to understand and help their patients through a demonstration of courtesy, kindness, and sincerity. It also showed that nurses need to devote time to patient communication with utmost confidentiality and, by extension, to the people surrounding the sick individual (109).

The benefits of lifelong learning among healthcare professionals have been established in previous studies (111–113). Lifelong learning, a process where healthcare professionals continuously search for knowledge and understanding, ensures that they stay current with developments, thus enabling them to provide best practice evidence-based care to patients (113). Findings from this review support encouraging lifelong learning to enable graduates to stay up to date with the latest developments in healthcare and continue to improve their skills and knowledge throughout their careers (114). This leads to ongoing professional development, improved patient outcomes, and a more effective and efficient healthcare system (115). Additionally, in staying up to date with the latest developments, it is important for healthcare graduates to adopt innovative technological advancements to update their knowledge and skills (111). Evidence suggests that in the age of increasing influence of generative Artificial Intelligence (AI) on human behavior in learning and work, there is need for learning purpose to shift from focusing solely on human capital to promote competencies and capabilities in the era of AI (111).

There are significant implications for using tools to measure R2P in healthcare professions. The stress and uncertainty associated with transitioning into the workforce in a demanding and stressful healthcare environment, particularly among new graduates, can be mitigated by higher levels of perceived R2P (4). Identifying and utilizing the necessary tools needed to determine readiness, can facilitate thoughtful reflection among healthcare leaders and help identify gaps allowing for proactive resource allocations and preparatory activity to improve readiness (116). In this review, several tools were identified that are used to measure R2P across different professions, thereby offering comprehensive insights into the skills and attributes essential for work readiness for health graduates. Evidence from the review suggests that results of the assessment of R2P using the necessary tools, can inform the design and implementation of HPE programs (117). Furthermore, it suggests that the ability of the assessment to identify areas of strength and weakness in graduates’ preparedness allows the opportunity for targeted interventions to enhance readiness for professional practice (118).

Understanding factors that influence R2P can inform the design and implementation of HPE programs to improve graduates’ readiness (119). Evidence has shown that health professionals’ R2P in healthcare professions is influenced by a combination of factors related to their knowledge, skills, attitude, and the resources available in their work environment (4, 42, 120, 148). Similarly, in this review, factors influencing R2P could be broadly categorized into individual, educational and contextual domains. Identifying factors that are common across health professions may drive interprofessional learning and assessment and enhance collaboration in a multidisciplinary workforce.

Individual factors including burnout, stress, reality shock, anxiety, and case overload have been reported to negatively influence graduates’ R2P. A review on nurses’ preparedness for practice found that graduates experienced a “reality shock” in their transitioning to nursing role, with the reality of practice being challenging during transitioning, resulting in a feeling of being overwhelmed (119). Furthermore, it demonstrated a perceived disconnect between the ideal view of nursing from students and the real world of nursing encountered in professional practice (119). Similar studies also found that new graduates found heavy workload to be stressful as they struggled to adjust to shift work (119, 121, 122). Additionally, educational factors including academic workload can influence graduates’ R2P practice in healthcare (4, 42, 115, 119). Previous studies showed that academic workload particularly during placement was perceived as a barrier to learning key clinical and technical skills necessary for professional practice. This creates uncertainty in transitioning into the workforce as practice-ready health professionals (4, 79). Thus, it is necessary to ensure that academic workloads are efficiently managed to support the successful transition of newly graduated healthcare professionals to professional practice. Furthermore, Malau-Aduli et al. (4) showed that the perception of R2P is strongly linked to teaching quality, including the utilization of case-based learning, research and practical skills development, and inter-professional learning activities.

In addition to individual and educational factors, contextual factors may significantly influence R2P. These factors include workplace conflicts, culture, social, political, and economic conditions that influence the successful preparation, transition, and integration of new graduates into the workforce (4, 149). Common workplace conflicts impacting R2P include conflict with patients and families, conflicts that arise when healthcare professionals disagree on technical procedure or patient care decisions, and interprofessional conflicts which could be due to differing opinions, a hierarchical power structure and resource competition (123, 124). The potential of workplace conflicts to lead to a toxic work environment, which could ultimately affect performance and patient outcomes, highlights the imperative for newly graduated healthcare professionals to be equipped with conflict resolution and effective escalation skills that enhance their opportunities to optimize their work environment.

The need to identify strategies to enhance R2P among newly graduated healthcare professionals has been highlighted by previous studies (44). This review has broadly categorized these strategies into curricular design, individual support, and workplace integration. Involving practicing clinicians in curriculum design provides a contemporary and real-life perspective, thereby fostering innovation and enhancing students’ readiness (125). The use of competency-based curriculum design, informed by current literature and practitioners, is essential for creating health professional programs that effectively prepare graduates for practice (126). Similarly, quality clinical placements, simulations, interprofessional education, and effective feedback have been identified as significant contributors to enhancing readiness for graduates (4, 118). Quality clinical placements are essential for preparing healthcare graduates for practice, playing a crucial role in providing students with real-world settings to apply their knowledge and skills (4). Additionally, simulation-based learning has been acknowledged as an effective method for developing the skills and competencies of nursing students, further contributing to their overall readiness for clinical practice (118). In this systematic review, factors such as mentorship, supervision, teamwork, collaboration, structured orientation, and onboarding programs were identified as effective strategies to enhance R2P. Thus, this systematic review highlights the importance of creating supportive work environments, developing interprofessional collaboration, and creating and maintaining comprehensive onboarding programs in preparing new healthcare graduates for professional practice.

### Strengths and limitations

This systematic review used a robust methodology for the search strategy, data extraction and analysis and included a substantial number of papers published across diverse health disciplines, with a wide geographical distribution supporting the generalizability of the findings. The theoretical framework and conceptualization of R2P were included as outcomes. The authors are from a range of healthcare professions, have expertise in R2P, and are well-placed to design the study and interpret the results. The conclusion expands the existing literature and knowledge of R2P across healthcare professions.

The included professions, allied health, dentistry, medicine, nursing, pharmacy, veterinary medicine, are a broad representation of health professions, but are not universally inclusive of all healthcare providers. Older publications may not represent contemporary clinical competency or workplace expectations. Most papers were from nursing literature, potentially skewing the conclusions toward the nursing discipline. Grey literature and non-peer reviewed studies were not included and there may have been relevant information within these study types.

### Research gaps and future perspectives

This review acknowledges challenges in assessing readiness for professional practice, including the need for standardized assessments and effective feedback addressing variability in educational programs and ensuring ongoing professional development. Future directions include delivering studies that longitudinally track new graduates’ success in practice, refining R2P assessment tools and enabling education to adapt to emerging healthcare trends.

The findings from this review emphasize a shift in focus within health professions education from assessing the preparedness of new graduates for the workplace to evaluating the readiness of the workplace to support these individuals. This shift emphasizes the crucial need to investigate the receptivity of clinical environments, strategies and support offered to new graduates. These factors encompass the development of personal characteristics, education-related elements, cognitive and psychological attributes, and supportive social factors. This review highlights challenges for HPE, suggesting the need for innovative and alternative educational approaches to develop R2P, including the potential of interprofessional education. Future studies should explore the relationships between R2P, stakeholders’ perceptions of the educational curriculum, and the quality of support in the professional practice environment.

## Conclusion

Readiness for professional practice stands as a foundation of healthcare education, ensuring that newly qualified health professionals are equipped to provide safe, competent, and compassionate care. By understanding the multifaceted nature of R2P, adopting a holistic approach to assessment and feedback, and implementing effective strategies for enhancing practice readiness, healthcare educators, employers and the health service can empower graduates to confidently enter the workforce.

## The ACHIEVE network

Academy for Collaborative Health Interprofessional Education and Vibrant Excellence, NSW, Australia.

## References

[1] Wolff AC Pesut B Regan S. New graduate nurse practice readiness: perspectives on the context shaping our understanding and expectations. Nurse Educ Today. (2010) 30:187–91. doi: 10.1016/j.nedt.2009.07.011 19699561

[2] Barr J Ogden KJ Rooney K Robertson I. Preparedness for practice: the perceptions of graduates of a regional clinical school. Med J Aust. (2017) 206:447–52. doi: 10.5694/mja16.00845, PMID: 28566071

[3] Harrison H Birks M Franklin RC Mills J. Fostering graduate nurse practice readiness in context. Collegian. (2020) 27:115–24. doi: 10.1016/j.colegn.2019.07.006, PMID: 31231947

[4] Malau-Aduli BS Jones K Alele F Adu MD Drovandi A Knott G. Readiness to enter the workforce: perceptions of health professions students at a regional Australian university. BMC Med Educ. (2022) 22:89. doi: 10.1186/s12909-022-03120-4, PMID: 35139831 PMC8827198

[5] Monrouxe LV Bullock A Gormley G Kaufhold K Kelly N Roberts CE. New graduate doctors’ preparedness for practice: a multistakeholder, multicentre narrative study. BMJ Open. (2018) 8:e023146. doi: 10.1136/bmjopen-2018-023146, PMID: 30158236 PMC6119440

[6] Oliver B Jorre de St Jorre T. Graduate attributes for 2020 and beyond: recommendations for Australian higher education providers. High Educ Res Dev. (2018) 37:821–36. doi: 10.1080/07294360.2018.1446415

[7] Aitken G Jones D Fawns T Sutherland D Henderson S. Using Bourdieu to explore graduate attributes in two online Master’s programmes. Adv Health Sci Educ. (2019) 24:559–76. doi: 10.1007/s10459-019-09885-6, PMID: 30915641 PMC6647485

[8] Laidlaw A Guild S Struthers J. Graduate attributes in the disciplines of medicine, dentistry and veterinary medicine: a survey of expert opinions. BMC Med Educ. (2009) 9:1–6. doi: 10.1186/1472-6920-9-28 19500358 PMC2701429

[9] Miller L Brushett S Ayn C Furlotte K Jackson L MacQuarrie M. Developing a competency framework for population health graduate students through student and faculty collaboration. Pedagogy Health Promot. (2021) 7:280–8. doi: 10.1177/2373379919859607, PMID: 39081902

[10] McGarity T Monahan L Acker K Pollock W. Nursing graduates’ preparedness for practice: substantiating the call for competency-evaluated nursing education. Behav Sci. (2023) 13:553. doi: 10.3390/bs13070553, PMID: 37504000 PMC10376128

[11] Vista AM Balucio TM Yap TB. Graduating student nurses’ preparedness for nursing practice amid COVID-19. Africa J Nurs Midwifery. (2022) 24:1–17. doi: 10.25159/2520-5293/12050

[12] Walker A Yong M Pang L Fullarton C Costa B Dunning AM. Work readiness of graduate health professionals. Nurse Educ Today. (2013) 33:116–22. doi: 10.1016/j.nedt.2012.01.007, PMID: 22336479

[13] Kamal KA Benson JE. Cultural competence in mental healthcare amongst central American migrants. Houston, Texas, USA: Elliott T. Bowers Honors College (2021).

[14] Chua BS Cosmas G Arsat N. Nurses’ preparedness, readiness, and anxiety in managing COVID-19 pandemic. Asia Pac J Public Health. (2021) 33:564–70. doi: 10.1177/10105395211012170, PMID: 33938289

[15] Muruvan C Downing C Kearns IJ. Preparedness for practice: experiences of newly qualified professional nurses in a private hospital setting. Int J Afr Nurs Sci. (2021) 15:100329. doi: 10.1016/j.ijans.2021.100329

[16] AlMekkawi M El Khalil R. Undergraduate nursing students' readiness to practice: views of the senior students in the United Arab Emirates. Nurse Educ. (2022) 47:E86–90. doi: 10.1097/NNE.0000000000001153, PMID: 35113063

[17] Sharma S Kalal N Rani R. Clinical practice readiness of nursing graduates. Clin Mother Child Health. (2021) 18:381. doi: 10.35248/2090-7214.21.18.381

[18] Padley J Boyd S Jones A Walters L. Transitioning from university to postgraduate medical training: a narrative review of work readiness of medical graduates. Health Sci Rep. (2021) 4:e270. doi: 10.1002/hsr2.270, PMID: 33855193 PMC8025846

[19] Morrow G Johnson N Burford B Rothwell C Spencer J Peile E. Preparedness for practice: the perceptions of medical graduates and clinical teams. Med Teach. (2012) 34:123–35. doi: 10.3109/0142159X.2012.643260, PMID: 22288990

[20] Shamim H. Importance of leadership skills for international medical graduates. J Int Med Graduates. (2023) 2:1–45. doi: 10.56570/jimgs.v2i1.57

[21] Morley CP Rosas SR Mishori R Jordan W Jarris YS Competencies Work Group FM. Essential public health competencies for medical students: establishing a consensus in family medicine. Teach Learn Med. (2017) 29:255–67. doi: 10.1080/10401334.2016.1268964, PMID: 28632011

[22] Kuzmenko N Ivanytska T Poda O Nesina I Tanianska S. Perception of readiness of future doctors for professional activities and determination of key factors affecting readiness to work. Bull Probl Biol Med. (2023) 1:246–53. doi: 10.29254/2077-4214-2023-1-168-246-253

[23] Chaou C-H Yu S-R Chang Y-C Ma S-D Tseng H-M Hsieh M-J. The evolution of medical students’ preparedness for clinical practice during the transition of graduation: a longitudinal study from the undergraduate to postgraduate periods. BMC Med Educ. (2021) 21:1–9. doi: 10.1186/s12909-021-02679-8 33957907 PMC8101179

[24] Sanson-Fisher R Hobden B Carey M Mackenzie L Hyde L Shepherd J. Interactional skills training in undergraduate medical education: ten principles for guiding future research. BMC Med Educ. (2019) 19:1–7. doi: 10.1186/s12909-019-1566-2 31092235 PMC6521390

[25] Fejzic J Barker M. ‘The readiness is all’–Australian pharmacists and pharmacy students concur with Shakespeare on work readiness. Pharm Educ. (2015) 15:76–82.

[26] Cutler S Morecroft C Carey P Kennedy T. Are pharmacy students adequately prepared to work in healthcare teams? Pharm Educ. (2020) 20:43–51.

[27] James PB Cole CP. Intern pharmacists' perceived preparedness for practice, their extent of involvement in pharmacy related activities and future career choices in Sierra Leone: a baseline descriptive survey. Pharm Educ. (2016) 16:26–32.

[28] O’Sullivan TA Sy E Bacci JL. Essential attributes for the community pharmacist as care provider. Am J Pharm Educ. (2020) 84:7125. doi: 10.5688/ajpe7125, PMID: 32292190 PMC7055410

[29] Wells C Olson R Bialocerkowski A Carroll S Chipchase L Reubenson A. Work readiness of new graduate physical therapists for private practice in Australia: academic faculty, employer, and graduate perspectives. Phys Ther. (2021) 101:1–12. doi: 10.1093/ptj/pzab078, PMID: 33686439

[30] Evans L Cheffins A. Simulation for preceptee physiotherapists readiness to practise. Int J Healthcare Simul. (2022) 2:A15–A16. doi: 10.54531/LZYR6785

[31] Dennett A Rowe A Mortimer J Gordon C Glagovski S Osadnik C. Perceptions and work-readiness of Australian physiotherapists in cancer care: a national evaluation. Physiotherapy. (2021) 113:1–7. doi: 10.1016/j.physio.2021.06.003, PMID: 34399131

[32] Chesterton P Chesterton J Alexanders J. New graduate physiotherapists’ perceived preparedness for clinical practice. A cross-sectional survey. Eur J Physiother. (2021) 25:33–42. doi: 10.1080/21679169.2021.1958007

[33] Martiáñez-Ramírez NL Pineda-Galán C Rodríguez-Bailón M Romero-Galisteo R-P. Competence assessment rubric in the physiotherapy practicum. PLoS One. (2022) 17:e0264120. doi: 10.1371/journal.pone.0264120, PMID: 35213586 PMC8880643

[34] Samant O Vardan GDV. Perception of undergraduate pyhsiotherapy students towards clinical attributes and clinical teaching. Int J Physiother Res. (2020) 8:3715–22. doi: 10.16965/ijpr.2020.185

[35] Ingram M Forbes R Jones A. Physiotherapy new graduate self-efficacy and readiness to engage in pain assessment and management: a mixed-methods study. Focus Health Prof Educ. (2019) 20:65–83. doi: 10.11157/fohpe.v20i3.362

[36] Oluwatosin L Ogundero AF. Career and work readiness of nutrition and dietetics trainees in Nigerian universities. World Nutr. (2021) 12:92–102. doi: 10.26596/wn.202112192-102

[37] Smythe JA Schumacher JR Cullen RW Ma YJ. Personal attributes of successful interns as perceived by dietetic internship directors and preceptors from varying generations. Open Nutr J. (2015) 9:28–34. doi: 10.2174/1876396001509010028

[38] Palermo C Capra S Ash S Beck E Truby H Jolly B. Professional competence standards, learning outcomes and assessment: Designing a valid strategy for nutrition and dietetics: Department of Education, skills and employment; (2014).

[39] Koemel NA Shafieizadeh K Farr BR. Career readiness in the dietetics curriculum. J Acad Nutr Diet. (2021) 121:15–24. doi: 10.1016/j.jand.2020.07.015, PMID: 32950459

[40] Jones D McAllister L Lyle D. Stepping out of the shadows: allied health student and academic perceptions of the impact of a service-learning experience on student's work-readiness and employability. J Teach Learn Graduate Employability. (2015) 6:66–87. doi: 10.21153/jtlge2015vol6no1art574

[41] Judd B Brentnall J Scanlan JN Thomson K Blackstock F Mandrusiak A. Evaluating allied health students’ readiness for placement learning. BMC Med Educ. (2023) 23:1–12. doi: 10.1186/s12909-023-04005-w 36709272 PMC9883866

[42] Lawton V Ilhan E Pacey V Jones TM Walker A Dean CM. The validation and refinement of a Work Readiness Scale for graduate Allied Health Professionals. Res Sq. (2022). doi: 10.21203/rs.3.rs-2348757/v1

[43] O'Brien M Troy K Kirkpatrick J. The allied health work readiness study: identifying personal characteristics signalling work readiness in allied health students. Int J Allied Health Sci Pract. (2020) 18:5. doi: 10.46743/1540-580X/2020.1859

[44] Le Huray L Murry AT Mughal H Crowshoe L. Readiness to practice in health care: an empirical definition based on a content analysis of the literature. J Contin Educ Nurs. (2023) 54:302–12. doi: 10.3928/00220124-20230620-03, PMID: 37390305

[45] Merga M. Gaps in work readiness of graduate health professionals and impact on early practice: possibilities for future interprofessional learning. Focus Health Prof Educ. (2016) 17:14–29. doi: 10.11157/fohpe.v17i3.174

[46] Page MJ McKenzie JE Bossuyt PM Boutron I Hoffmann TC Mulrow CD. The PRISMA 2020 statement: an updated guideline for reporting systematic reviews. BMJ. (2021) 372:n71. doi: 10.1136/bmj.n71 33782057 PMC8005924

[47] Covidence (Veritas Health Innovation). (n.d.) Covidence systematic review software. Melbourne, Australia. Available at: www.covidence.org (Accessed February, 2024).

[48] Harrison R Jones B Gardner P Lawton R. Quality assessment with diverse studies (QuADS): an appraisal tool for methodological and reporting quality in systematic reviews of mixed- or multi-method studies. BMC Health Serv Res. (2021) 21:1–20. doi: 10.1186/s12913-021-06122-y 33588842 PMC7885606

[49] Pullen D Ahchay D. A case study of new nurses’ transition from university to work. Teach Learn Nurs. (2022) 17:282–95. doi: 10.1016/j.teln.2022.04.004, PMID: 38959702

[50] Ersoy E Ayaz-Alkaya S. Academic self-efficacy, personal responsibility, and readiness for professional practice in nursing students: a descriptive and correlational design. Nurse Educ Today. (2024) 132:106007. doi: 10.1016/j.nedt.2023.106007, PMID: 37922765

[51] Willman A Bjuresäter K Nilsson J. Newly graduated registered nurses' self-assessed clinical competence and their need for further training. Nurs Open. (2020) 7:720–30. doi: 10.1002/nop2.443, PMID: 32257259 PMC7113520

[52] Zhang J Makanjee C Hayre CM Lewis S. Australian graduate radiographers' perspectives and experiences of work readiness. J Med Radiat Sci. (2023) 70:254–61. doi: 10.1002/jmrs.675, PMID: 37015838 PMC10500112

[53] Phan A Tan S Martin R Mandrusiak A Forbes R. Exploring new-graduate physiotherapists’ preparedness for, and experiences working within, Australian acute hospital settings. Physiother Theory Pract. (2023) 39:1918–28. doi: 10.1080/09593985.2022.2059424, PMID: 35387567

[54] Sheehan D de Bueger TM Thorogood J Sitters S Deo A. Beyond competencies–describing work ready plus graduates for the New Zealand medical imaging workforce. J Med Radiat Sci. (2018) 65:275–81. doi: 10.1002/jmrs.290, PMID: 29962009 PMC6275251

[55] Walters G Hoffart N Kring D Whitley T Horne L Almotairy M. Work readiness of newly licensed RNs. J Nurs Adm. (2022) 52:469–73. doi: 10.1097/NNA.0000000000001184, PMID: 35973193

[56] Attrill S McAllister S Brebner C. Not too little, not too much: supervisor perceptions of work-readiness of speech-language pathology graduates. Adv Health Sci Educ. (2022) 27:87–106. doi: 10.1007/s10459-021-10073-8, PMID: 34545503

[57] O'Brien K Moore A Hartley P Dawson D. Lessons about work readiness from final year paramedic students in an Australian university. Australas J Paramed. (2013) 10:1–3. doi: 10.33151/ajp.10.4.52

[58] Gruenberg K Hsia S O’Brien B O’Sullivan P. Exploring multiple perspectives on pharmacy students’ readiness for advanced pharmacy practice experiences. Am J Pharm Educ. (2021) 85:8358. doi: 10.5688/ajpe8358, PMID: 34283732 PMC8174613

[59] Lim SH Ang SY Aloweni F Siow KC Koh SB Ayre TC. Factors associated with practice readiness among newly qualified nurses in their first two years of practice. Nurse Educ Today. (2024) 136:106143. doi: 10.1016/j.nedt.2024.106143, PMID: 38422796

[60] Mak VS March G Clark A Gilbert AL. Australian intern pharmacists’ perceived preparedness for practice, and their expectations and experiences of the internship year and future career intentions. Integr Pharm Res Pract. (2013) 2:25–34. doi: 10.2147/IPRP.S50387

[61] Ottrey E Rees CE Kemp C Brock TP Leech M Lyons K. Exploring healthcare graduates' conceptualisations of preparedness for practice: a longitudinal qualitative research study. Med Educ. (2021) 55:1078–90. doi: 10.1111/medu.14475, PMID: 33617656

[62] Almarzoky Abuhussain SS Elrggal ME Salamatullah AK Althobaity AA Alotaibi AF Almeleebia TM. Work readiness scale for pharmacy interns and graduates: a cross-sectional study. Saudi Pharm J. (2021) 29:976–80. doi: 10.1016/j.jsps.2021.07.018, PMID: 34588843 PMC8463463

[63] Walker A Campbell K. Work readiness of graduate nurses and the impact on job satisfaction, work engagement and intention to remain. Nurse Educ Today. (2013) 33:1490–5. doi: 10.1016/j.nedt.2013.05.008, PMID: 23742716

[64] Almadani N Hables RM Alharbi J Alamri M Alshammari M. Nurse interns’ perception of clinical preparation and readiness for clinical internship experiences. J Nurs Manag. (2024) 2024:6682600. doi: 10.1155/2024/6682600 40224802 PMC11919187

[65] Jamieson I Sims D Basu A Pugh K. Readiness for practice: the views of New Zealand senior nursing students. Nurse Educ Pract. (2019) 38:27–33. doi: 10.1016/j.nepr.2019.05.007, PMID: 31174136

[66] Woods C West C Mills J Park T Southern J Usher K. Undergraduate student nurses’ self-reported preparedness for practice. Collegian. (2015) 22:359–68. doi: 10.1016/j.colegn.2014.05.003, PMID: 26775522

[67] Fenech R Baguant P Abdelwahed I. Work readiness across various specializations. AJIS. (2020) 9:86–92. doi: 10.36941/ajis-2020-0064, PMID: 39396967

[68] Musallam EA Flinders B. Senior BSN students’ confidence, comfort, and perception of readiness for clinical practice: the impacts of COVID-19. Int J Nurs Educ Scholarsh. (2021) 18:20200097. doi: 10.1515/ijnes-2020-0097 33882201

[69] Nweke CI Abazie OH Adetunji AJ Okwuikpo MI. Readiness for clinical practice amidst coronavirus among nursing students in Southwest Nigeria. Int J Afr Nurs Sci. (2021) 15:100328. doi: 10.1016/j.ijans.2021.100328, PMID: 34277348 PMC8276559

[70] Usher K Mills J West C Park T Woods C. Preregistration student nurses' self-reported preparedness for practice before and after the introduction of a capstone subject. J Clin Nurs. (2015) 24:3245–54. doi: 10.1111/jocn.12996 26374447

[71] Graham P Padley J Williams S Gonzalez-Chica D Isaac V Walters L. Australian rural medical students' perceived readiness for work as a junior doctor: a cross-sectional national survey. Aust J Rural Health. (2023) 31:999–1007. doi: 10.1111/ajr.13035, PMID: 37650537

[72] Lagali-Jirge V Umarani M. Evaluation of readiness to practice among interns at an Indian dental school. J Contemp Med Educ. (2014) 2:227–31. doi: 10.5455/jcme.20141105071940

[73] Anokwuru RA Daniels FM. Perceptions of baccalaureate graduates on their clinical nursing education and its effectiveness in their service delivery. Afr J Nurs Midwifery. (2021) 23:17. doi: 10.25159/2520-5293/8165

[74] Haruzivishe C Macherera DM. Perceived readiness to practice among BSC honors in nursing graduates: implications for training. Open Access Lib J. (2021) 8:1–12. doi: 10.4236/oalib.1107138

[75] Lanahan M Montalvo B Cohn T. The perception of preparedness in undergraduate nursing students during COVID-19. J Prof Nurs. (2022) 42:111–21. doi: 10.1016/j.profnurs.2022.06.002, PMID: 36150848 PMC9222345

[76] Casey K Loresto F Jr Lundy K Humphrey K Oja KJ. Impact of the pandemic on newly licensed nurses’ role transition experiences. J Nurses Prof Dev. (2024) 40:111–7. doi: 10.1097/NND.0000000000001038, PMID: 38411564

[77] Grant S Sheridan L Webb SA. Newly qualified social workers’ readiness for practice in Scotland. Br J Soc Work. (2017) 47:487–506. doi: 10.1093/bjsw/bcv146

[78] Meyer G Shatto B. Resilience and transition to practice in direct entry nursing graduates. Nurse Educ Pract. (2018) 28:276–9. doi: 10.1016/j.nepr.2017.10.008, PMID: 29042183

[79] Opoku EN Khuabi LJ van Niekerk L. Exploring the factors that affect the transition from student to health professional: an integrative review. BMC Med Educ. (2021) 21:558. doi: 10.1186/s12909-021-02978-0, PMID: 34727905 PMC8561904

[80] Bäck L Sharma B Karlström A Tunon K Hildingsson I. Professional confidence among Swedish final year midwifery students–a cross-sectional study. Sex Reprod Healthc. (2017) 14:69–78. doi: 10.1016/j.srhc.2017.10.003, PMID: 29195637

[81] Bradley L Barr JA Finn J. Work readiness of graduating nursing students: case study research. Teach Learn Nurs. (2023) 18:383–8. doi: 10.1016/j.teln.2023.03.012

[82] Hyun A Tower M Turner C. Exploration of the expected and achieved competency levels of new graduate nurses. J Nurs Manag. (2020) 28:1418–31. doi: 10.1111/jonm.13105, PMID: 32687641

[83] Shaw P Abbott M King TS. Preparation for practice in newly licensed registered nurses: a mixed-methods descriptive survey of preceptors. J Nurses Prof Dev. (2018) 34:325–31. doi: 10.1097/NND.0000000000000487, PMID: 30379766

[84] Thomas D Merrill K. Meeting theory-to-practice gaps? Evaluation of new graduates. J Nurses Prof Dev. (2023) 39:E168–73. doi: 10.1097/NND.0000000000000847, PMID: 37683222

[85] Powers K Montegrico J Pate K Pagel J. Nurse faculty perceptions of readiness for practice among new nurses graduating during the pandemic. J Prof Nurs. (2021) 37:1132–9. doi: 10.1016/j.profnurs.2021.09.003, PMID: 34887031 PMC8648075

[86] Rusch L Manz J Hercinger M Oertwich A McCafferty K. Nurse preceptor perceptions of nursing student progress toward readiness for practice. Nurse Educ. (2019) 44:34–7. doi: 10.1097/NNE.0000000000000546, PMID: 29794884

[87] Farris C Fowler M Wang S Wong E Ivy D. Descriptive survey of pharmacy students’ self-evaluation of advanced pharmacy practice experiences (APPE) and practice readiness using entrustable professional activities. Pharm Educ. (2023) 23:447–53. doi: 10.46542/pe.2023.231.447453

[88] Watt E Pascoe E. An exploration of graduate nurses' perceptions of their preparedness for practice after undertaking the final year of their bachelor of nursing degree in a university-based clinical school of nursing. Int J Nurs Pract. (2013) 19:23–30. doi: 10.1111/ijn.12032, PMID: 23432885

[89] Forbes R Ingram M. New-graduate physiotherapists’ readiness for practice and experiences of managing chronic pain; a qualitative study. Physiother Theory Pract. (2021) 37:1177–84. doi: 10.1080/09593985.2019.1692394, PMID: 31786970

[90] Grimm KL Barker S. A Pandemic's impact: newly licensed nurse self-efficacy following increased use of simulation. J Nurses Prof Dev. (2023) 39:E108–11. doi: 10.1097/NND.0000000000000848, PMID: 37683212

[91] Kasita REN Daniels ER Karera A. Preparedness to assume professional roles: experiences of recently qualified radiographers: a qualitative study. J Med Radiat Sci. (2023) 70:262–9. doi: 10.1002/jmrs.690, PMID: 37219064 PMC10500110

[92] Adam K Strong J Chipchase L. Readiness for work injury management and prevention: important attributes for early graduate occupational therapists and physiotherapists. Work (Reading, Mass). (2014) 48:567–78. doi: 10.3233/WOR-141912, PMID: 24962310

[93] Clark K Biesiekierski JR Farrer OD Stefoska-Needham A Beckett EL Lawlis T. Nutrition employability and graduate readiness: the Australian working in nutrition study. J Hum Nutr Diet. (2024) 37:685–94. doi: 10.1111/jhn.13295, PMID: 38446559

[94] Li J Huang Y Fong DY Chen J Song Y. Work readiness: its determinants and association with work-related outcomes among new graduate nurses. J Nurs Manag. (2022) 30:2968–81. doi: 10.1111/jonm.13691, PMID: 35596059

[95] Friedlander LT Wallace WD Broadbent JM Hanlin SM Lyons KM Cannon RD. Preparedness and competency of New Zealand graduates for general dental practice–perceptions from the workforce. Aust Dent J. (2024) 69:29–39. doi: 10.1111/adj.12981, PMID: 37740647

[96] Piccuito CM Santiago RR. New graduate respiratory therapists' perceptions of their transition to practice. Respir Care. (2023) 68:1365–76. doi: 10.4187/respcare.11004, PMID: 37185116 PMC10506651

[97] Duijn C Bok H Ten Cate O Kremer W. Qualified but not yet fully competent: perceptions of recent veterinary graduates on their day-one skills. Vet Rec. (2020) 186:216. doi: 10.1136/vr.105329, PMID: 31767696

[98] Illing JC Morrow GM Rothwell nee Kergon CR Burford BC Baldauf BK Davies CL. Perceptions of UK medical graduates’ preparedness for practice: a multi-Centre qualitative study reflecting the importance of learning on the job. BMC Med Educ. (2013) 13. doi: 10.1186/1472-6920-13-34 PMC3599362 23446055

[99] Hatzenbuhler NJ Klein JE. Educational preparation for clinical practice: reflections of newly graduated RNs. Nurse Educ. (2019) 44:93–7. doi: 10.1097/NNE.0000000000000550, PMID: 29794883

[100] Reynolds K McLean M. Clinical supervisors’ perceptions of podiatry students’ preparedness for clinical placement and graduates’ preparedness for podiatry practice in Australia: an exploratory study. Focus Health Prof Edu. (2021) 22:1–22. doi: 10.11157/fohpe.v22i2.339

[101] Akinkugbe AA Garcia DT Smith CS Brickhouse TH Mosavel M. A descriptive pilot study of the immediate impacts of COVID-19 on dental and dental hygiene students' readiness and wellness. J Dent Educ. (2021) 85:401–10. doi: 10.1002/jdd.12456, PMID: 33084054 PMC8043566

[102] Mustakallio S Näpänkangas R Narbutaite J Virtanen JI. Graduating dentists’ perceptions about their professional competence in Finland and Lithuania. Eur J Dent Educ. (2020) 24:227–32. doi: 10.1111/eje.12488, PMID: 31845488

[103] Woolley T Clithero-Eridon A Elsanousi S Othman AB. Does a socially-accountable curriculum transform health professional students into competent, work-ready graduates? A cross-sectional study of three medical schools across three countries. Med Teach. (2019) 41:1427–33. doi: 10.1080/0142159X.2019.1646417, PMID: 31407932

[104] Ford CR Astle KN Garza KB Kleppinger EL. Exploring standardized persons' expectations for practice-readiness among student pharmacists. Curr Pharm Teach Learn. (2021) 13:492–9. doi: 10.1016/j.cptl.2021.01.016, PMID: 33795100

[105] Stulz VM Elmir R Reilly H Francis L. A pilot study: transitioning into a new graduate midwife - perspectives about a unique student-led practice. Women Birth. (2023) 36:e369–77. doi: 10.1016/j.wombi.2022.09.008, PMID: 36175297

[106] Kinnane P Kennedy N Quinton A. Work readiness attributes: comparative views of clinical supervisors and final year sonography students. Sonography. (2021) 8:82–9. doi: 10.1002/sono.12274

[107] Almotairy M Nahari A Moafa H Alanazi AA. Work readiness of newly graduated nurses transitioning to practice in Saudi Arabia: a cross-sectional study. J Nurs Manag. (2022) 30:4523–32. doi: 10.1111/jonm.13893, PMID: 36326491

[108] Govindaraju V. Interpersonal communication skills in healthcare: literature review on doctors and patients communication. Multicult Educ. (2021) 7:324–332. doi: 10.5281/zenodo.5790211

[109] Kourkouta L Papathanasiou IV. Communication in nursing practice. Mater Socio-Med. (2014) 26:65. doi: 10.5455/msm.2014.26.65-67, PMID: 24757408 PMC3990376

[110] Moore PM Rivera S Bravo-Soto GA Olivares C Lawrie TA. Communication skills training for healthcare professionals working with people who have cancer. Cochrane Database Syst Rev. (2018) 7:Cd003751. doi: 10.1002/14651858 30039853 PMC6513291

[111] Hachoumi N Eddabbah M El Adib AR. Health sciences lifelong learning and professional development in the era of artificial intelligence. Int J Med Inform. (2023) 178:105171. doi: 10.1016/j.ijmedinf.2023.105171, PMID: 37573636

[112] Mlambo M Silén C McGrath C. Lifelong learning and nurses’ continuing professional development, a metasynthesis of the literature. BMC Nurs. (2021) 20:1–13. doi: 10.1186/s12912-021-00579-2 33853599 PMC8045269

[113] Qalehsari MQ Khaghanizadeh M Ebadi A. Lifelong learning strategies in nursing: a systematic review. Electron Physician. (2017) 9:5541. doi: 10.19082/5541, PMID: 29238496 PMC5718860

[114] Taylor RB. Staying up to date. Medical wisdom and doctoring: the art of 21st century practice. New York: Springer-Verlag. (2010):177–197.

[115] Frenk J Chen LC Chandran L Groff EOH King R Meleis A. Challenges and opportunities for educating health professionals after the COVID-19 pandemic. Lancet. (2022) 400:1539–56. doi: 10.1016/S0140-6736(22)02092-X, PMID: 36522209 PMC9612849

[116] Holt DT Helfrich CD Hall CG Weiner BJ. Are you ready? How health professionals can comprehensively conceptualize readiness for change. J Gen Intern Med. (2010) 25:50–5. doi: 10.1007/s11606-009-1112-8, PMID: 20077152 PMC2806967

[117] Howard B Diug B Ilic D. Methods of teaching evidence-based practice: a systematic review. BMC Med Educ. (2022) 22:742. doi: 10.1186/s12909-022-03812-x, PMID: 36289534 PMC9607697

[118] Lee T Damiran D Konlan KD Ji Y Yoon YS Ji H. Factors related to readiness for practice among undergraduate nursing students: a systematic review. Nurse Educ Pract. (2023) 69:103614. doi: 10.1016/j.nepr.2023.103614, PMID: 37002991

[119] Masso M Sim J Halcomb E Thompson C. Practice readiness of new graduate nurses and factors influencing practice readiness: a scoping review of reviews. Int J Nurs Stud. (2022) 129:104208. doi: 10.1016/j.ijnurstu.2022.104208, PMID: 35344839

[120] Mensah NK Adzakpah G Kissi J Boadu RO Lasim OU Oyenike MK. Health professional’s readiness and factors associated with telemedicine implementation and use in selected health facilities in Ghana. Heliyon. (2023) 9:e14501. doi: 10.1016/j.heliyon.2023.e14501, PMID: 36945351 PMC10022178

[121] Gardiner I Sheen J. Graduate nurse experiences of support: a review. Nurse Educ Today. (2016) 40:7–12. doi: 10.1016/j.nedt.2016.01.016, PMID: 27125143

[122] Labrague L McEnroe-Petitte D. Job stress in new nurses during the transition period: an integrative review. Int Nurs Rev. (2018) 65:491–504. doi: 10.1111/inr.12425, PMID: 29266201

[123] Almost J Wolff AC Stewart-Pyne A McCormick LG Strachan D D'Souza C. Managing and mitigating conflict in healthcare teams: an integrative review. J Adv Nurs. (2016) 72:1490–505. doi: 10.1111/jan.12903, PMID: 26822008

[124] Ramsay MA, editor. (2001). Conflict in the health care workplace. Baylor University Medical Centre Proceedings; Taylor & Francis. 10.1080/08998280.2001.11927749 PMC1291328 16369603

[125] Belinda R. Bridging the skill gap through internships: stakeholders theory perspective. Productivity. (2023) 63:457–64. doi: 10.32381/PROD.2023.63.04.8

[126] Parson L Childs B Elzie P. Using competency-based curriculum design to create a health professions education certificate program that meets the needs of students, administrators, faculty, and patients. Health Profes Educ. (2018) 4:207–17. doi: 10.1016/j.hpe.2018.03.008

[127] Javed MQ Nawabi S Bhatti UA Atique S AlAttas MH Abulhamael AM. How well prepared are dental students and new graduates in Pakistan—a cross-sectional national study. Int J Environ Res Public Health. (2023) 20:1506. doi: 10.3390/ijerph20021506, PMID: 36674261 PMC9859325

[128] Smith SM Buckner M Jessee MA Robbins V Horst T Ivory CH. Impact of COVID-19 on new graduate nurses' transition to practice: loss or gain? Nurse Educ. (2021) 46:209–14. doi: 10.1097/NNE.0000000000001042, PMID: 33988534

[129] Al-Rawajfah OM AlBashayreh A Al Sabei SD Al-Maqbali M Al YA. Role transition from education to practice and its impact on the career futures of Omani nurses. Nurse Educ Pract. (2023) 68:103594. doi: 10.1016/j.nepr.2023.103594, PMID: 36889168

[130] Atkinson R McElroy T. Preparedness for physiotherapy in private practice: novices identify key factors in an interpretive description study. Man Ther. (2016) 22:116–21. doi: 10.1016/j.math.2015.10.016, PMID: 26640225

[131] Carter K Stoehr JD. Preparedness for clinical practice and the development of professional competencies. J Physician Assist Educ. (2019) 30:164–7. doi: 10.1097/JPA.0000000000000262, PMID: 31385908

[132] Dlamini CP Mtshali NG Dlamini CH Mahanya S Shabangu T Tsabedze Z. New graduates' readiness for practice in Swaziland: an exploration of stakeholders' perspectives. J Nurs Educ Pract. (2014) 4:148. doi: 10.5430/jnep.v4n5p148

[133] Dudley M Khaw D Botti M Hutchinson AF. The relationship between the undergraduate clinical learning environment and work readiness in new graduate nurses: a pre-post survey study. Nurse Educ Today. (2020) 94:104587. doi: 10.1016/j.nedt.2020.104587, PMID: 32927394

[134] Harvey P Nightingale C Kippen R. Rural medical students' self-reported perceptions of preparedness to practice in the aboriginal and Torres Strait islander health context. Aust J Rural Health. (2021) 29:261–6. doi: 10.1111/ajr.12721, PMID: 33793025

[135] Lazarus G Findyartini A Putera AM Gamalliel N Nugraha D Adli I. Willingness to volunteer and readiness to practice of undergraduate medical students during the COVID-19 pandemic: a cross-sectional survey in Indonesia. BMC Med Educ. (2021) 21:138. doi: 10.1186/s12909-021-02576-0, PMID: 33648516 PMC7919987

[136] Leufer T Cleary-Holdforth J. Senior nursing students' perceptions of their readiness for practice prior to final year internship: part 2—a qualitative perspective. Dimens Crit Care Nurs. (2020) 39:81–90. doi: 10.1097/DCC.0000000000000407, PMID: 32000239

[137] Mariño R Delany C Manton D Reid K Satur J Crombie F. Preparedness for practice of newly qualified dental professionals in Australia-educator, employer, and consumer perspectives. BMC Med Educ. (2022) 22:396. doi: 10.1186/s12909-022-03476-7, PMID: 35606758 PMC9125536

[138] Missen K McKenna L Beauchamp A. Work readiness of nursing graduates: current perspectives of graduate nurse program coordinators. Contemp Nurse. (2015) 51:27–38. doi: 10.1080/10376178.2015.1095054 26394245

[139] Nelson C Mandrusiak A Forbes R. Perceived preparedness and training needs of new graduate physiotherapists’ working with first nations Australians. Physiother Theory Pract. (2024, 2024) 40:1–14. doi: 10.1080/09593985.2023.2179382 36809246

[140] Phillips KE Dzurec L Burgess A Beauvais A McNutt-Clarke B. Ramifications of the COVID-19 pandemic on nursing students’ transition to practice. J Nurses Prof Dev. (2023) 39:E196–201. doi: 10.1097/NND.0000000000000904, PMID: 37902641

[141] Sterner A Eklund A Nilsson MS. Prepared to learn but unprepared for work: a cross sectional survey study exploring the preparedness, challenges, and needs of newly graduated nurses entering a hospital-based transition program. Nurse Educ Pract. (2023) 72:103782. doi: 10.1016/j.nepr.2023.103782, PMID: 37717407

[142] Stoikov S Maxwell L Butler J Shardlow K Gooding M Kuys S. The transition from physiotherapy student to new graduate: are they prepared? Physiother Theory Pract. (2022) 38:101–11. doi: 10.1080/09593985.2020.1744206, PMID: 32212986

[143] Tarhan M Doğan P Kürklü A. The relationship between nurse–nurse collaboration and work readiness among new graduate nurses. Nurs Forum. (2022) 57:1104–10. doi: 10.1111/nuf.12795, PMID: 36036181

[144] Waite NM McCarthy L Milne E Hillier C Houle SK Dolovich L. Perceived preparedness for full-scope pharmacist services among recent doctor of pharmacy graduates from Ontario schools of pharmacy. J Am Pharm Assoc. (2018) 58:630–7. doi: 10.1016/j.japh.2018.06.016, PMID: 30077565

[145] Wijnen-Meijer M Ten Cate O van der Schaaf M Burgers C Borleffs J Harendza S. Vertically integrated medical education and the readiness for practice of graduates. BMC Med Educ. (2015) 15:1–9. doi: 10.1186/s12909-015-0514-z 26689282 PMC4687104

[146] Wong WJ Lee RF Chong LY Lee SW Lau WM. Work readiness of pharmacy graduates: an exploratory study. Explor Res Clin Soc Pharm. (2024) 13:100389. doi: 10.1016/j.rcsop.2023.100389, PMID: 38204886 PMC10776422

[147] Alsalamah YS Alsalamah TS Saad Albagawi B Alslamah T El Tassi A Fawaz M. The relationship between work readiness and perceived clinical competence among graduates transitioning into professional practice. Int J Afr Nurs Sci. (2023) 18:100555. doi: 10.1016/j.ijans.2023.100555

[148] Syed Aznal SS Nadarajah VDV Kwa SK Seow LL Chong DW Molugulu N. Validation of a ‘work readiness scale’ for health professional (HP) graduates. Med Teach. (2021) 43:S33–8. doi: 10.1080/0142159X.2019.1697434, PMID: 31854254

[149] Thomas J Jinks A Jack B. Finessing incivility: the professional socialisation experiences of student nurses' first clinical placement, a grounded theory. Nurse Educ Today. (2015) 35:e4–9. doi: 10.1016/j.nedt.2015.08.022, PMID: 26358630

