# Correction: Readiness for professional practice among health professions education graduates: a systematic review

**DOI:** 10.3389/fmed.2026.1936811

**Published:** 2026-08-06

**Authors:** Katie Wynne, Felista Mwangi, Oyepeju Onifade, Omotola Abimbola, Fiona Jones, Julie Burrows, Marita Lynagh, Tazeen Majeed, Dileep Sharma, Elizabeth Bembridge, Michelle Stubbs, Carla Sunner, Jessica Bergmann, Tanmay Bagade, Bunmi S. Malau-Aduli

**Affiliations:** 1 School of Medicine and Public Health, University of Newcastle, Callaghan, NSW, Australia; 2 School of Nursing and Midwifery, University of Newcastle, Callaghan, NSW, Australia; 3 University Library, University of Newcastle, Callaghan, NSW, Australia; 4 Department of Rural Health, University of Newcastle, Tamworth, NSW, Australia; 5 School of Health Sciences, University of Newcastle, Callaghan, NSW, Australia

**Keywords:** readiness to practice, work-ready, professional practice, healthcare training, health graduates

The article type for this article was erroneously given as: Review. The correct type for the article is Systematic Review.

The original version of this article has been updated.

There was a mistake in Figure 1 as published. The PRISMA flowchart (Figure 1) lists the database sources as Google Scholar (*n* = 25), PubMed (*n* = 16), and “Unspecified (*n* = 4,122). The corrected Figure 1 appears below.

**Figure 1:**
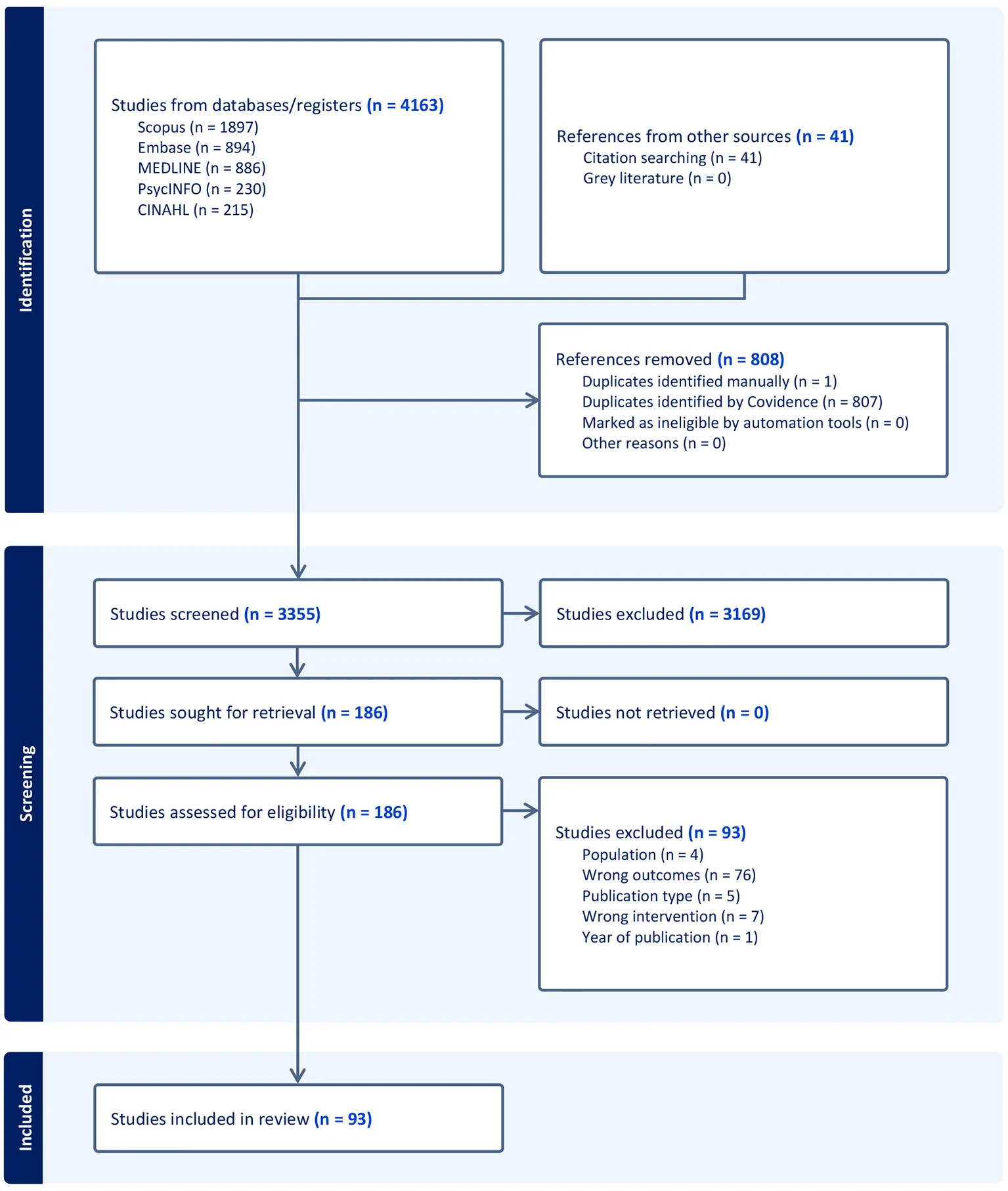
PRISMA chart.

The search details were not provided in the article as published. The full search strings, dates, and platforms are provided below.

**Table:** 

Database (Platform)	Search strings and controlled vocabulary terms	Result counts
Scopus	(“Health Occupations” OR “Students, Health Occupations” OR Medicine OR Nursing OR Midwifery OR Pharmacy OR Dentistry OR “Allied Health Occupations” OR “Occupational Therapy” OR “Physical Therapy Modalities” OR “Speech-Language Pathology” OR Audiology OR Dietetics OR “Nutrition Sciences” OR Optometry OR Podiatry OR “Medical Laboratory Science” OR Radiology OR “Physician Assistants” OR “Public Health” OR “Veterinary Medicine” OR Chiropractic) W/4 graduat^*^ (“ready for practi^*^” OR “readiness for practi^*^” OR “readiness to practi^*^” OR “practice readiness” OR “prepared for practi^*^” OR “prepared to practi^*^” OR “preparedness for practi^*^” OR “prepared for clinical practice” OR “preparedness for clinical practice” OR “transition to practice” OR “transition into practice” OR ((readiness OR preparedness OR prepar^*^ OR preparation) W/3 (practice OR work OR profession^*^))) #1 AND #2 “limit 3 to” (“yr=”2014 - Current“)	1,897
CINAHL (EBSCO)	((MH “Health Occupations+”) OR (MH “Students, Health Occupations+”) OR (MH Medicine+) OR (MH Nursing+) OR (MH Midwifery+) OR (MH Pharmacy+) OR (MH Dentistry+) OR (MH “Allied Health Occupations+”) OR (MH “Occupational Therapy+”) OR (MH “Physical Therapy Modalities+”) OR (MH “Speech-Language Pathology+”) OR (MH Audiology+) OR (MH Dietetics+) OR (MH “Nutrition Sciences+”) OR (MH Optometry+) OR (MH Podiatry+) OR (MH “Medical Laboratory Science+”) OR (MH Radiology+) OR (MH “Physician Assistants+”) OR (MH “Public Health+”) OR (MH “Veterinary Medicine+”) OR (MH Chiropractic+)) N4 graduat^*^ ((TI “ready for practi^*^” OR AB “ready for practi^*^”) OR (TI “readiness for practi^*^” OR AB “readiness for practi^*^”) OR (TI “readiness to practi^*^” OR AB “readiness to practi^*^”) OR (TI “practice readiness” OR AB “practice readiness”) OR (TI “prepared for practi^*^” OR AB “prepared for practi^*^”) OR (TI “prepared to practi^*^” OR AB “prepared to practi^*^”) OR (TI “preparedness for practi^*^” OR AB “preparedness for practi^*^”) OR (TI “prepared for clinical practice” OR AB “prepared for clinical practice”) OR (TI “preparedness for clinical practice” OR AB “preparedness for clinical practice”) OR (TI “transition to practice” OR AB “transition to practice”) OR (TI “transition into practice” OR AB “transition into practice”) OR (((TI readiness OR AB readiness) OR (TI preparedness OR AB preparedness) OR (TI prepar^*^ OR AB prepar^*^) OR (TI preparation OR AB preparation)) N3 ((TI practice OR AB practice) OR (TI work OR AB work) OR (TI profession^*^ OR AB profession^*^)))) S1 AND S2 “limit 3 to” (“yr=”2014 - Current”)	215
PsycInfo (Ovid)	(exp Health Occupations/or exp Students, Health Occupations/or exp Medicine/or exp Nursing/or exp Midwifery/or exp Pharmacy/or exp Dentistry/or exp Allied Health Occupations/or exp Occupational Therapy/or exp Physical Therapy Modalities/or exp Speech-Language Pathology/or exp Audiology/or exp Dietetics/or exp Nutrition Sciences/or exp Optometry/or exp Podiatry/or exp Medical Laboratory Science/or exp Radiology/or exp Physician Assistants/or exp Public Health/or exp Veterinary Medicine/or exp Chiropractic/) adj4 graduat^*^.mp. (“ready for practi^*^” or “readiness for practi^*^” or “readiness to practi^*^” or “practice readiness” or “prepared for practi^*^” or “prepared to practi^*^” or “preparedness for practi^*^” or “prepared for clinical practice” or “preparedness for clinical practice” or “transition to practice” or “transition into practice” or ((readiness or preparedness or prepar^*^ or preparation) adj3 (practice or work or profession^*^))).ti,ab. 1 and 2 limit 3 to (yr=“2014 - Current”)	230
EMBASE (Ovid)		894
Medline (Ovid)		886

The original version of this article has been updated.

