# Supplementary material for: Readiness for professional practice among health professions education graduates: a systematic review
Source: Front Med (Lausanne). 2024 Nov 8;11:1472834. doi: 10.3389/fmed.2024.1472834 (PMC11583157; doi:10.3389/fmed.2024.1472834)
Supplement: Supplementary file 1 [file Table_1.docx]

**Supplementary Table 1. Characteristics of the reviewed articles**

| **Title** | **Study objectives** | **Study design** | **Study Site** | **Population characteristics** | **Main findings** |
| --- | --- | --- | --- | --- | --- |
| Abuhussain, S.S.A., Elrggal, M.E., Salamatullah, A.K., Althobaity, A.A., Alotaibi, A.F., Almeleebia, T.M., Almangour, T.A. & Alhifany, A.A. Work readiness scale for pharmacy interns and graduates: A crosssectional study. (2021) | To assess the work readiness level of pharmacy students and graduates in Saudi Arabia, and identify factors associated with higher or lower work readiness | Quantitative | Saudi Arabia | 617 pharmacy students and graduates; 49.4% female | The students and graduates reported high levels of work readiness. Graduates from PharmD programs with additional training in areas like pharmaceutical marketing tended to demonstrate higher levels of readiness compared to those with BPharm degrees. |
| Adam, K., Strong, J. & Chipchase, L. Readiness for work injury management and prevention: Important attributes for early graduate occupational therapists and physiotherapists. (2014) | To develop a detailed qualitative account of the perceptions of employers and early graduates on the attributes required of early graduates in work injury management and prevention, and processes for effective transition to practice in this field. | Qualitative | Australia | 12 employers and 12 occupational therapists and physiotherapists | Employers and early graduates shared similar views that clinical education in work injury management and prevention was useful to early graduates entering this field. However, work injury management and prevention skills were not expected of early graduates by employers. Physiotherapy employers considered PT early graduates not yet ready for work injury management and prevention. |
| Akinkugbe A, Garcia D, Smith C, Brickhouse T, & Mosavel M. A descriptive pilot study of the immediate impacts of COVID-19 on dental and dental hygiene students' readiness and wellness. (2021) | To assess the immediate impacts of COVID-19 on dental and dental hygiene students' readiness to enter clinical practice or residency, and to assess the association between students' readiness and their well-being (anxiety, perceived stress, coping, social support, and resilience) | Quantitative | United States | 252 dental and dental hygiene students; 38% male and 62% female | Dental and dental hygiene students, particularly those in clinical years nearing graduation, experienced heightened anxiety due to the COVID-19 pandemic. There were no significant differences in coping abilities, as measured by social support, coping, and resilience, across different years in the dental program. However, certain demographic groups, including females, racial minorities, and those with altered graduation plans, exhibited significant differences in wellness measures and may require targeted interventions. |
| Almadani, N., Hables, R.M., Alharbi, J., Alamri, M. & Alshammari, M. Nurse interns' perception of clinical preparation and readiness for clinical internship experiences. (2024) | To assess nurse interns' perception of clinical preparation and readiness for clinical internship experiences, and examine the relationship between perceived clinical preparation and perceived readiness for nursing practice during the internship | Quantitative | Saudi Arabia | 130 female nurse interns; 26.1±4 years | A significant portion of nurse interns exhibited moderate (50%) or low levels (28.5%) of clinical preparation, while more than half of the interns (53.8%) were rated as moderately ready for practice, and over one-fifth (22.3%) were classified as having a low readiness level. The study identified a strong positive correlation between perceived clinical preparation and readiness for practice among nurse interns. |
| AlMekkawi, M., & El Khalil, R. Undergraduate nursing students' readiness to practice: Views of the senior students in the United Arab Emirates. (2022) | To investigate senior nursing students' perceptions of readiness to practice, and examine how senior students in the UAE view their readiness to practice safely and independently and perform registered nurses' job-specific requirements upon graduation | Mixed methods | United Arab Emirates | 117 female senior nursing students; 23.2±1.95 years | Generally, students felt well-prepared for their professional roles. They exhibited confidence in communication, problem-solving, and identifying patient safety risks. However, they were not ready in handling tasks like catheter insertion, blood draws, and central line care independently. |
| Almotairy, M., Nahari, A., Moafa, H. & Alanazi, A.A. Work readiness of newly graduated nurses transitioning to practice in Saudi Arabia: A cross-sectional study. (2022) | To explore work readiness among newly graduated nurses transitioning to practice in Saudi Arabia, and investigate the relationships between work readiness and the demographic characteristics of newly graduated nurses | Quantitative | Saudi Arabia | 248 newly graduated nurses; 44% male, 26.2% female and 29.8% unknown; 23.9±3.3 years | Generally, the newly graduated nurses demonstrated high levels of work readiness. Weekly working hours, the country and university of graduation, holding a second job, whether the hospital was their first-choice preference for employment, and whether nursing was their primary choice of study were factors influencing the graduates’ work readiness. |
| Al-Rawajfah, O. M., AlBashayreh, A., Al Sabei, S. D., Al-Maqbali, M., & Al Yahyaei, A. Role transition from education to practice and its impact on the career futures of Omani nurses. (2023) | To investigate newly graduated Omani nurses' perception of their role transition, and examine the predictors of a successful transition to professional nursing practice | Quantitative | Oman | 405 newly graduated nurses; 20-50 years; 20.2% male and 79.8% female | Appropriate national-level interventions are crucial to facilitate the smooth transition of nursing graduates into their professional roles. Recommendations included shortening the waiting time before employment and enhancing the quality of internship experiences and residency programs. |
| Anokwuru, R.A. & Daniels, F.M. Perceptions of Baccalaureate Graduates on their clinical nursing education and its effectiveness in their service delivery. (2021) | To explore the perceptions of baccalaureate nursing graduates on the effectiveness of their clinical education in their service delivery | Qualitative | Nigeria | 29 baccalaureate nursing graduates; 25-40 years; 59% male and 41% female | Graduates generally viewed their clinical education as adequate, despite facing challenges such as limited clinical time and negative attitudes from professional nurses. They appreciated the strong theoretical component of their curriculum in enhancing their clinical judgment and care delivery. |
| Atkinson, R. & McElroy, T. Preparedness for physiotherapy in private practice: Novices identify key factors in an interpretive description study. (2016) | To examine perceptions of preparedness in clinical and non-clinical areas of practice and identify supports influencing novice physiotherapist preparedness for work in private practice. | Qualitative | Australia | 8 novice physiotherapists; 4 male and 4 female; 23-25 years old | Graduate preparedness for work in private practice was significantly influenced by non-curricular experiences, elective curricular experiences, and the development of skills specific to private practice. Supportive colleagues in the private practice setting played a crucial role in enhancing new graduates' feelings of preparedness and facilitating their transition from student to practitioner. However, most participants lacked the skill of making appropriate referrals for radiological imaging upon entering private practice. |
| Attrill, S.L., McAllister, S. & Brebner, C. Not too little, not too much: supervisor perceptions of work-readiness of speech-language pathology graduates. (2021) | To explore the perceptions of supervisors from diverse work settings about work-readiness of speech-language pathology (SLP) graduates, and identify how supervisors' perceptions of work-readiness relate to graduates' perceptions described in previous literature | Qualitative | Australia and Singapore | 30 supervisor speech-language pathologists from 20 different workplaces | Supervisors in speech-language pathology identified four essential skills and attributes crucial for ensuring graduates' readiness for professional practice: independence, positive attitude, continuous learning ability, and effective teamwork. They emphasized the necessity for graduates to balance these attributes, considering the constraints of their university-based education and the practical demands of the workplace environment. |
| Bäck, L., Sharma, B., Karlström, A., Tunon, K. & Hildingsson, I. Professional confidence among Swedish final year midwifery students -A cross-sectional study. (2017) | To explore final year midwifery students' professional confidence in basic midwifery skills according to International Confederation of Midwives (ICM) competencies, and investigate factors associated with the students' confidence levels | Mixed methods | Sweden | 238 final-year midwifery students: 32±6.6 years | Students demonstrated confidence in managing normal pregnancy, labor, and birth. Students enrolled in midwifery programs affiliated with medical faculties showed higher confidence levels in handling obstetric emergencies compared to their peers in other programs. Background variables such as age and work experience were found to be associated with higher levels of confidence in specific skills among the students. |
| Barr, J., Ogden, K.J., Rooney, K. & Robertson, I. Preparedness for practice: the perceptions of graduates of a regional clinical school. (2017) | To assess medical graduates' self-reported preparedness for a range of clinical practice capabilities, including those related to patient-centered care | Quantitative | Australia | 135 medical students; 51% male and 49% female | Most graduates felt well or extremely well prepared in the majority of practice areas, with 80% feeling confident in 17 areas. However, there were differences in retrospective perceptions of preparedness between male graduates with 5-10 years of experience and more recent male graduates, while no such differences were noted for female graduates. Graduates felt least prepared in providing nutritional care, using audits, clinical governance, informatics, responding to errors/safety, and cultural competency, and most prepared in understanding patient-centered practice. |
| Bradley, L., Barr, J. A., & Finn, J. Work readiness of graduating nursing students: Case study research. (2023) | To explore how final year nursing students in Australia perceive themselves as work ready for registered nurse (RN) roles and clinical practice | Mixed-methods | Australia | 91 nursing students; 17% male and 83% female; majority (66%) were 19-28 years old | There was a wide variation of students perceived work readiness from confidence and readiness to feelings of being unprepared or unnoticed. The study highlighted concerns about the consistency and objectivity of clinical performance evaluations, noting variability in the use of assessment tools like Australian Nursing Standards Assessment Tool (ANSAT) and subjectivity in evaluations. More than half of the students were not satisfied with their clinical placements, feeling they were insufficiently prepared for their transition into the graduate year |
| Carter, K. & Stoehr, J.D. Preparedness for clinical practice and the development of professional competencies. (2019) | To assess students' perceived level of preparedness for specific clinical competencies just prior to graduation and identify the effectiveness of specific curricular methods on the development of professional competencies. | Quantitative | United States | 71 physician assistant students | Students felt most prepared for group dynamics, teamwork, and continuous professional development, but least prepared for pharmacotherapeutics and handling medical emergencies. Case groups were rated as the most effective teaching method for developing interpersonal and communication skills, while didactic lecturing was deemed the least effective. Didactic lecturing was considered the most effective for acquiring medical knowledge, though no single teaching method was identified as the best for integrating knowledge and fostering critical thinking. |
| Casey K, Loresto F, Lundy K, Humphrey K, & Oja KJ. Impact of the pandemic on newly licensed nurses' role transition experiences. (2024) | To examine differences in newly licensed nurses' role transition experiences while enrolled in a nurse residency program before and during the pandemic, and to compare baseline, 6-month, and 12-month program outcomes using the Casey-Fink Graduate Nurse Experience Survey between the pre-pandemic and pandemic cohorts. | Quantitative | United States | 454 newly licensed nurses; 20% male and 60% female; 30±6.6 years | NLNs participating in both pre-pandemic and pandemic cohorts demonstrated significant increases in role confidence over the 12-month nurse residency program. Despite fluctuations in professional satisfaction, with a decline at 6 months followed by a return to higher levels at 12 months, NLNs maintained high overall satisfaction. |
| Chesterton, P., Chesterton, J. & Alexanders, J. New graduate physiotherapists' perceived preparedness for clinical practice. A cross-sectional survey. (2023) | To explore new UK graduate physiotherapists' perceived preparedness for clinical practice | Mixed methods | United Kingdom | 365 newly qualified UK physiotherapists; 28 ± 8 years; 37% male, 62% female and 1% self-identifying other | Newly qualified physiotherapists in the UK generally reported feeling well-prepared by their pre-registration courses against the Health and Care Professions Council (HCPC) standards of proficiency, although they identified a gap in awareness of culture, equality, and diversity. They felt underprepared in exercise therapy, psychology, pain management, and manual therapy. Practical placements and seminars were highlighted as the most effective methods for preparing graduates for clinical practice, whereas online learning was perceived as less favorable. |
| Clark, K., Biesiekierski, J.R., Farrer, O.D., Stefoska‐Needham, A., Beckett, E.L., Lawlis, T., Mantzioris, E. & Swanepoel, L. Nutrition employability and graduate readiness: The Australian Working in Nutrition study. (2024) | To examine the current employment landscape for nutrition science graduates in Australia | Mixed-methods | Australia | 119 nutrition graduates; 7% male and 93% female; mainly 25-44 years old (84%) | The majority of nutrition graduates reported that they have found employment in fields related to food, nutrition, or health, with more than half currently working in these areas. Engagement in work-integrated learning (WIL) during their degree was noted to influence their career trajectories, often leading them to roles they had not initially anticipated. However, graduates reported inadequate research skills and knowledge in specialized areas such as the food industry, sports nutrition, and global/public health nutrition. |
| Dlamini, C.P., Mtshali, N.G., Dlamini, C.H., Mahanya, S., Shabangu, T. & Tsabedze, Z. New graduates' readiness for practice in Swaziland: An exploration of stakeholders' perspectives. (2014) | To explore stakeholders' perceptions of the clinical proficiency of new nursing graduates, and identify factors that could have contributed to the clinical proficiency of new nursing graduates | Qualitative | Swaziland | 31 registered nurses | Stakeholders in Swaziland widely perceived that most new nursing graduates were inadequately prepared for professional practice upon graduation. They attributed this to deficiencies in their educational preparation and lack of structured support and induction programs upon entering the workforce. The study recommended collaborative efforts among academia, healthcare service providers, and regulatory bodies to establish good quality assurance mechanisms in clinical education and to implement structured support systems for new nursing graduates. |
| Dudley, M., Khaw, D., Botti, M. & Hutchinson, A.F The relationship between the undergraduate clinical learning environment and work readiness. (2019) | To explore the relationship between the undergraduate clinical learning environment and nurse perceptions of work readiness prior to and following commencement as a new graduate nurse, describe the trajectory of perceived work readiness and evaluate the quality of the clinical learning environment and perceived work readiness associated with the Fellowship Model and other undergraduate education programs | Quantitative | Australia | 75 nursing students; 22 years (IQR=4); 4% male and 96% female | The study showed that the quality of the undergraduate clinical learning environment strongly correlates with the self-reported work readiness of nursing graduates. Key elements such as a student-centered approach, individualized learning experiences, appreciation for nurses' contributions, and an environment fostering innovation and adaptability were identified as significant contributors to graduates' perceived readiness for work. |
| Duijn, C., Bok, H., Ten Cate, O. & Kremer, W. Qualified but not yet fully competent: perceptions of recent veterinary graduates on their day-one skills. (2020) | To provide insight into recently graduated veterinarians' perceptions of their readiness for independent practice in the first year after graduation | Quantitative | Netherlands | 146 veterinarians; 27.5±2.1 years; 17% male and 83% female | Recently graduated veterinarians typically require nearly a year to feel prepared to independently perform critical professional tasks, with varying levels of supervision needed initially depending on the task. Most respondents rated the supervision they received early in their careers as sufficient or excellent. The study underscores the significance of thorough preparation during veterinary education and supportive guidance during the first year post-graduation to facilitate a smooth transition into clinical practice. |
| Ersoy E, & Ayaz-Alkaya S. Academic self-efficacy, personal responsibility, and readiness for professional practice in nursing students: A descriptive and correlational design. (2024) | To determine the levels of readiness for professional practice, academic self-efficacy, and personal responsibility among nursing students, and examine whether nursing students' sociodemographic characteristics affect their levels of personal responsibility, academic self-efficacy, and readiness for professional practice | Quantitative | Turkey | 832 nursing students; 20.57 ± 1.72 years; 14% male and 86% female | Nursing students exhibited a moderate level of readiness for professional practice, above-average academic self-efficacy, and good personal responsibility. Factors such as gender, academic level, practical experiences, satisfaction with nursing, and intention to work as a nurse influenced their academic self-efficacy, personal responsibility, and readiness for professional practice. The study recommends developing and implementing supportive educational programs to further enhance nursing students' readiness for professional practice and academic self-efficacy. |
| Farris C, Fowler F, Wang, Wong E, & Ivy D. Descriptive survey of pharmacy students' self-evaluation of Advanced Pharmacy Practice Experiences (APPE) and practice readiness using entrustable professional activities. (2023) | To evaluate pharmacy students' self-identified levels of entrustability before and after their advanced pharmacy practice experiences (APPE), and examine the degree of change in entrustability levels over the APPE year | Quantitative | United States | 249 third- and 106 fourth-year pharmacy students. | The study showed that APPE rotations significantly enhanced students' self-reported levels of entrustability across all 15 Entrustable Professional Activities domains. The domains with the lowest reported entrustability included overseeing pharmacy operations, optimizing medication use, and developing patient-centered goals and plans. By the end of their APPE year, students reported feeling sufficiently competent (a level of 4, indicating capability to practice unsupervised with distant preceptor availability) in all domains except overseeing pharmacy operations. |
| Fejzic, J., & Barker, M. 'The readiness is all' -Australian pharmacists and pharmacy students concur with Shakespeare on work readiness. (2015) | To explore the understanding and perceptions of work readiness among pharmacy students and their preceptors in order to inform future teaching strategies, particularly in the area of pharmacy practice and work-integrated learning (WIL). | Qualitative | Australia | 92 pharmacists and 71 pharmacy students | Both students and preceptors emphasized the importance of "soft", transferrable skills and social intelligence in addition to clinical and practice knowledge. Preceptors noted an increased competition for pharmacy intern positions, allowing them to be more selective and expect higher levels of skills and experience from applicants, including the ability to implement new professional pharmacy services to increase revenue and improve customer service. |
| Fenech, R., Baguant, P., & Abdelwahed, I. Work readiness across various specializations. (2020) | To study the work readiness of Emirati graduating students across various specializations | Quantitative | United Arab Emirates | 30 health professions students; mean age of 22 years | The concept of work readiness varied significantly across different academic specializations, showing strong differences in preparedness levels. It is recognized as a multidimensional construct influenced by factors such as specific skills, knowledge areas, and practical experience. |
| Forbes, R. & Ingram, M. New-graduate physiotherapists' readiness for practice and experiences of managing chronic pain; a qualitative study. (2021) | To understand new graduate physiotherapists' readiness for practice and experiences in managing chronic pain, as well as their perceptions of their training and support in this area | Qualitative | Australia | 15 new-graduate physiotherapists; 23±1.7 years; 4 male and 11 female | New graduate physiotherapists acknowledged the complex and personalized nature of chronic pain management and reported their entry-level training did not sufficiently address this complex area. They reported a need for additional practical experiences such as learning to negotiate care with patients resistant to treatment, to better equip them for managing chronic pain in clinical settings. Mentorship and collaboration within the interprofessional team was identified as essential workplace support. |
| Ford CR, Astle KN, Garza KB, & Kleppinger EL. Exploring standardized persons' expectations for practice-readiness among student pharmacists. (2021) | To explore the qualities and behaviors that standardized persons (SPs) associate with each of the three practice-readiness rating levels for student pharmacists in an OSCE setting to better understand how SPs evaluate student pharmacists for readiness to enter practice | Qualitative | United States | 50 standardized patients (SPs) working at Auburn University Harrison School of Pharmacy (AU HSOP) | Professionalism, patient-centered communication skills, and preparedness are critical factors influencing practice-readiness ratings of student pharmacists by standardized persons (SPs). These qualities and behaviors were consistently linked with different levels of practice readiness. However, the study suggests that further research is necessary to determine if these findings hold true across other educational institutions. |
| Friedlander, L.T., Wallace, W.D.A., Broadbent, J.M., Hanlin, S.M., Lyons, K.M., Cannon, R.D. & Cooper, P.R. Preparedness and competency of New Zealand graduates for general dental practice perceptions from the workforce. (2024) | To explore the preparedness of new graduates from the perspective of experienced clinicians who employ and/or mentor them and identify themes reflecting graduates’ preparedness for independent practice. | A mixed-methods | New Zealand | 83 registered dentists | The dental graduates were generally well-prepared for independent practice, especially in basic dentistry, communication, ethics, and record-keeping. However, they were perceived to be less proficient in complex treatment planning, molar endodontics, fixed prosthodontics, and exodontia due to limited clinical exposure during their training. |
| Graham P, Padley J, Williams S, Gonzalez-Chica D, Isaac V, & Walters L. Australian rural medical students' perceived readiness for work as a junior doctor: A cross-sectional national survey. (2023) | To report self-perceived readiness for work as a junior doctor in a national cohort of rural clinical school students, and to evaluate the relationship between readiness and students' perceived experiences of the clinical placement, including support, mentorship and clinical placement experience | Quantitative | Australia | 668 medical students; 44% male and 56% female; more than half of the respondents were less than 24 years of age | A significant majority felt well-prepared to begin work as junior doctors. While these students reported high confidence in clinical tasks, they expressed lower confidence in handling professional challenges such as addressing workplace bullying or raising concerns about colleagues. Factors contributing to their overall readiness included feeling integrated within their clinical teams, receiving academic support, opportunities for supervised practice, guidance from rural mentors, and prior qualifications in health professions. |
| Grant, S., Sheridan, L. & Webb, S.A. Newly qualified social workers' readiness for practice in Scotland. (2017) | To map the experiences of newly qualified social workers (NQSWs) entering first employment from degree training programs, identify components that impact on their continuing professional development in the workplace, examine the perspectives of recently qualified social workers related to preparedness to enter professional social work practice and investigate NQSW experiences of post-qualifying support and learning | Mixed-methods | Scotland | 205 newly qualified social workers | While most NQSWs felt adequately prepared by their social work education, they perceived less support from employers regarding opportunities for continuous professional development. Experiences with induction, supervision, and access to professional development opportunities varied across different regions of Scotland. |
| Grimm K, & Barker S. A Pandemic's Impact: Newly Licensed Nurse Self-Efficacy Following Increased Use of Simulation. (2022) | To identify the difference in self-efficacy in nursing practice of newly licensed nurses who transitioned to acute care during the pandemic to those who transitioned prior to the pandemic. | Quantitative | United States | 45 newly licensed nurses;  22-42 years old; 16% male and 84% female participants | There was no significant difference in overall self-efficacy scores between newly licensed nurses who transitioned before the pandemic and those who transitioned during it. However, nurses who transitioned during the pandemic had significantly higher self-efficacy in areas such as comfort and confidence with death and dying and finding nursing exciting and challenging. The increased use of simulation during the pandemic may have provided positive personal mastery experiences for nursing students, leading to higher self-efficacy in these specific areas. |
| Gruenberg K, Hsia S, O'brien B, & O'sullivan P. Exploring multiple perspectives on pharmacy students' readiness for advanced pharmacy practice experiences. (2021) | To explore the perspectives of APPE students, preceptors, and faculty site directors on pharmacy students' readiness for advanced pharmacy practice experiences (APPEs) | Qualitative | United States | 24 pharmacy students, 22 preceptors and 6 faculty directors | The study showed that APPE readiness encompasses several key facets, including learner characteristics, active participation in workplace activities, building effective relationships, and implementing supportive practices for student orientation and ongoing support. Learner attributes, active engagement in clinical settings, and fostering meaningful connections are essential aspects of APPE readiness. |
| Harrison H, Birks M, Franklin RC, & Mills J. Fostering graduate nurse practice readiness in context. (2020) | To examine the impact of the healthcare environment on graduate nurses' practice readiness and identify factors that assist graduate nurses to become practice ready. | Qualitative | Australia | 43 nursing, 11 medicine, 8 allied health, and 5 human resources participants | Graduate nurses undergo a progressive development of practice readiness from their initial nursing education and through their first year in practice. Achieving readiness for practice depends on factors within both educational programs and workplace settings. The implementation of a structured 12-month transition program specifically designed for graduate nurses, supported by workplace environments, plays a pivotal role in fostering their readiness for professional roles. |
| Haruzivishe, C. & Macherera, D.M. Perceived Readiness to practice among BSC Honors in Nursing graduates: Implications for training. (2021) | To assess the readiness to practice among BSc Nursing graduates | Mixed methods | Zimbabwe | 118 BSc nursing graduates; mean age of 31 years; 47.5% male and 52.5% female | The majority of the graduates demonstrated high confidence in their ability to care for patients on medical-surgical units. However, they expressed lower confidence in skills related to writing reflective journals or logs to develop critical thinking abilities. Additionally, a significant proportion of graduates felt that they lacked adequate support from their training institutions upon entering employment. |
| Harvey, P., Nightingale, C., & Kippen, R. Rural medical students' self-reported perceptions of preparedness to practice in the Aboriginal and Torres Strait Islander Health Context. (2021) | To investigate medical students' perceptions of preparedness to work in the Aboriginal and Torres Strait Islander health context, and explore their perceptions of what would improve their preparedness | Mixed methods | Australia | 106 medical students; 60% female | Medical students' readiness to work with Aboriginal and Torres Strait Islander peoples improves significantly as they advance through their clinical training due to prior learning, personal interactions with Indigenous communities, and direct patient care experiences. Students expressed a strong desire for more clinical placements and interactions with Indigenous peoples. |
| Hatzenbuhler NJ, & Klein JE. Educational Preparation for Clinical Practice Reflections of Newly Graduated RNs. (2019) | To explore the perceptions of newly graduated RNs about how their educational experiences as prelicensure students prepared them to enter the nursing workforce | Qualitative | United States | 10 registered nurses (RNs); 22-32 years old; 4 male and 6 female RNs | The study showed that nursing education programs are perceived as falling short in adequately preparing graduates for the diverse roles and responsibilities of RNs in clinical settings. Participants emphasized the need for nursing faculty to integrate more practice-oriented educational experiences to better equip students for the realities of clinical practice. It was recommended that these programs prioritize preparing students to confidently assume professional roles and responsibilities upon entering the nursing workforce. |
| Hyun A, Tower M, and Turner C. Exploration of the expected and achieved competency levels of new graduate nurses. (2020) | To explore the expected and achieved competency levels of new graduate nurses, compare the expected competency levels rated by educators vs. managers, and compare the achieved competency levels perceived by new graduates vs. graduating students | Mixed methods | Korea | 97 academic educators, 31 managers, 70 new graduate nurses and 179 graduating nursing students | The new graduate nurses demonstrated strong preparedness in respecting patient dignity; however, they expressed a need for additional support in delivering emergency care. Compared to graduating students, new graduates reported feeling less confident in competencies pertaining to legal and ethical practices. There was a disparity in expectations regarding the competency levels of new graduates between educators and managers. |
| Illing, J.C., Morrow, G.M., Rothwell nee Kergon, C.R., Burford, B.C., Baldauf, B.K., Davies, C.L., Peile, E.B., Spencer, J.A., Johnson, N., Allen, M. & Morrison, J. Perceptions of UK medical graduates' preparedness for practice: A multi-centre qualitative study reflecting the importance of learning on the job. (2013) | To explore whether UK medical graduates were prepared for medical practice | Qualitative | United Kingdom | 65 medical graduates; 38% male and 62% female; 85% were <30 years | Preparedness for practice as a junior doctor was closely linked to the extent and quality of experiential learning, especially involving active participation in clinical teams and supervised practice. |
| James PB, & Cole PC. Intern pharmacists' perceived preparedness for practice, their extent of involvement in pharmacy related activities and future career choices in Sierra Leone: A baseline descriptive survey. (2016) | To assess intern pharmacists' perceived preparedness for practice, document the extent of their involvement in selected pharmacy related activities during the internship period, and evaluate their future career path. | Quantitative | Sierra Leone | 20 intern pharmacists; 30±3 years old; 16 male and 4 female | Intern pharmacists in Sierra Leone generally felt adequately prepared for and actively engaged in various pharmacy-related activities, though they perceived a gap in participation within multidisciplinary team care. Despite this, the majority expressed a preference for more clinically oriented settings. However, pharmacists in Sierra Leone are predominantly involved in drug regulation, procurement, and community or academic settings rather than in direct patient care. This discrepancy highlights the mismatch between interns' career aspirations and the prevailing roles available in the local pharmacy sector. |
| Jamieson, I., Sims, D., Basu, A. & Pugh, K. Readiness for practice: The views of New Zealand senior nursing students. (2019) | To explore senior nursing students' perceptions of their readiness to practice as registered nurses, validate the Casey-Fink Readiness for Practice Survey in the New Zealand context | Mixed methods | New Zealand | 245 senior nursing students; 9% male and 90% female; majority aged 30 years or younger (73%) | Students generally felt confident and well-prepared across various aspects of nursing practice, including using evidence-based approaches, problem-solving, and identifying safety risks. However, they reported discomfort in specific areas such as caring for dying patients and feeling overwhelmed when managing more than three patients simultaneously. |
| Javed M, Nawabi S, Bhatti U, Atiqu S, Alattas M, Abulhamael A, Zahra D, & Ali K. How well prepared are dental students and new graduates in Pakistan: A cross-sectional national study. (2023) | To evaluate the self-perceived preparedness of undergraduate dental students and new graduates (house officers) in Pakistan. | Quantitative | Pakistan | 862 house officers and undergraduate dental students; 25.4% male and 74.6% female; 20-30 years old | Dental students and new graduates in Pakistan reported shortcomings in both clinical competencies and soft skills, scoring lower overall compared to studies from other countries. Specific areas of weakness identified included deficiencies in performing endodontic treatments, radiography skills, assessing orthodontic treatment needs, and providing crowns and cast partial dentures. The study also noted gender disparities, with female students generally feeling more prepared in certain clinical and professional skills compared to their male counterparts. |
| Kasita, R.E.N., Daniels, E.R. & Karera, A. Preparedness to assume professional roles: experiences of recently qualified radiographers: A qualitative study. (2023) | To explore and describe the lived experiences of recently qualified radiographers from the local university on their readiness to assume their professional roles | Qualitative | Namibia | 10 diagnostic radiographers; 3 male and 7 female; 22 to 27 years | There were positive and negative experiences of recently qualified radiographers. The positive experiences were increased confidence and creativity, responsibility consciousness, and effective teamwork. The negative experiences were reality shock from excessive workload, patient care impediments, burden of student supervision, and lack of professional trust. |
| Kinnane, P., Kennedy, N., & Quinton, A. Work readiness attributes: Comparative views of clinical supervisors and final year sonography students. (2021) | To determine what personal, interpersonal, and professional attributes clinical supervisors think are important for work readiness, and to compare the views of clinical supervisors to those of final year student sonographers | Quantitative | Australia | 65 clinical supervisors; 23% male and 77% female.  39 final year sonography students: 13% male and 87% female; 21-40 years | There was general alignment on the importance of social intelligence and work competence attributes for work readiness according to the clinical supervisors and final year sonography students. However, clinical supervisors placed greater emphasis on attributes related to personal qualities and understanding organizational dynamics, while, final year sonography students tended to prioritize technical competence and clinical skills over these aspects. |
| Kuzmenko, N. V., Ivanytska, T. A., Poda, O. A., Nesina, I. M., & Tanianska, S. M. Perception of readiness of future doctors for professional activities and determination. (2023) | To determine the readiness level and factors that influence the readiness of higher medical education students for future professional activities | Quantitative | Ukraine | 322 students of higher medical education; mainly ≤25 years old (67.6%) | The survey identified gaps in critical skills necessary for future work, alongside deficiencies in competence development and readiness to perform professional tasks. Medical students felt most prepared in patient-oriented abilities and advanced counselling skills and demonstrated high personal and professional skills. Limitation of the practical component of training was the most critical obstacle to acquisition of skills. |
| Lagali-Jirge, V., and Umarani, M. Evaluation of readiness to practice among interns at an Indian dental school. (2014) | To determine the readiness to practice after graduation among interns at a dental school in India. | Quantitative | India | 51 dental interns | 96% of dental interns expressed the need for further exposure under supervision before feeling ready to establish their own practice. Despite completing their internship, 88% of interns felt inadequately prepared in terms of knowledge and confidence to practice independently. Specific areas of concern included a lack of confidence in performing complex dental procedures such as root canal treatments, third molar extractions, and managing medically compromised patients. |
| Lanahan, M., Montalvo, B., & Cohn, T. The perception of preparedness in undergraduate nursing students during COVID-19. (2022) | To explore the perception of preparedness in nursing students who transitioned to online and virtual learning platforms brought about by Coronavirus disease 2019 (COVID-19) | Mixed methods | United States | 103 undergraduate nursing students; 4.9% male and 95.1% female; 30. 8±7.85 years | During the COVID-19 pandemic, the shift from in-person to online and virtual learning had a detrimental effect on nursing students' perceptions of preparedness for practice. The transition was marked by increased school support, which positively correlated with students' comfort in performing nursing skills. Conversely, greater patient assignments during this period were linked to decreased comfort levels. Reductions in clinical hours exacerbated challenges in learning techniques and dealing with challenges. |
| Lazarus, G., Findyartini, A., Putera, A.M., Gamalliel, N., Nugraha, D., Adli, I., Phowira, J., Azzahra, L., Ariffandi, B. and Widyahening, I.S. Willingness to volunteer and readiness to practice of undergraduate medical students during the COVID-19 pandemic: a crosssectional survey in Indonesia. (2021) | To evaluate the readiness to practice and willingness to volunteer of undergraduate medical students in Indonesia during the COVID-19 pandemic | Quantitative | Indonesia | 2374 undergraduate medical students; median age of 20 years (IQR: 19-21); 30.2% male and 69.8% female | Many undergraduate medical students expressed willingness to volunteer during the COVID-19 pandemic, but only a minority felt adequately prepared for independent practice. Factors such as sex, prior volunteering experience, university type, location, and socioeconomic status independently influenced students' readiness and willingness to volunteer. The main motivations for volunteering included addressing the shortage of medical staff and a sense of moral duty, while concerns about personal health risks and uncertainties about effective treatments were significant deterrents among students. |
| Leufer, T., & Cleary-Holdforth, J. Senior nursing students' perceptions of their readiness for practice prior to final year internship Part 2-A qualitative perspective. (2020) | To explore final-year nursing students' perceptions of their readiness for practice prior to their final internship | Qualitative, | Ireland | 24 final-year undergraduate nursing students | Students reported that they were not ready for professional practice, mainly in medication management, patient caseload management, and communication skills. They attributed their unease partly to challenges in clinical settings, such as staffing shortages, which restrict their exposure to and practice of complex nursing duties. The students perceive a tendency among qualified nurses to delegate basic tasks to them, potentially hindering their ability to fully develop necessary competencies before graduation. |
| Li, J., Huang, Y., Fong, D. Y. T., Chen, J., & Song, Y. Work readiness: Its determinants and association with work-related outcomes among new graduate nurses. (2022) | To identify determinants of work readiness among new graduate nurses, and assess the influence of work readiness on occupational commitment, coping self-efficacy and Intention to remain | Quantitative | China | 794 graduate nurses;8.3% male and 91.7% female | The study reported that demographics, education, social support, and environmental conditions influence how well-prepared nurses feel to handle their professional roles upon graduation. Enhancing work readiness was shown to positively impact coping self-efficacy, occupational commitment, and intention to remain in the nursing profession. |
| Lim SH, Ang SY, Aloweni F, Siow KCE, Bee S, Koh L, & Ayre TC. Factors associated with practice readiness among newly qualified nurses in their first two years of practice. (2024) | To provide an overview of the perception of practice readiness of newly graduated nurses for their professional nursing role and examine associations between nurses' readiness with individual demographic and occupational variables, and reasons for choosing the nursing profession. | Quantitative | Singapore | 445 registered nurses; 11% male and 89% female; 24±4.5 years | More than half of the newly qualified nurses expressed discomfort with independently performing complex clinical skills and procedures. Their primary concerns included facing ethical challenges and ensuring clinical competency. Nurses in their second year of practice indicated a higher level of confidence in problem-solving, caring for terminally ill patients, and prioritizing care needs compared to the first-year nurses. |
| Mak, V. S., March, G., Clark, A., & Gilbert, A. L. Intern pharmacists perceived preparedness for practice and their expectations. (2013) | To explore Australian intern pharmacists' perceived preparedness for practice, explore the match between intern pharmacists' expectations and experiences in meeting the requirements of Australia's health care reforms and evaluate intern pharmacists' future career intentions. | Quantitative | Australia | 60 intern pharmacists; median age of 23 years; 27% male and 73% female | Pharmacy graduates reported feeling well-prepared for patient care, providing medicines information, and roles in primary healthcare, but their internship experiences revealed a disparity between expectations and reality. By the end of the internship, an increase occurred in the proportion of respondents expressing a desire to pursue career paths other than practicing pharmacy, rising from 38% at the start to 45%. Additionally, uncertainty about future career directions also grew, with the percentage of undecided respondents increasing from 35% initially to 50% by the internship's conclusion. |
| Malau-Aduli, B.S., Jones, K., Alele, F., Adu, M.D., Drovandi, A., Knott, G., Young, L. & Jo, C. Readiness to enter the workforce: perceptions of health professions students at a regional Australian university. (2022) | To investigate perceptions of readiness for clinical practice and identify key factors that influence work-readiness among final-year medical, dental and pharmacy students | Mixed methods | Australia | 132 medical, dental, and pharmacy students; 38.6% male and 61.4% female; 20-39 years old | Health professions students reported that quality clinical placements and support systems from supervisors, family, and peers play a critical role in enhancing their preparedness for professional practice and improving patient outcomes. They identified challenges with readiness for private practice settings and noted that heavy workloads during placements hindered their preparation. |
| Mariño, R., Delany, C., Manton, D., Reid, K., Satur, J., Crombie, F., Wong, R., McNally, C., Lopez, D., Celentano, A. & Lim, M. Preparedness for practice of newly qualified dental professionals in Australia -educator, employer, and consumer perspectives. (2022) | To explore perceptions of different observers (educators, employers, consumers) regarding the preparedness to practice and work readiness of newly qualified dental professionals, and develop a more comprehensive understanding of dental professionals' preparedness for practice | Qualitative | Australia | 19 participants: 9 clinical supervisors (2 oral health therapists, 5 dentists, 2 prosthetists), 4 dental course convenors, 2 representatives of large public employers and 4 consumers; 12 male and 7 female | The study showed that dental students receive sufficient theoretical and evidence-based training to prepare for professional practice, although some skills would benefit from further training, consolidation, and higher levels of experience. While new graduates are generally ready to practice safely, they would benefit from additional transitional support in certain areas to enhance their competency and confidence. |
| Meyer, G. & Shatto, B. Resilience and transition to practice in Direct Entry nursing graduates. (2018) | To examine the relationship between resilience and transition to practice in Direct Entry Accelerated Master’s in Nursing (DEAMSN) graduates. | Quantitative | United States | 17 nursing graduates; 29.7±9.03 years; 2 male and 15 female | Graduates exhibited moderately high resilience upon graduation, although they scored lowest in equanimity and existential aloneness. Resilience was identified as a significant factor, explaining 79% of the variance in transition to practice scores at the 12-month mark post-graduation. |
| Missen, K., McKenna, L.& Beauchamp, A. Work readiness of nursing graduates: current perspectives of graduate nurse program coordinators. (2015) | To explore the perceptions of graduate nurse program coordinators on the work readiness of new nursing graduates and the challenges new nursing graduates experience in their first year of practice | Qualitative | Australia | 16 graduate nurse program coordinators; 3 male and 13 female | The study reported several challenges faced by new nursing graduates upon entering professional practice, especially the inadequacy of clinical skills and basic nursing knowledge among these graduates. Poor communication skills and issues with professional behavior were identified as common deficiencies that could impact patient care quality. Graduates who had prior experience as enrolled nurses faced greater difficulties transitioning to the registered nurse role compared to their peers without such experience. |
| Monrouxe, L.V., Bullock, A., Gormley, G., Kaufhold, K., Kelly, N., Roberts, C.E., Mattick, K. & Rees, C. New graduate doctors' preparedness for practice: a multistakeholder, multicentre narrative study. (2018) | To explore how stakeholders conceptualize "preparedness for practice", and understand the aspects in which new medical graduates are perceived as prepared or unprepared for clinical practice | Qualitative | United Kingdom | 175 stakeholders: The doctors (n=57) were mainly 25-34 years (74%) and female (62%). The healthcare stakeholders (n=93) were mainly 30-59 years (79%) and male (58%) The patient and public representatives (n=25) were aged 60+ years and 68% were female | Stakeholders involved in assessing preparedness for medical practice held diverse views that encompassed short-term, long-term, practical, and emotional dimensions. These perceptions varied significantly across different General Medical Council (GMC) outcomes and among different stakeholder groups. In some cases, medical graduates viewed themselves as more prepared than how other stakeholders assessed them. |
| Muruvan, C., Downing, C. & Kearns, I.J. Preparedness for practice: Experiences of newly qualified professional nurses in a private hospital setting. (2021) | To gain an understanding of newly qualified professional nurses' experiences of preparedness in private practice in the Southwest region of Johannesburg, South Africa | Qualitative | South Africa | 8 newly qualified female professional nurses; 25-41 years old | Newly qualified professional nurses experienced a lack of preparedness for leadership and responsibility, leading to self-doubt about their competence. They needed to acquire various skills to engage effectively with different healthcare team members. Time was essential for these nurses to develop into confident professionals. |
| Musallam, E., & A. Flinders, B. Senior BSN students' confidence, comfort, and perception of readiness for clinical practice: the impacts of COVID-19. (2021) | To explore which clinical skills senior BSN students felt least comfortable with due to COVID-19 modifications to their education, explore students' perceptions of readiness for professional nursing practice when their education was modified due to COVID-19, and explore the impacts of COVID-19 on senior BSN students' preparation for the National Council Licensure Exam (NCLEX) and initial clinical practice | Quantitative | United States | 26 nursing students; 26.8±5.1 years; 15.4% male and 84.6% female | Students highlighted significant adverse effects of the COVID-19 pandemic on their clinical training, skill acquisition, NCLEX preparation, and career trajectories. The pandemic notably reduced their confidence in managing multiple patient assignments, communicating effectively with physicians, responding to acute changes in patient conditions, and providing end-of-life care. |
| Mustakallio S, Näpänkangas R, Narbutaite J, & Virtanen J. Graduating dentists' perceptions about their professional competence in Finland and Lithuania. (2020) | To explore graduating dentists' perceptions about their professional readiness for clinical work in Finland and Lithuania | Quantitative | Finland and Lithuania | 515 fifth-year dental students | Graduating dental students from Finland and Lithuania expressed confidence in their ability to handle clinical procedures aligned with the ADEE competencies, particularly in tasks commonly encountered in dental practice. Finnish students felt most competent in producing and maintaining accurate patient records, administering block anesthesia, and ensuring sterilization and hygiene protocols. In comparison, Lithuanian students were confident in producing and maintaining patient records and maintaining sterilization and hygiene standards. Significant differences in perceived competence were noted between Finnish and Lithuanian students, as well as between Lithuanian students and their international counterparts. |
| Nelson, C., Mandrusiak, A. & Forbes, R. Perceived preparedness and training needs of new graduate physiotherapists working with First Nations Australians. (2023) | To explore the perceptions of new graduate physiotherapists regarding their preparedness for working with First Nations Australians | Qualitative | Australia | 13 new graduate physiotherapists | The new graduate physiotherapists reported adequacy of their pre-professional training to effectively work with First Nations Australians. However, they found that engagement in work-integrated learning (WIL) and ongoing on-the-job development was beneficial in enhancing their capability to provide culturally safe care. The study recommended a comprehensive training continuum that spans both pre-professional education and ongoing professional development. |
| Nweke, C.I., Abazie, O.H., Adetunji, A.J. & Okwuikpo, M.I. Readiness for clinical practice amidst coronavirus among nursing students in southwest Nigeria. (2021) | To assess the readiness of nursing students in southwest Nigeria to resume clinical practice amidst the coronavirus pandemic, and determine if there is a difference in readiness between nursing institutions and between years of clinical exposure | Quantitative | Nigeria | 300 nursing students; 22.16±3.11 years | 61.7% of participants felt prepared for clinical practice during the COVID-19 pandemic, but only 30.7% felt ready to practice in environments with COVID-19 patients. The readiness varied significantly across different nursing institutions, likely influenced by geographic location and varying levels of exposure to COVID-19 cases among the institutions. |
| O'Brien, K, Moore, A., Hartley, P., & Dawson, D. Lessons about work readiness from final year paramedic students in an Australian university. (2013) | To investigate the perceptions of readiness for the workforce in final year paramedic students at Victoria University, evaluate the strengths of the VU paramedic course and how the VU paramedic course could be improved to facilitate the transition into the workplace | Mixed methods | Australia | 23 final year paramedic students; 23±5.8 years; 12 male and 10 female | Final year paramedic students generally perceive themselves as adequately prepared or somewhat prepared for transitioning into the workforce. However, they advocated for improvement in their education, particularly through increased clinical placements across a broader spectrum of healthcare environments. |
| O'Brien, M., Troy, K., & Kirkpatrick, J. The allied health work readiness study: Identifying personal characteristics signaling work readiness in allied health students. (2020) | To identify the personal characteristics of allied health students that contribute to work readiness, and gain insights into which of these characteristics are considered most important for allied health careers | Qualitative | Australia | 18 allied health professionals | Personal insight and self-awareness, resilience, communication skills, organizational skills, lifelong learning, and professionalism were the main attributes across all groups. An additional nine characteristics were deemed important by two of the groups, while 22 more characteristics were highlighted by one group each. Many of these attributes were noted to be personal, personality-based, and focused on emotional intelligence. |
| Oluwatosin L, and Ogundero, A. Career and Work Readiness of Nutrition and Dietetics Trainees in Nigerian Universities. (2021) | To assess the readiness of trainees in the field of Nutrition and Dietetics from Nigerian Universities for career and work engagements. | Quantitative | Nigeria | 109 final-year undergraduate and postgraduate students in nutrition and dietetics programs; 27% male and 73% female; average age 23.8±3.4 years | Most nutrition and dietetics trainees expressed eagerness to embark on careers in their field; however, many faced challenges regarding their preparedness and understanding of necessary skills. While many trainees are willing to begin their professional journey, only half perceive themselves as adequately ready for work and career engagement. Additionally, many trainees lack clarity on the specific skills and competencies essential for their careers. |
| Opoku EN, Niekerk LV, Jacobs A, & Khuabi N. Exploring the transition from student to health professional by the first cohort of locally trained occupational therapists in Ghana. (2021) | To explore the experiences of the transition from student to health professional by the first cohort of locally trained occupational therapists in Ghana | Qualitative | Ghana | 6 occupational therapists | The initial cohort of occupational therapists in Ghana encountered substantial obstacles during their transition from students to professionals, including feelings of self-doubt, a perceived lack of preparedness for independent practice, difficulties in functioning without supervision, and challenges in integrating into the workplace environment. The participants acknowledged the importance of ongoing professional development and expressed optimism about the future of occupational therapy in Ghana. |
| Ottrey E, Rees CE, Kemp C, Brock TP, Leech M, Lyons K, Monrouxe LV, Morphet J, & Palermo C. Exploring health care graduates' conceptualisations of preparedness for practice: A longitudinal qualitative research study. (2021) | To explore health care learners' solicited and unsolicited conceptualizations of preparedness for practice (P4P) over their early graduate transition, and identify longitudinal patterns in P4P conceptualizations across the new graduate transition | Qualitative | Australia | 87 final-year students from four health care disciplines: dietetics, medicine, nursing, and pharmacy; median age of 23 years; 20% male and 80% female | The study identified 13 distinct conceptualizations of preparedness for practice among healthcare learners. Certain conceptualizations were prevalent in both solicited and unsolicited discussions, while others were more prominent in either solicited or unsolicited contexts. The dominance of these conceptualizations varied throughout the transition from learner to practitioner. |
| Phan A, Tan S, Romany M, Mandrusiak A, & Forbes R. Exploring new-graduate physiotherapists' preparedness for, and experiences working within, Australian acute hospital settings. (2023) | To investigate new-graduate physiotherapists' experiences of working in acute hospital settings, and their perceptions of how their pre-professional training prepared them for working in acute hospital settings | Qualitative | Australia | 14 new graduate physiotherapists; 22-25 years old; 3 male and 11 female students | New graduate physiotherapists encounter distinct and often daunting challenges when transitioning into acute hospital settings, despite their foundational skills acquired during pre-professional training. They face difficulties associated with managing higher patient caseloads and handling more complex cases than they did as students. Additionally, these new graduates face challenges in effectively communicating and collaborating within multidisciplinary teams in acute care environments, which are essential for ensuring safe and optimal patient care outcomes. |
| Phillips, K.E., Dzurec, L., Burgess, A., Beauvais, A. & McNutt-Clarke, B. Ramifications of the COVID-19 pandemic on nursing students' transition to practice. (2023) | To examine clinical nursing faculty members’ and preceptors’ assessment of senior nursing students’ practice strengths and challenges compared to those of graduates of prior years’ whose clinical experiences were not disrupted by COVID-19 | Mixed methods | United States | 25 clinical faculty members; 40±13 years | According to clinical faculty and preceptors, COVID-19 had a significant positive and negative impact on 2021 nursing graduates' learning, skill development, and transition to practice. They reported that graduates would benefit from clinical support through extended orientations and mentoring. |
| Piccuito, C.M. & Santiago, R.R. New graduate respiratory therapists' perceptions of their transition to practice. (2023) | To explore the experiences of new graduate respiratory therapists (RTs) during their first year of practice and identify barriers and facilitators to a successful transition to practice. | Qualitative | United States | 28 new graduate respiratory therapists; 18-54 years; 21% male and 79% female | New RTs encountered many challenges during their transition to practice, including insufficient orientation, inadequate leadership support, high stress levels, overwhelming workloads, and instances of negative workplace behavior. These challenges had significant implications for job satisfaction, retention rates, and patient care quality. Improving transition-to-practice programs and implementing surveys to gather feedback from new graduate RTs could enhance their experience, potentially leading to higher job satisfaction and improved patient outcomes. |
| Powers, K., Montegrico, J., Pate, K. & Pagel, J. Nurse faculty perceptions of readiness for practice among new nurses graduating during the pandemic. (2021) | To quantitatively describe and compare nurse faculty perceptions of readiness for practice among students who graduated pre-pandemic and those who will graduate during the pandemic, and provide guidance to transition-to-practice programs based on the findings | Quantitative | United States | 116 nurse faculties; 8.6% male and 91.4% female; mainly 30-69 years old (94%) | Nurse faculty reported a decline in the readiness for practice among new graduate nurses who completed their education during the COVID-19 pandemic compared to those who graduated before it. Technical skills, critical thinking, management of responsibilities, and communication were specific areas where mean competency scores decreased significantly. |
| Pullen, D., & Ahchay, D. A case study of new nurses' transition from university to work. (2022) | To explore the perceived factors that affect how well new graduate nurses were prepared for transition to practice as a registered nurse, identify what can be done during clinical placement processes to improve outcomes for nursing undergraduates and potential employers, and determine what elements can be influenced or taken into consideration by clinical placement teams to ensure that the nursing students' clinical experience is more relevant and applicable to give them a better chance of a seamless transition | Quantitative | Australia | 16 new graduate nurses; 21-40 years old | This study showed that a successful transition from undergraduate to new graduate nurse is facilitated by varied clinical placements during university, which expose students to different patient acuity, complexity, workload, and settings. Transition to Practice Programs that build on new graduates' previous undergraduate clinical experiences further enhance this transition. However, many new graduate nurses (31%) did not have adequate opportunities to practice skills in labs prior to clinical placements. |
| Reynolds K, & Mclean M. Clinical supervisors' perceptions of podiatry students' preparedness for clinical placement and graduates' preparedness for podiatry practice in Australia: An exploratory study. (2021) | To explore clinical supervisors' perceptions of podiatry students' preparedness for clinical placements, explore clinical supervisors' perceptions of podiatry graduates' preparedness for clinical practice, and the perceived challenges for student clinical placement preparation and related recommendations for clinical placement and graduate practice | Qualitative | Australia | 11 clinical supervisors; 5 male and 6 female; average age 40.5±7.8 years | Clinical supervisors in podiatry expressed varied views on the readiness of students for clinical placements, with opinions ranging from some supervisors perceiving students as adequately prepared, others uncertain, and some viewing them as unprepared. Key challenges identified included students' insufficiently developed clinical skills and low self-efficacy, primarily attributed to limited hands-on experience during placements. To enhance students' preparedness for clinical roles, supervisors recommended improvements in the quality of clinical experiences offered during training, alongside targeted training in communication skills. |
| Rusch, L., Manz, J., Hercinger, M., Oertwich, A. & McCafferty, K. Nurse preceptor perceptions of nursing student progress toward readiness for practice. (2019) | To determine the strengths and weaknesses of senior-level nursing students related to readiness for practice before graduation, and to assess students' readiness for practice as perceived by nurse preceptors one term before graduation and entry into practice | Quantitative | United States | 569 nurse preceptor responses | Senior nursing students demonstrated strong performance in professional attributes but exhibited weaknesses in time management, prioritization, management of multiple patients, and pharmacology knowledge. Pharmacology knowledge emerged as a significant area of concern within the cognitive domain, |
| Shaw, P., Abbott, M. & King, T.S. Preparation for practice in newly licensed registered nurses: A mixed-methods descriptive survey of preceptors. (2018) | To examine current RN preceptor perceptions of newly licensed registered nurses’ (RNs') preparedness for practice. | Mixed methods | United States | 42 registered nurses’ preceptors | RNs exhibited notable strengths in caring/compassion, collaboration, communication, and overall preparedness to practice. However, they faced deficiencies in time management, prioritization, handling complex situations, and quick thinking. |
| Sheehan D, De Bueger T, Thorogood J, Sitters S, & Deo A. Beyond competencies -describing work ready plus graduates for the New Zealand medical imaging workforce. (2018) | To identify the capabilities most valued by health services that provide placement opportunities for medical imaging students, using an existing Professional Capability Framework, define "work ready plus" graduates for the medical imaging workforce by identifying the key capabilities required of medical imaging technologists in their graduate years, and inform the development of the clinical experience program, as clinical placements are seen as crucial for developing capability and work readiness skills in graduates | Quantitative | New Zealand | 52 senior medical imaging staff; 10% male and 90% female | The study showed that the medical imaging profession in New Zealand values a range of capabilities across personal, interpersonal, and cognitive domains. Personal attributes highly valued include the ability to stay composed under pressure, awareness of personal strengths and limitations, a commitment to learning from mistakes, a strong work ethic, and a genuine passion for the profession. Interpersonally, effective teamwork and collaboration skills are emphasized as crucial for success in the field. Cognitive capabilities deemed essential involve problem-solving skills. |
| Smith SM, Buckner M, Jessee MA, Robbins V, Horst T, & Ivory CH. Impact of COVID-19 on new graduate nurses' transition to practice: Loss or gain? (2021) | To examine the impact of COVID-19 on new graduate nurses' preparedness for professional practice. | Mixed methods | United States | 340 newly graduated nurses (NGNs) | During the COVID-19 pandemic, over two-thirds of NGNs encountered disruptions in their clinical training, leading to transitions to virtual or alternative clinical experiences. NGNs expressed concerns about potentially missing critical details or making errors in patient care. The study recommended clear communication with nursing leadership, active advocacy from nurse residency programs, and targeted initiatives to provide clinical and emotional support for NGNs. |
| Sterner, A., Eklund, A. & Nilsson, M.S. Prepared to learn but unprepared for work: A cross-sectional survey study exploring the preparedness, challenges, and needs of newly graduated nurses entering a hospital-based transition program. (2023) | To investigate new graduate nurses' (NGNs') transition to hospital care, including their perceptions of their preparedness, their perceived challenges and their expectations of a transition program | Mixed methods | Sweden | 248 nurses; 12.5% male, 87.1% female and 0.4% unknown; 21-53 years old | The NGNs perceived that their preparedness, the challenges they experience, and their needs are related and amplify each other.65% of NGNs felt that their nursing education prepared them for clinical work, but only 55% felt it prepared them well for practical aspects of nursing work. The NGNs expect transition programs to provide them with opportunities for knowledge development, understanding the hospital organization, role development, exchanging experiences with other NGNs, psychosocial support, and supervisor support. |
| Stoikov, S., Maxwell, L., Butler, J., Shardlow, K., Gooding, M., & Kuys, S. The transition from physiotherapy student to new graduate: are they prepared? (2022) | To explore the perspectives of new graduate and experienced physiotherapists on the transition from student to new graduate. | Qualitative | Australia | 38 new graduate physiotherapists; 26.3% male and 73.7% female; 20-30 years. 35 experienced physiotherapists; 40% male and 60% female; majority (65.7%) 26-35 years | The study showed that new graduate physiotherapists face considerable challenges transitioning to professional practice, primarily due to increase in caseload volume and complexity compared to their clinical placements as students. They reported feeling overwhelmed and unprepared for the demands of the full scope of physiotherapy responsibilities. They often placed significant pressure on themselves to meet the standards set by experienced colleagues, intensifying their feelings of inadequacy during this critical period of professional transition. |
| Stulz V, Rakime Elmir, Reilly H, & Francis L. A pilot study: Transitioning into a new graduate midwife perspectives about a unique student-led practice. (2023) | To explore midwifery students' experiences of a student-led midwifery group practice and to explore the same midwifery students' experiences at the completion of their final year and as they transitioned into their new graduate program | Qualitative | Australia | 4 midwifery students; 21-35 years old | Midwifery students valued spending a year in a student-led model of care, which allowed them to build and sustain important relationships with women and their teams, including their mentor midwife. This model helped students transition from being students to becoming confident and autonomous new graduate midwives. Through the support and mentorship of experienced midwives, the students developed important skills such as teamwork, communication, and clinical decision-making. |
| Tarhan, M., Doğan, P., & Kürklü, A. The relationship between nurse-nurse collaboration and work readiness among new graduate nurses. (2022) | To determine the perception of nurse-nurse collaboration among new graduate nurses (NGNs), determine their work readiness level and the relationship between nurse-nurse collaboration and work readiness among the NGNs | Quantitative | Turkey | 198 new graduate nurses; 23.4±1.1 years;16.2% male and 83.6% female | NGNs generally held moderate perceptions regarding nurse-nurse collaboration and their overall work readiness. There was a positive correlation between nurse-nurse collaboration and NGNs' readiness for work. Additionally, NGNs expressed higher perceptions of their personal work characteristics compared to other aspects of work readiness. |
| Thomas, D. & Merrill, K. Meeting Theory-to-Practice Gaps? Evaluation of New Graduates. (2023) | To evaluate the effectiveness of a nurse residency program in meeting theory-to-practice gaps for new nursing graduates, and identify the gaps between theory and practice for new nursing graduates in order to restructure the residency program | Mixed methods | United States | 198 newly graduated nurses, 76 before and 122 after the program; 80-83% female; 25-35 years old | Participation in a nurse residency program significantly enhances the transition to practice for new nursing graduates, leading to increased ratings of competency and confidence. The study emphasizes the importance of ongoing evaluation and restructuring of these programs to effectively bridge theory-to-practice gaps commonly experienced by new nurses. Specific areas identified for improvement include addressing skill deficiencies, enhancing unit support, and tailoring program elements to meet the unique needs of nurses specializing in various clinical areas. |
| Usher, K., Mills, J., West, C., Park, T. & Woods, C. Preregistration student nurses' self-reported preparedness for practice before and after the introduction of a capstone subject. (2015) | To assess changes in perceptions of confidence and preparedness for practice of preregistration nursing students before and after the introduction of a capstone subject, and examine factors associated with perceptions of preparedness | Mixed methods | Australia | 167 nursing students. 2012 cohort (113): 11% male and 89% female; median 27 years old (21, 38 interquartile range).  2013 cohort (54): 2% male and 98% female; median 25 years old (21, 36 interquartile range) | The students did not strongly identify with a professional identity, which they attributed more to the quality of clinical placements rather than the capstone subject. They reported a lack of confidence in performing invasive procedures and managing care for dying patients. Confidence levels tended to decrease as the complexity and size of patient care assignments increased, with older students showing less confidence in managing multiple patient assignments. |
| Waite, N.M., McCarthy, L., Milne, E., Hillier, C., Houle, S.K. & Dolovich, L. Perceived preparedness for full-scope pharmacist services among recent Doctor of Pharmacy graduates from Ontario schools of pharmacy. (2018) | To examine how recent Doctor of Pharmacy graduates feel about providing full-scope pharmacist services (such as prescribing and immunization) and whether characteristics such as recent graduates' institutional and personal demographics, practice setting (e.g., busyness of practice, time spent directly with patients), or additional education after graduation affect their perceptions of feeling able and sure to perform these services | Quantitative | Canada | 120 pharmacy graduates; average age 26.3 years; 51% female | The graduates reported feeling generally prepared to offer full-scope pharmacist services, although they often expressed greater certainty than actual ability in performing many of these services. There was a positive correlation between the frequency of providing a service and graduates' feelings of both capability and certainty in performing that service. Graduates pursuing further education after graduation tended to feel less confident and capable in providing certain services, potentially due to heightened self-awareness of their limitations and the complexities associated with advanced practice. |
| Walker, A., & Campbell, K. Work readiness of graduate nurses and the impact on job satisfaction, work engagement and intention to remain. (2013) | To investigate the relationship between work readiness and job satisfaction, work engagement, and intention to remain among graduate nurses | Quantitative | Australia | 96 graduate nurses; 6% male and 92% female; 21-52 years | Organizational acumen, clinical competence, and social intelligence contribute to different work outcomes such as job satisfaction, work engagement, and intention to remain in the profession. Organizational acumen influences intention to remain through its effects on job satisfaction and work engagement. The combined factors of organizational acumen and work engagement explain 21% of the variance in nurses' intention to remain in their jobs. |
| Walker, A., Yong, M., Pang, L., Fullarton, C., Costa, B., & Dunning, A. T. Work readiness of graduate health professionals. (2013) | To explore the strategies and skills that constitute work readiness (WR) in medical and nursing graduates, investigate how WR can impact the ability to deal with organizational issues, and examine how WR can influence the transition and integration experiences of medical and nursing graduates | Qualitative | Australia | 46 graduate health professionals: 15 medical graduates and 26 nursing graduates | The study showed that the concept of work readiness among graduate health professionals is categorized into four main domains: social intelligence, organizational acumen, work competence, and personal characteristics. Social intelligence, highlighted by effective communication and teamwork skills, is seen as essential for a smooth transition and successful integration into the workplace environment. Organizational acumen, encompassing an understanding of workplace dynamics and adaptability, also plays a crucial role in navigating professional settings. Work competence, which includes technical proficiency and clinical skills, ensures that graduates can perform their job duties effectively. Personal characteristics such as resilience are vital for managing the challenges and pressures of hospital environments, contributing to overall readiness and success in graduates’ professional roles. |
| Walters, G., Hoffart, N., Kring, D., Whitley, T., Horne, L., & Almotairy, M. Work readiness of newly licensed RNs. (2022) | To describe the work readiness of newly licensed registered nurses (NLRNs) participating in a nurse residency program (NRP) and determine if there are relationships between NLRN work readiness demographic characteristics, type of nursing degree, completion of a nurse externship program and previous healthcare work experience | Quantitative | United States | 262 newly licensed registered nurses; 27.6±7.45 years; 11.1% male and 88.9% female | NLRNs reported high levels of perceived work readiness, with the highest scores observed in the Organizational Acumen dimension. Black NLRNs scored higher on the Personal Work Characteristics dimension than White NLRNs, indicating potential cultural or contextual differences in self-perception. NLRNs who graduated from Associate Degree in Nursing (ADN) programs scored higher on both Personal Work Characteristics and Work Competence dimensions compared to their Bachelor of Science in Nursing (BSN) counterparts. Participants who had participated in nurse externship programs scored lower on the Work Competence dimension. |
| Watt E, & Pascoe E. An exploration of graduate nurses' perceptions of their preparedness for practice after undertaking the final year of their Bachelor of Nursing degree in a university-based clinical school of nursing. (2013) | To explore graduate nurses' perceptions of their preparedness for practice after undertaking the final year of their Bachelor of Nursing degree in a university-based clinical school of nursing | Qualitative | Australia | 10 registered nurses; 1 male and 9 female | The university-based clinical school model effectively prepared graduate nurses for professional practice by immersing them in hospital settings, integrating university resources, and fostering meaningful engagement with clinical practice. |
| Wells, C., Olson, R., Bialocerkowski, A., Carroll, S., Chipchase, L., Reubenson, A., Scarvell, J.M.& Kent, F. Work readiness of new graduate physical therapists for private practice in Australia: Academic faculty, employer, and graduate perspectives. (2021) | To explore academic faculty, employer, and recent graduate perspectives of the work readiness of Australian new graduate physical therapists for private practice and factors that influence new graduate preparation and transition to private practice. | Mixed methods | Australia | 27 academic faculty members; 45.6±10.2 years; 40% male and 60% female  53 employers; 41.6±11.1 years; 64% male and 36% female.  32 recent physical therapy graduates; 24.9±3.2 years; 53% male and 47% female | There was a gradual increase in readiness for private practice, with initial perceptions of readiness improving significantly by the third year of employment. The new graduates’ strengths were enthusiasm and up-to-date knowledge, while they were perceived to have weaknesses in clinical reasoning, business acumen, and interpersonal skills. Factors influencing transition to private practice encompassed practical experience gained, support received in the workplace, the quality of academic preparation, and individual attributes of the graduates themselves. |
| Wijnen-Meijer, M., Ten Cate, O., van der Schaaf, M., Burgers, C., Borleffs, J., & Harendza, S. Vertically integrated medical education and the readiness for practice of graduates. (2015) | To determine differences in readiness for clinical practice between graduates from a vertically integrated (VI) curriculum and those from a non-VI curriculum. | Quantitative | Netherlands and Germany | 59 medical students; 71% female; average age of 25.1 years | Graduates from a vertically integrated curriculum demonstrated stronger competencies in "active professional development" and excelled in performing tasks such as "solving a management problem." Conversely, graduates from a non-vertically integrated curriculum showed higher proficiency in delivering difficult news, "teamwork and collegiality" and "knowing and maintaining own personal bounds and possibilities". |
| Willman, A., Bjuresäter, K. & Nilsson, J. Newly graduated registered nurses' self-assessed clinical competence and their need for further training. (2020) | To explore and describe changes in self-assessed clinical competence of newly graduated registered nurses (NGRNs) during their first 15 months of work in acute care hospital settings, and explore changes in NGRNs' need for further training during their first 15 months of work in acute care hospital settings | Quantitative | Sweden | 45 newly graduated registered nurses; 25.5±4.5 years; 7.1% male and 92.9% female | NGRNs demonstrated an increase in self-assessed clinical competence over a 15-month period, with the highest scores reported in "clinical leadership" and the lowest in "critical thinking". Throughout this period, NGRNs expressed the need for additional training in "direct clinical practice". Development of "critical thinking" skills did not show as significant an improvement as other competencies and even exhibited a slight decrease towards the end of the study period. |
| Wong, W. J., Lee, R. F., Chong, L. Y., Lee, S. W. H., & Lau, W. M. Work readiness of pharmacy graduates: An exploratory study. (2023) | To determine the impact of the COVID-19 pandemic on the work readiness of pharmacy graduates | Mixed-methods | Malaysia | 9 pharmacy preceptors; 30-50 years old; 22.2% male and 77.8% female 30 pharmacy graduates; 23.7±0.98 years; 16.7% male and 83.3% female | Graduates faced challenges due to reduced clinical exposure during the pandemic, resulting in comparable clinical knowledge but diminished clinical skills, confidence, and communication abilities compared to previous cohorts. The pandemic's impact on clinical placements influenced graduates' career decisions. |
| Woods, C., West, C., Mills, J., Park, T., Southern, J., & Usher, K. Undergraduate student nurses' self-reported preparedness for practice. (2015) | To identify the skills and procedures third year nursing students lacked confidence in or found difficult to perform independently, ascertain student perception of preparedness for the registered nurse role, and collect baseline evidence of students' perceptions of confidence and preparedness to compare with phase two data collected post-introduction of the capstone subject | Quantitative | Australia | 113 student nurses; 20-56 years; 10.7% male and 89.3% female | Nursing students placed high value on clinical placements as essential for enhancing their preparedness for independent practice. They perceive caring for multiple patients as a skill that develops over time, with confidence and competence increasing as they gain experience. Students were not confident in managing caseloads involving 3 or more patients, and this lack of confidence tended to persist as they progressed through their education. |
| Woolley T, Clithero-Eridon A, Elsanousi S, & Othman A. Does a socially accountable curriculum transform health professional students into competent, work-ready graduates? A cross-sectional study of three medical schools across three countries. (2019) | To assess the biomedical and socially accountable medical schools in Australia, the United States and Sudan. | Quantitative | Australia, Sudan and United States | 184 supervising physician graduates from three medical schools | The socially accountable health professional education (SAHPE) medical graduates were rated as competent or more competent than graduates from traditional medical schools, particularly in socially accountable competencies. However, SAHPE graduates scored slightly lower than non-SAHPE graduates in certain aspects of biomedical knowledge and clinical reasoning and decision-making. |
| Zhang J, Makanjee C, Hayre C, & Lewis S. Australian graduate radiographers' perspectives and experiences of work readiness. (2023) | To understand the work readiness perspectives and initial experiences of graduate radiographers starting their newly qualified roles, and establish student, graduate, and supervising radiographers' perspectives and understandings of work readiness, focusing on the transition to professional practice | Qualitative | Australia | 14 newly qualified radiographers; <30 years old; 4 male and 10 female | New graduate radiographers' perceptions of readiness for work are shaped by several factors including their clinical placements, familiarity with the workplace, support from colleagues, and personal coping strategies. Opinions among new graduates vary regarding the level of support they prefer, with some favoring immersive experiences while others seek structured orientations. |
