# Supplementary material for: Readiness for professional practice among health professions education graduates: a systematic review
Source: Front Med (Lausanne). 2024 Nov 8;11:1472834. doi: 10.3389/fmed.2024.1472834 (PMC11583157; doi:10.3389/fmed.2024.1472834)
Supplement: Supplementary file 2 [file Table_2.docx]

**Supplementary Table 2. Summary of findings in the reviewed articles**

| **Title** | **Theoretical framework** | **Conceptualization of R2P (Definition of R2P where provided)** | **Measurement tool used** | **Factors influencing R2P** | **Strategies to enhance R2P** |
| --- | --- | --- | --- | --- | --- |
| Abuhussain, et al. 2021 | Not mentioned | Work readiness involves having the necessary skills, knowledge, and attributes to perform pharmacy roles, with a focus on practical application in real-world settings. | Work Readiness Scale | Type of pharmacy program (PharmD vs. BPharm), previous pharmaceutical marketing training, quality of educational preparation and training, and support from mentors and clinical supervisors. | Incorporation of advanced pharmacy training, including pharmaceutical marketing experience, enhanced clinical and practical training, structured mentorship and support, and regular assessment and feedback mechanisms. |
| Adam et al., 2014 | Successful transition to the workforce characterized by feelings of well-being and mastery, and dependent on professional relationships. | Capability to manage and prevent workplace injuries effectively  (“Successful transition to the workforce has been described as a state where distress is replaced with feelings of well-being and mastery.”) | Surveys and interviews | Knowledge of injury management, confidence, workplace safety culture, support from colleagues, training on injury prevention, and practical experiences | Training on injury prevention and management, and supportive workplace environment |
| Akinkugbe et al., 2020 | Not mentioned | Confidence levels in entering clinical practice or residency. | Online survey | Impacts of COVID-19 on education modalities, moving to online instruction, and limitations on patient care. | Providing training in tele dentistry for students. Responsive training and support to mitigate heightened anxiety and stress levels. |
| Almadani et al., 2024 | Not mentioned | Ability to transition from student to professional nurse | Casey-Fink Readiness for Practice Survey | Self-assessment of skills, previous clinical experiences, quality of mentorship, clinical environment, curriculum design, and hands-on clinical practice | Enhanced orientation programs, continuous assessment and feedback mechanisms |
| AlMekkawi and El Khalil, 2022 | Not mentioned | Readiness to practice involves the ability to manage patient care independently, communicate effectively with patients and interdisciplinary teams, solve clinical problems, and recognize significant changes in patient conditions. | The Casey-Fink Readiness for Practice Survey | Clinical experiences, support and feedback from supervisors, opportunities for hands-on practice in clinical settings, and theory-practice gaps in educational preparation. | Better learning opportunities in clinical settings, closer guidance and support from supervisors, on-time feedback in clinical settings, and collaboration between educators and clinical settings to align student learning opportunities with professional roles. |
| Almotairy, et al., 2022 | Not mentioned | Work readiness encompasses having the necessary clinical skills, knowledge, and professional attitudes to perform effectively in nursing roles. | Work Readiness Scale | Country and university from which students graduated, having a second job, whether nursing was their first choice of study, whether the hospital was the first preference and the number of weekly working hours | Enhancing clinical training and practical experiences, providing mentorship and continuous support, creating a supportive work environment, and regular assessment and feedback. |
| Al-Rawajfah et al., 2023 | Not mentioned | Ability to transition from education to professional practice effectively | Surveys and interviews | Clinical skills, confidence, support from colleagues, cultural factors, quality of nursing education, and practical training | Enhanced clinical training, and continuous professional development |
| Anokwuru and Daniels, 2021 | Not mentioned | Ability to deliver effective nursing care based on education received | Surveys and interviews | Confidence in clinical skills, adaptability, support from healthcare institutions, quality of mentorship, practical training, and relevance of curriculum | Continuous education and training programs, strengthening clinical partnerships and mentorship opportunities |
| Atkinson and McElroy, 2016 | Thorne’s interpretive description | Ability to handle the demands of private practice effectively | Interviews | Clinical skills, business acumen, support from colleagues, business environment, training on business aspects, and clinical placements | Training on business management, mentorship programs focusing on private practice skills |
| Attrill, et al., 2021 | Boundary Critique Theory and Yolles' extension of Boundary Critique Theory | Ability to apply academic knowledge in clinical settings  (“Having the skills and attributes that allow graduates to succeed in the workplace”). | Surveys and interviews | Self-confidence, communication skills, support from workplace, supervision quality, quality of clinical training, and curriculum alignment | Enhanced clinical placements, and improved supervisor training |
| Bäck et al., 2017 | Confidence and self-efficacy | Professional confidence in performing midwifery tasks | Surveys | Self-assessed competence, previous experience, support from clinical staff, workload, curriculum quality, and practical experience during training | Enhanced practical training, and supportive clinical supervision |
| Barr et al., 2017 | Alignment of the "work ready plus" concept with patient-centred medicine, and the view of preparedness as a continuous non-linear process that should be assessed through an integrated continuous assessment model. | Readiness to handle clinical responsibilities effectively | Surveys and focus groups | Clinical competence, confidence, support from clinical environment, quality of supervision, curriculum design, and practical training | Enhanced clinical placements, and improved support systems for graduates |
| Bradley et al., 2023 | Benner's "From Novice to Expert" theory | Readiness for practice involves having the necessary clinical skills, knowledge, and confidence to transition into professional nursing roles effectively. | Case studies, surveys, and interviews | Clinical skills and competence, support from faculty and mentors, quality of clinical placements and practical experiences, and educational curriculum and training. | Enhanced clinical training and practical experiences, structured mentorship programs, continuous assessment and constructive feedback, and integration of theory and practice in the curriculum. |
| Carter and Stoehr, 2019 | Not mentioned | Readiness includes clinical competence and professional skills | Questionnaire | Clinical skills, confidence, teamwork, continuous professional development, effective teaching methods, and practical training | More group dynamics and teamwork exercises, increased use of simulations and standardized patient encounters |
| Casey et al., 2024 | Not mentioned | Influence of the pandemic on the healthcare system and training. | Surveys and interviews. | Personal resilience and coping mechanisms, changes in clinical practice environments, and modifications in training programs. | Enhancing support systems and providing crisis-specific training. |
| Chesterton et al., 2023 | Not mentioned | Ability to apply theoretical knowledge to clinical situations | Survey | Self-efficacy, stress management skills, quality of clinical supervision, work environment, curriculum content, and practical training opportunities | Enhanced supervision, and more real-world scenarios in training |
| Clark et al., 2024 | Not mentioned | Being able to apply nutritional knowledge in practical settings | Surveys and interviews | Professional skills, confidence in applying knowledge, support from employers, workplace culture, practical training, and curriculum relevance | More diverse practical placements, enhanced collaboration between educational institutions and industry |
| Dlamini et al., 2014 | Levett-Jones and Lathlean’s ascent to competence conceptual framework | Capability to meet the demands of the healthcare system  (“Competencies new graduates must attain upon completion of training”). | Surveys and interviews | Personal resilience, adaptability, health system infrastructure, support from senior staff, relevance of curriculum, and quality of clinical placements | Integration of context-specific training modules, and strengthening of internship and residency programs |
| Dudley et al., 2019 | Not mentioned | Readiness for practice is influenced by the quality and characteristics of the clinical learning environment, including exposure to real-world scenarios and mentorship. | Surveys and interviews | Quality of clinical placements, support from mentors and clinical supervisors, opportunities to engage in hands-on practice, integration of theoretical knowledge with clinical practice. | Improving the quality of clinical placements, providing strong mentorship and support, enhancing the integration of theory and practice, and ensuring a supportive and well-resourced clinical learning environment. |
| Duijn et al., 2020 | Competency-based medical education and entrustable professional activities (EPAs) | The ability to perform core veterinary tasks with varying levels of supervision | Surveys | Clinical skills, self-confidence, support from colleagues, workload, quality of veterinary education, and practical training components | Continued professional development, enhanced mentorship and supervision in early career stages |
| Ersoy and Ayaz-Alkaya, 2024 | Not mentioned | Combination of knowledge, experience, clinical competencies, holistic patient care and ability to apply theoretical knowledge into practice, complemented by self-efficacy.  (“The level of preparedness of new graduates transitioning from student roles to professional roles”) | Academic Nurse Self-Efficacy Scale and the Casey-Fink Readiness for Practice Survey. | Age, gender, academic achievement, satisfaction with studying nursing, and practical applications such as clinical/field practice. | Increase practices, care plan preparation, case discussion, and simulation practices to prepare students for the profession. Integrating theory and practice in clinical settings, maintaining practices that raise students' sense of responsibility and self-efficacy, addressing academic and individual needs. |
| Farris et al., 2023 | Entrustable Professional Activities (EPAs) | Conceptualized through entrustable professional activities across various levels of supervision and independence | Advanced Pharmacy Practice Experiences readiness survey and Practice readiness survey. | Level of autonomy during training, exposure to real-world scenarios, and feedback mechanisms | Implementing structured assessments, increasing real-life practice exposure |
| Fejzic and Barker, 2015 | Not mentioned | Readiness for practice involves possessing both technical and transferable (soft) skills, as well as the ability to apply these skills in a practical setting. | Surveys and interviews | Technical knowledge and skills, soft skills such as communication and teamwork, practical experience in community pharmacy settings, and support from academic and clinical mentors. | Incorporation of more practical experiences in the curriculum, development of soft skills through targeted training programs, enhanced mentorship and support systems, regular assessment and feedback mechanisms. |
| Fenech et al., 2020 | The four-factor model by Caballero, Walker and Fuller-Tyszkiewicz | Work readiness involves possessing the required skills, knowledge, and attributes to perform effectively in various professional specializations.  (“Work readiness of graduates is the extent to which graduates are perceived to have the required skills and attributes to be successful at work in terms of performance and career advancement.”) | Work Readiness Scale | Personal work characteristics such as resilience, adaptability, and stress management, social intelligence including teamwork and communication skills, work competence comprising technical skills and knowledge, and organizational acumen involving professional development and work ethics. | Development of personal skills through training programs, enhancing social intelligence through teamwork and communication exercises, improving technical skills and knowledge through practical experiences, and fostering professional development and work ethics. |
| Forbes and Ingram, 2021 | The biopsychosocial model of health | Ability to manage complex conditions like chronic pain effectively | In-depth interviews | Confidence in managing chronic pain, communication skills, support from senior physiotherapists, interdisciplinary collaboration, training on chronic pain management, and case-based learning | Specialized training modules, and continuous professional development programs |
| Ford et al., 2021 | Not mentioned | Standardized person patients’ expectations focusing on competencies and confidence. | Survey | Personal competencies and confidence levels, clinical environment and patient interactions, and curriculum and training provided by the pharmacy school. | Incorporating feedback from standardized persons into curriculum. |
| Friedlander et al., 2024 | Not mentioned | Ability to apply dental knowledge effectively in practice | Surveys and interviews | Clinical skills, confidence, support from colleagues, availability of resources, quality of dental education, and clinical placements | Improved clinical placements, and continuous professional development |
| Graham et al., 2023 | Not mentioned | Readiness approached through self-perceived competence and preparedness to meet professional responsibilities.  (“Being dependent on students' self-perceptions, their capability to adapt and the influence of the context of undergraduate experience”) | Federation of Rural Australian Medical Educators (FRAME) survey. | Educational background, clinical training, personal expectations from the profession. | Enhancing educational curricula, improving clinical training quality, aligning expectations with professional realities. |
| Grant et al., 2017 | Not mentioned | Competence to handle complex social work cases | Surveys and interviews | Emotional intelligence, resilience, support from supervisors, availability of resources, quality of field education, and theoretical grounding | Regular supervision, workshops on case management and practical skills |
| Grimm and Barker et al., 2022 | Bandura's social cognitive theory | Assessed based on self-efficacy in real clinical settings after increased reliance on simulation due to the pandemic. | Casey-Fink Graduate Nurse Experience Survey. | Type and quality of simulation experiences, realism of simulations, reduction in traditional clinical experiences. | Improving quality and realism of simulations, integrating feedback mechanisms, covering critical nursing care. |
| Gruenberg et al., 2021 | Workplace learning | Readiness for advanced pharmacy practice experiences (APPEs) involves possessing the necessary knowledge, skills, and attitudes to perform effectively in clinical settings, including patient care tasks under the supervision of a pharmacist.  (“It is a holistic construct that includes social and behavioral characteristics as well as knowledge and skills that students require to successfully learn in the workplace.”) | Semi-structured focus groups and interviews | Learner characteristics (e.g., self-awareness, initiative, confidence, adaptability, professionalism), participation in workplace activities and understanding of roles, relationship-building with patients, health care providers, and preceptors, and support and orientation practices provided by educators and workplace participants. | Developing personal characteristics through pre-APPE curriculum, aligning core entrustable professional activities with IPPE and APPE curricula, practicing interprofessional communication skills through simulations, and providing consistent orientation and support at APPE sites. |
| Harrison et al., 2020 | Not mentioned | Integration of knowledge, skills, and behavior in a clinical setting. | Clinical assessments and supervisor feedback. | Clinical competence and confidence, hospital policies and support systems, and quality of clinical placements and mentorship. | Enhancing clinical training programs and providing strong mentorship. |
| Haruzivishe and Macherera, 2021 | Not mentioned | Comprehensive clinical skills and professional competence.  (1. “Having a generalist foundation and some job specific capabilities, providing safe client care, keeping up with the current realities of nursing practice, being well equipped with the tools needed to adapt to the future needs of clients, and possessing a balance of doing, knowing and thinking”. 2. “Being competent and having the knowledge, skills and judgment that is required for such role performance”). | Surveys and interviews | Self-confidence, adaptability, supportive work environment, quality of supervision, practical training, and simulation-based learning | Regular mentorship programs, and increased focus on real-world clinical scenarios |
| Harvey et al., 2021 | Indigenous knowledge and postcolonial/ decolonial theory | Preparedness to practice in the Aboriginal and Torres Strait Islander Health Context includes understanding historical and cultural issues, and the ability to provide culturally safe care. | Survey | Previous educational experience in pre-tertiary years, clinical experience with Aboriginal and Torres Strait Islander people, teaching by Aboriginal and Torres Strait Islander peoples, and formal education sessions within the medical course. | Increased placements with Aboriginal and Torres Strait Islander peoples, more educational time with Aboriginal and Torres Strait Islander academics, and incorporating Aboriginal and Torres Strait Islander pedagogies into curricula. |
| Hatzenbuhler and Klein, 2019 | Not mentioned | Readiness conceptualized through practical skills, clinical judgment, and confidence.  (“The need to be prepared to enter clinical practice.”) | Surveys, interviews and observational notes | Supportive learning environment, exposure to real-life nursing scenarios, and mentorship | Focused training on critical thinking, clinical skills, and stress management techniques |
| Hyun et al., 2020 | Benner's stages of clinical competence from novice to expert | Readiness for practice involves achieving expected competency levels across various domains, including clinical skills, professional behavior, and the ability to apply theoretical knowledge in practical settings. | Surveys and competency assessments | Quality of nursing education and training programs, clinical placements and practical experience, support from mentors and supervisors, and individual attributes such as confidence, communication skills, and adaptability. | Improving the quality and structure of clinical placements, providing continuous support and mentorship, regular assessment and feedback mechanisms, and enhancing educational programs to bridge theory and practice. |
| Illing et al., 2013 | Constructivist grounded theory | Capability to perform clinical tasks and integrate into professional roles | Interviews and focus groups | Confidence, clinical skills, support from colleagues, workload, quality of medical education, and clinical exposure | Enhanced mentorship programs, increased practical training and real-world scenarios |
| James and Cole, 2016 | Not mentioned) | Assessing intern pharmacists’ perceived preparedness for practice. | Questionnaire | Interns' confidence in performing pharmacy-related activities, desire for patient interaction, location of internship (urban), involvement in community and hospital settings, limited multidisciplinary team care, and undergraduate training focused on product-centered skills, limited clinical training, and new revised curriculum aimed at increasing clinical skills. | Incentives for interns, improved clinical exposure, enforcement of pharmacy regulations, inclusion of business management topics in the curriculum. |
| Jamieson et al., 2019 | Duchscher’s theory of Transition Shock | Ability to effectively transition from student to professional nurse roles.  (“The ability as a graduate nurse, to assume the roles of a provider of care, designer/ manager/ coordinator of care, and member of the nursing profession”). | Casey-Fink Readiness for Practice Survey and focus groups | Self-confidence, clinical skills, support from mentors, clinical environment, quality of nursing education, and practical training | Increased clinical placements, and enhanced mentorship programs |
| Javed et al., 2023 | Not mentioned | Clinical competencies and confidence in practice. | Dental Undergraduates Preparedness Assessment Scale | Clinical experience and confidence levels, clinical practice settings and support systems, and quality of dental education and clinical training. | Strengthening clinical training programs and continuous professional development. |
| Kasita et al., 2023 | Not mentioned | Competence to perform radiographic duties competently and independently | Interviews | Clinical skills, confidence, support from colleagues, quality of equipment, practical training, and curriculum relevance | More hands-on training, and enhanced mentorship programs |
| Kinnane et al., 2021 | Not mentioned | Readiness for practice involves having the necessary technical skills, clinical competence, and professional behaviors required for sonography practice. | Surveys and interviews | Technical skills and knowledge, clinical competence, professional behaviors and attitudes, and support from clinical supervisors and mentors. | Improving technical and clinical training, providing continuous mentorship and support, fostering professional behaviors and attitudes, and regular assessment and feedback. |
| Kuzmenko et al., 2023 | Personal characteristics, readiness to perform tasks, and contextual factors | Includes clinical skills, decision-making abilities, and professional behavior | Surveys and interviews | Clinical knowledge, interpersonal skills, quality of clinical placements, institutional support, comprehensive curriculum, and practical exposure | Structured clinical rotations, enhanced mentorship and support systems |
| Lagali-Jirge and Umarani, 2014 | Not mentioned | Readiness conceptualized as the ability to independently handle dental procedures and interact with patients | Self-assessment surveys and feedback from supervisors | Educational environment, quality of teaching, and access to practical experiences | Enhanced curriculum focusing on practical skills, increased patient interaction |
| Lanahan et al., 2022 | Not mentioned | Readiness for practice during the COVID-19 pandemic involves the ability to adapt to rapidly changing clinical environments, manage stress, and apply theoretical knowledge under pressure. | Surveys and interviews | Psychological resilience, support from faculty and peers, access to adequate personal protective equipment, and adaptability to new protocols and guidelines. | Increased mental health support, enhanced training on infection control and use of PPE, flexible and adaptive learning environments, and continuous updates on changing protocols and guidelines. |
| Lazarus et al., 2021 | Not mentioned | Readiness to practice is linked to the willingness to volunteer and gain practical experience in real-world medical situations. | Surveys and interviews | Willingness to volunteer, practical experience gained through volunteering, support from mentors and peers and educational background and training. | Encouraging volunteer opportunities, providing support and mentorship for volunteers, integrating volunteer experiences into the medical curriculum and continuous assessment and feedback. |
| Leufer and Cleary-Holdforth, 2020 | Not mentioned | Readiness for practice involves having sufficient clinical skills, confidence, and theoretical knowledge to handle real-world nursing situations. | Surveys and interviews | Prior clinical experience, mentorship and guidance from experienced nurses, quality of the nursing program, and exposure to a variety of clinical settings. | Increased clinical practice opportunities, structured mentorship programs, enhanced simulation-based training, continuous feedback and assessment mechanisms. |
| Li et al., 2022 | The operational conceptual framework of work readiness, which encompasses work competence, social intelligence, organizational acumen, and personal work characteristics | Work readiness encompasses work competence, social intelligence, organizational acumen, organizational hierarchy and personal characteristics necessary to perform effectively and confidently in the nursing profession. | Surveys and assessments | Clinical skills and knowledge. Support from colleagues and supervisors. Work environment and organizational culture. Educational preparation and training. | Providing comprehensive training programs, offering continuous support and mentorship, fostering a positive work environment, and regular assessment and feedback. |
| Lim et al., 2024 | Duchscher's conceptual framework on the three stages of role transition, and Benner's theory on the development of nursing competence over two years. | Ability to handle clinical responsibilities and integrate into roles.  (“Work readiness encompasses four elements: individual characteristics (e.g., ability to handle daily tasks and challenges encountered in the workplace), clinical characteristics (e.g., clinical competency, problem-solving and independent decision making), interpersonal characteristics (e.g. communication and teamwork), and organizational elements.”) | Surveys and performance evaluations. | Previous clinical experience and self-confidence, workplace support and orientation programs, and adequacy of nursing education and clinical training. | Structured orientation programs and ongoing professional development. |
| Mak et al., 2013 | Not mentioned | Ability to perform pharmacy-related activities and confidence. | Surveys | Personal skills and confidence levels, clinical environment and support systems, and quality of pharmacy education and internship training. | Improving internship programs and providing more clinical exposure. |
| Malau-Aduli et al., 2022 | Not mentioned | Ability to transition from student to professional roles across health professions.  (“Readiness for practice is an emerging field of educational research which focuses on graduating students’ work-readiness, and the identification and management of factors such as student familiarity with teaching curricula, assessment, placement expectations and an understanding of professional requirements after graduating, which influence transition into the workforce.”) | Surveys and focus groups | Clinical skills, confidence, support from clinical staff, workload, quality of education, and practical training | Enhanced practical training and placements, and supportive mentorship programs |
| Mariño et al., 2022 | Not mentioned | Readiness to perform clinical duties and manage patient care independently.  (“Having adequate technical skills, clinical knowledge and attributes necessary to practice their chosen profession”) | Interviews | Confidence, clinical skills, support from colleagues, quality of supervision, practical training, and simulation-based learning | Enhanced mentorship and supervision, and continuous professional development programs |
| Meyer and Shatto, 2018 | Conner and Davidson's definition of resilience, Wagnild and Young's five characteristics of resilience, Grafton et al.'s three "waves" of resilience, and Hodges et al.'s framework for building professional resilience | Includes resilience and the ability to transition effectively into practice | Surveys and interviews | Resilience, adaptability, support from colleagues, work environment, curriculum design, and practical experiences | Programs to build resilience, supportive clinical environments |
| Missen et al., 2015 | Not mentioned | Work readiness involves having mastery of the necessary clinical skills, knowledge and professional attitudes to effectively transition into the nursing profession. | Surveys and interviews | Quality of nursing education, clinical training and practical experiences, support from mentors and supervisors, and organizational culture and work environment. | Enhanced clinical training programs, structured mentorship and support, continuous professional development, regular assessment and feedback mechanisms. |
| Monrouxe et al., 2018 | Self-efficacy, self-concept, metacognition, cognition, models of expert performance, reflective practice, reflection-on-practice, reflection-in-practice | Capacity to integrate into clinical practice effectively.  (Possessing the knowledge, skills and behaviors expected of graduates, and include knowing limitations, prioritization, managing stress, engendering patient trust and generally being a safe doctor). | Interviews | Clinical knowledge, interpersonal skills, institutional support, mentoring, quality of medical education, and clinical exposure during training | Structured mentorship programs, and enhanced clinical exposure during training |
| Muruvan et al., 2021 | Phenomenology | Ability to transition from education to professional practice smoothly | Surveys and interviews | Clinical skills, confidence, support from employers, availability of resources, quality of dental education, and practical training | Increased practical training and clinical exposure, continuous collaboration between educational institutions and industry |
| Musallam and Flinders, 2021 | Duchscher & Windey’s Stages of Transition and Transition Shock model | Readiness for clinical practice involves confidence, comfort, and the ability to apply theoretical knowledge in clinical settings.  (1. “Having a generalist foundation with specific capabilities related to the job, an ability to provide safe client care, awareness of the current state of nursing practice, being well-equipped with the necessary tools to adapt to the future client needs, and the possession of balance between doing, knowing, and thinking.” 2.) “Being competent and having the knowledge, skills, and judgment required for one’s role.”) | Survey | Clinical experience, support from faculty and mentors, quality of clinical placements and integration of theory and practice. | Enhanced simulation experiences, improved quality and duration of clinical placements, increased support from faculty and clinical mentors. |
| Mustakallio et al., 2020 | Association for Dental Education in Europe (ADEE) guidelines for the profile and competencies of graduating European dentists | Professional competence and readiness for independent practice. | Surveys | Clinical skills and self-perceived competence, healthcare system and clinical practice environment, and dental education quality and clinical exposure. | Improving clinical training and providing hands-on practice. |
| Nelson et al., 2023 | First Nations' methodology, Working at the research interface | Includes cultural competence and clinical skills for working with First Nations Australians | Surveys and focus groups | Cultural awareness, clinical skills, support from colleagues, understanding of community needs, training on cultural competence, and practical experience in relevant settings | Culturally specific training modules, and increased exposure to clinical settings involving First Nations communities |
| Nweke et al., 2021 | Not mentioned | Readiness to resume clinical practice during the COVID-19 pandemic | Surveys | Confidence, knowledge of infection control, institutional support, availability of PPE, training on infection control, and practical experiences | Training on infection control, and provision of adequate PPE |
| O'Brien et al., 2013 | Not mentioned | Preparedness to handle the practical and theoretical aspects of paramedic work.  (“Preparedness for a lifetime working within a field that is likely to change significantly over the lifespan of the graduate”). | Survey | Practical skills, theoretical knowledge; quality of clinical placements, interaction with experienced paramedics; course content, hands-on training. | More varied clinical placements, increased focus on practical skills |
| O'Brien et al., 2020 | Not mentioned | Work readiness involves possessing the necessary personal characteristics, skills, and attitudes to effectively perform in a professional allied health setting. | Surveys and interviews | Personal characteristics such as resilience, adaptability, and communication skills, contextual factors including workplace culture and support, and educational background and training. | Development of personal skills through targeted training programs, supportive workplace environments, and comprehensive educational curricula that include practical experiences. |
| Oluwatosin and Ogundero, 2021 | Not mentioned | Readiness is seen as a combination of theoretical knowledge and practical skills, alongside self-assessment of readiness | Online semi-structured questionnaires | Infrastructure, practical training exposure, and personal interest in the field | Strengthening infrastructure, enhancing practical training, and encouraging self-assessment |
| Opoku et al., 2021 | Interpretive phenomenology | Transition readiness through experiences and challenges faced. | Interviews and surveys. | Personal resilience and adaptability, support systems and clinical placement experiences, and quality of training and mentorship. | Enhanced mentorship programs and support networks. |
| Ottrey et al., 2021 | Social constructionism | Conceptualized through experience, skills, confidence, knowledge, competence, self-sufficiency, accountability and employability.  (“knowledge, skills (e.g. prioritization, safety), self-awareness (i.e. good  understanding of yourself such as knowing limitations) and resilience  (e.g. dealing with psychological distress)”) | Interviews | Work environment, support systems, personal motivation, and prior experiences | Support networks, continued professional development, and reflective practices |
| Phan et al., 2023 | Not mentioned | Conceptualized as the transition from knowledge acquisition to practical application | Surveys and direct observation by supervisors during placements | Educational support, access to varied clinical placements, and personal resilience | Improving access to diverse clinical placements and mentorship programs |
| Phillips et al., 2023 | Meleis' theory of transition | Being able to transition into practice amidst challenging circumstances such as the COVID-19 pandemic | Surveys | Resilience, adaptability, support from healthcare institutions, availability of PPE, adaptations in curriculum, and simulation-based learning | Enhanced support systems during transitions, and simulation-based training for pandemic scenarios |
| Piccuito and Santiago, 2023 | Transition-to-practice programs | Being able to transition smoothly from an educational setting to professional practice | Surveys and interviews | Personal coping mechanisms, adaptability, institutional support, availability of resources, clinical placement experiences, and mentorship during training | Extended clinical placements, and comprehensive orientation programs |
| Powers et al., 2021 | Not mentioned | Capacity to handle real-world clinical situations | Surveys and interviews | Adaptability, stress management, impact of pandemic on training, availability of resources, quality of online learning, and clinical placement availability | Increased simulation-based learning, and enhanced support systems |
| Pullen and Ahchay, 2022 | Benner's stages of learning and expertise development | Multifaceted concept encompassing transition from university to practice focusing on preparedness in a hospital-based transition program. | New Graduate Nurse Workplace Preparation Survey. | Diversity and timing of clinical placements, support during placements, nature of pre-placement preparations. | Enhancing clinical placements, improving access to clinical skills labs, providing comprehensive orientation. |
| Reynolds and Mclean, 2021 | Benner's stages of clinical competence from novice to expert | Readiness conceptualized as acquiring a range of competencies through staged skill acquisition from novice to expert | Semi-structure interviews | Quality of clinical supervision, diversity of clinical cases, and educational curriculum | Structured skill progression, regular feedback, and competency-based evaluations |
| Rusch et al., 2019 | The 3 domains of learning framework (affective, psychomotor, and cognitive) | Includes critical thinking, clinical judgment, and technical skills | Surveys | Clinical confidence, professional behavior, quality of preceptorship, institutional support, integration of clinical and theoretical learning, and simulation-based education | Strengthening academic-clinical partnerships, increasing focus on time management and prioritization skills |
| Shaw et al., 2018 | Not mentioned | Capacity to transition from student to professional roles, focusing on competencies like critical thinking and time management | Surveys and interviews | Critical thinking, time management, quality of preceptorship, institutional support, practical training, and simulation-based learning | Enhanced focus on critical thinking and time management in curricula, and strengthening preceptor training programs |
| Sheehan et al., 2018 | The Professional Capability Framework | Readiness framed within the context of transitioning from medical school to junior doctor roles, focusing on practical and personal development aspects. | National surveys | Undergraduate experiences, particularly rural clinical placements, shaping readiness. | Promoting rural placements that offer extensive patient responsibility and immersive experiences to boost confidence and competence. |
| Smith et al., 2021 | Not mentioned | Readiness affected by changes and challenges due to COVID-19. | Surveys and interviews. | Adaptability and resilience, workplace changes and support during the pandemic, and adjustments in training and education due to the pandemic. | Providing additional support and training during crises. |
| Sterner et al., 2023 | Not mentioned | Emphasis on the gap between theoretical knowledge and practical skills needed in the workplace.  (“Work readiness encompasses four different domains: work competence, personal work characteristics, organizational acumen, and social intelligence.”) | Survey | Practical skills, adaptability, workplace support, supervision quality, practical training, and curriculum alignment | More practical placements, and enhanced supervision during initial work periods |
| Stoikov et al., 2022 | Duchscher's theoretical framework of new graduate transition stages in nursing | Readiness for practice involves the ability to effectively transition from student to professional, applying clinical skills and adapting to the demands of the physiotherapy profession. | Surveys and interviews | Clinical placements and practical experience, support from clinical educators and mentors, quality of the educational program, and opportunities for hands-on practice and skill development. | Enhanced clinical placement experiences, structured mentorship programs, continuous professional development and training, and integration of practical skills with theoretical knowledge. |
| Stulz et al., 2023 | Woman-centered care and authentic learning | Focus on the transition of midwifery students, emphasizing continuity of care experiences as a core component of readiness. | In-depth interviews | Extent and quality of student-led practice experiences, mentorship during these practices, curriculum alignment. | Enhancing the student-led practice model, providing robust mentorship programs, aligning curriculum with practical needs. |
| Tarhan et al., 2022 | Not mentioned | Work readiness involves the ability to effectively collaborate with colleagues, apply clinical skills, and adapt to the demands of the nursing profession. | Surveys and interviews | Collaboration with colleagues, clinical competence, support from the nursing team and supervisors, and quality of the nursing education program. | Promoting teamwork and collaboration in the workplace, continuous professional development and training, structured mentorship programs, regular feedback and assessment. |
| Thomas et al., 2023 | Not mentioned | Bridging the gap between theoretical knowledge and practical application | Surveys | Critical thinking, adaptability, supportive environment, availability of mentors, quality of educational programs, and practical training opportunities | Implementation of nurse residency programs, and continuous curriculum evaluation |
| Usher et al., 2015 | Benner's "novice to expert" theory | Ability to apply theoretical knowledge in clinical settings, enhanced by completing a capstone subject | Surveys | Self-confidence, previous clinical experience, support from mentors, clinical environment, quality of the capstone subject, and practical training components | Implementation of capstone subjects, increased clinical placements and mentorship |
| Waite et al., 2018 | Not mentioned | Ability to provide comprehensive pharmacist services independently | Surveys and interviews | Clinical knowledge, communication skills, institutional support, access to resources, practical training, and alignment of curriculum with professional requirements | Enhanced clinical placements, continuous professional development opportunities |
| Walker and Campbell, 2013 | The work readiness scale developed by Caballero et al. (2011), which conceptualizes work readiness as a multidimensional construct comprising four factors: organisational acumen, social intelligence, personal work characteristics, and work competence. | Work readiness comprises clinical competence, organizational acumen and social intelligence necessary to perform effectively in nursing roles and influence job satisfaction and work engagement.  (“Graduate work readiness is the extent to which graduates are perceived to possess the skills and attributes that render them prepared for success in the workplace and is increasingly being recognized as indicative of graduate potential in terms of job performance and career advancement.”) | Work Readiness Scale and interviews | Clinical competence and skills, support from colleagues and supervisors, work environment and organizational support, and quality of nursing education and training. | Providing comprehensive training and development programs, offering mentorship and continuous support, creating a positive and supportive work environment, and regular assessment and feedback. |
| Walker et al., 2013 | Not mentioned | Work readiness involves social intelligence, organizational acumen,  work competence and resilience to effectively transition into the health professional workforce.  (“Work readiness is the extent to which graduates possess the attributes that prepare them for success in the workplace”) | Surveys and interviews | Clinical skills and practical knowledge, support from mentors and supervisors, work environment and organizational culture, and quality of educational preparation and training. | Enhancing clinical and practical training, providing mentorship and continuous support, creating a supportive work environment, and regular assessment and constructive feedback. |
| Walters et al., 2022 | Work readiness | Work readiness is the extent to which new graduates possess qualities and competencies associated with workplace success, including clinical knowledge and skills, job satisfaction, and work engagement.  (“Readiness for clinical practice refers to new graduates' confidence and comfort in applying their nursing knowledge, skills, and values.”) | The Work Readiness Scale–Graduate Nurse | Demographic characteristics (age, gender, race), type of nursing degree, completion of a nurse externship program and previous healthcare work experience. | Participation in nurse residency programs, externship programs for gaining clinical experience, continuous support and mentorship, and structured training and development programs. |
| Watt and Pascoe, 2013 | Not mentioned | Integration of theoretical and clinical training, focusing on developing close relationship between academic education and practical application. | Interviews | Quality of undergraduate clinical experience. Clinical placement quality, sense of belonging in clinical environment, transition from academic settings to real-world nursing environments. | Improving integration of educational and practice settings, providing meaningful clinical experiences, fostering close collaboration between educational institutions and healthcare settings. |
| Wells et al., 2021 | Workplace learning framework | Work readiness involves having the necessary clinical, business, and employability knowledge and skills to effectively transition into private practice settings, including the ability to handle autonomous clinical reasoning, caseload management, and business and administrative responsibilities.  (“The degree to which graduates possess characteristics and attributes that prepare them for transition and success in the workplace.”) | Surveys and focus groups. | Clinical placements and private practice experience, employer and client expectations, workplace support and mentorship, university academic preparation and continuing education, and individual attributes such as confidence, communication skills, and readiness to learn. | Increasing private practice placements for students, providing workplace support such as mentoring and professional development, including business and administrative skills in university curricula, encouraging continuing education and postgraduate programs tailored to new graduates. |
| Wijnen-Meijer et al., 2015 | Vertical integration of medical curricula | Readiness to practice is achieved through a vertically integrated medical education approach that emphasizes continuity of learning and progressive responsibility in clinical settings. | Surveys and qualitative interviews | Continuity of learning experiences, progressive responsibility in clinical practice, integration of theoretical knowledge with practical application, and support from mentors and clinical educators. | Implementation of vertically integrated medical education models, continuous mentorship and support throughout the educational journey, progressive increase in clinical responsibilities and integration of theoretical and practical learning experiences. |
| Willman et al., 2020 | Benner's theory of nursing expertise development, and the EU Directive 2013/55/EU on the importance of lifelong learning for registered nurses. | Self-assessment of clinical competence | Questionnaires and interviews | Self-confidence, clinical skills, supportive work environment, access to resources, practical training during nursing education, and simulation-based learning | Continued professional education, and simulation-based training sessions |
| Wong et al., 2023 | Not mentioned | Work readiness involves possessing the necessary competencies, personal characteristics and organizational acumen required to perform effectively in pharmacy roles. | Surveys and interviews | Quality of pharmacy education, clinical and practical training experiences, support from mentors and preceptors, and organizational culture and work environment. | Enhanced clinical and practical training, structured mentorship and support programs, continuous professional development and training, and regular assessment and feedback mechanisms. |
| Woods et al., 2015 | Benner's novice-to-expert model of skill acquisition, and the Nursing and Midwifery Board of Australia (NMBA) standards for nursing registration. | Readiness to practice involves confidence, clinical competence, and the ability to integrate theoretical knowledge into practical settings. | Casey-Fink Readiness for Practice Survey | Quality of clinical placements, support from mentors and faculty, hands-on practice opportunities, and the integration of theory and practice in the curriculum. | Improved clinical placement experiences, increased mentorship and support from faculty, enhanced practical training, and continuous assessment and feedback. |
| Woolley et al., 2019 | Social accountability in health professional education (SAHPE) | Readiness seen as the outcome of education that aligns with community health needs | Surveys and students assessments | Curriculum relevance, community engagement during training, and personal commitment | Integrating community health needs into curriculum, promoting active learning |
| Zhang et al., 2023 | Not mentioned | Work readiness described through self-assessment of skills and knowledge, emphasizing multidimensional aspects of professional competence in a rapidly changing environment. The extent to which a person possesses specific skills, knowledge and attributes.  (“A contextual multidimensional construct centered around potential success in the work environment. It is indicated by the extent to which a person possesses specific skills, knowledge and attributes”) | Interviews | Clinical placements as students, workplace familiarity, gradual immersion, understanding and nurturing teams, peer support networks, and personal coping strategies. | Providing supportive collegial environments, structured orientations, and individualized attention during the transition to the workplace. |
